# Finite element human body models with active reflexive muscles suitable for sex based whiplash injury prediction

**DOI:** 10.3389/fbioe.2022.968939

**Published:** 2022-09-29

**Authors:** I Putu Alit Putra, Johan Iraeus, Fusako Sato, Mats Y. Svensson, Robert Thomson

**Affiliations:** ^1^ Injury Prevention Unit, Division of Vehicle Safety, Department of Mechanics and Maritime Sciences, Chalmers University of Technology, Gothenburg, Sweden; ^2^ Japan Automobile Research Institute (JARI), Tsukuba, Japan

**Keywords:** whiplash, finite element, human body models, reflexive neck muscle, rear impact

## Abstract

Previous research has not produced a satisfactory resource to study reflexive muscle activity for investigating potentially injurious whiplash motions. Various experimental and computational studies are available, but none provided a comprehensive biomechanical representation of human response during rear impacts. Three objectives were addressed in the current study to develop female and male finite element human body models with active reflexive neck muscles: 1) eliminate the buckling in the lower cervical spine of the model observed in earlier active muscle controller implementations, 2) evaluate and quantify the influence of the individual features of muscle activity, and 3) evaluate and select the best model configuration that can be used for whiplash injury predictions. The current study used an open-source finite element model of the human body for injury assessment representing an average 50th percentile female anthropometry, together with the derivative 50th percentile male morphed model. Based on the head-neck kinematics and CORelation and Analyis (CORA) tool for evaluation, models with active muscle controller and parallel damping elements showed improved head-neck kinematics agreement with the volunteers over the passive models. It was concluded that this model configuration would be the most suitable for gender-based whiplash injury prediction when different impact severities are to be studied.

## 1 Introduction

Whiplash injuries occur frequently around the world. In Sweden, from 2000 to 2009, an overall annual incidence of 235 per 100,000 population (Styrke et al., 2012) was observed. Similarly, 328 people were treated with neck sprain per 100,000 population in the US (Quinlan et al., 2004). Claims for whiplash injuries was relatively high (417 per 100,000 population) in Saskatchewan, Canada (Cassidy et al., 2000). Despite the high incidences of whiplash injuries and their excessive socio-economical cost worldwide (Gisolf-Berecki et al., 2013; Styrke et al., 2012; Quinlan et al., 2004; Cassidy et al., 2000), whiplash injuries’ aetiology remains unclear.

Many hypotheses of how whiplash injuries occur are connected to the retraction phase of the neck (Svensson et al., 1993; Grauer et al., 1997; Ono et al., 1997; Yoganandan et al., 2000; Ono et al., 2006). The retraction phase occurs when the relative motion of the head and torso produces an “S” shape in the cervical spine and is most common when the occupant’s vehicle is hit by a vehicle from behind. Thus, any surrogate tools (for example, crash test dummies or human body finite element models) that are typically used to study the kinematics of whiplash injuries should be able to replicate this S-like retraction motion. Several injury sites in the neck have been proposed that are related to whiplash which includes spinal ligaments, intervertebral discs, vertebral arteries, dorsal root ganglia, and neck muscles (Siegmund et al., 2009).

Injury statistics have shown that females have a higher risk for injuries and fatalities in traffic (Forman et al., 2019) with the highest difference in risk related to whiplash injuries sustained in low severity vehicle crashes (Carlsson et al., 2011). Another study by Kullgren et al. (2013) also concluded that whiplash protection in seats monitored on the Swedish market were less effective for females than males.

Finite Element (FE) Human Body Models (HBMs) have been a powerful and essential tool when developing and assessing road user safety. Until recently, FE HBMs that represent average female anthropometry did not exist. To fill this gap, an open-source 50th percentile female HBM called VIVA OpenHBM was developed previously (Östh et al., 2017a; 2017b) and validated against Post-Mortem Human Subject (PHMS) responses in rear impacts. It was further improved by adding active neck muscle controllers and muscle Co-Contraction (CCo) (Putra et al., 2019, 2020, 2022) since neck muscle activities have been shown to influence the head-neck kinematics during whiplash-like rear-impact volunteer tests (Brault et al., 2000; Siegmund et al., 2003; Blouin et al., 2006; Siegmund, 2011; Dehner et al., 2013; Mang et al., 2015). Muscle CCo was added in Putra and Thomson. (2022) since it has been known that muscle CCo contributes to maintaining spinal stability (Le et al., 2017) and directly keep the head in an upright position under gravitational acceleration.

With active muscle controllers and muscle CCo, the VIVA OpenHBM kinematics were improved over a passive neck implementation when compared to volunteer responses. However, several limitations were observed in those studies. Undesirable oscillations and buckling in the lower cervical spine (C4–C6) due to the active muscle controller were observed. It was hypothesized that this buckling could be due to the limitations of the current Hill muscle implementation in LS-DYNA itself. As mentioned in Kleinbach et al. (2017), the current implementation of the LS-DYNA Hill muscle only includes a parallel damping element (PDE) and neglects serial elastic and damping contributions of tendon structures. Subsequently, instabilities (oscillations and sudden drop in the active force generation) produced by force-velocity or force-length relation formulation (Wittek et al., 2000), incorrect energy storage and release in the interaction with the environment (Mörl et al., 2012), and unrealistic high-frequency oscillations (Günther et al., 2007) can arise. In addition, the muscle model in VIVA OpenHBM was modeled as 1D Truss elements and may not reproduce damping and stiffness effects from 3D muscles containing the vertebrae (Hedenstierna and Halldin, 2008). Therefore, it is desirable to explore numerical methods to eliminate the buckling in the lower cervical spine and better represent cervical kinematics for injury prediction.

A more sophisticated and robust open-source model called VIVA+ has been developed (John et al., 2022a). Its neck model is based on the VIVA OpenHBM’s neck. With improvements like a true average anthropometry, improved cervical spine curvature, updated soft tissue modelling, and better modelling of other body parts, it is strategic to use the VIVA+ model for simulating whiplash injury cases. However, no active muscle controller was developed for the VIVA+ model. In addition, the roles of each feature in an active muscle controller should be determined to identify their influence on head-neck kinematics that influence the calculation of whiplash injury criteria. For example, the primary inputs for global injury criteria such as Neck Injury Criteria (NIC) (Boström et al., 2000) are head and T1 centre of gravity (CG) x-accelerations. For indirect tissue-based injury assessment, such as analyzing transient pressure gradients in the spinal canal (Svensson et al., 1998; Yao et al., 2016), the individual vertebral angular displacements of the cervical spine are input into the calculation.

Based on the model limitations and research gaps explained previously, three objectives were addressed in the current study: 1) eliminate the buckling in the lower cervical spine of the model observed in earlier active muscle control implementations, 2) evaluate and quantify the influence of the individual features of muscle activity, and 3) evaluate and select the best model configuration that can be used for whiplash injury predictions.

## 2 Material and methods

**FIGURE 1:**
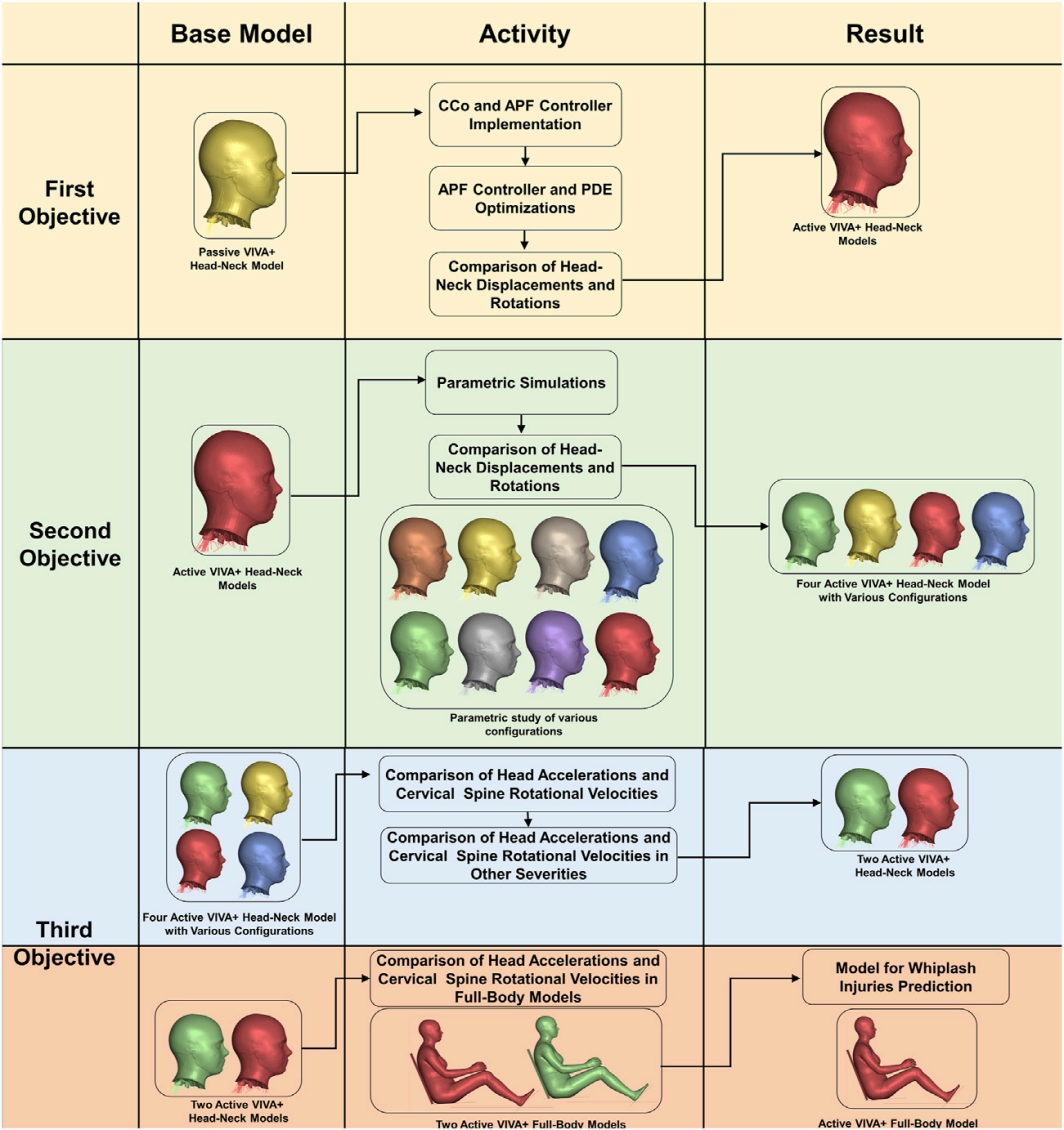
Flow chart of the methods connected to the present Study’s objectives.

The overall methods used in the present study are described in a flowchart shown in Figure 1. Both female and male VIVA+ models (PSV) were used, although the female VIVA+ is shown in the flowchart. In a first step, an active muscle controller called Angular-positioned Feedback (APF) controller and Muscle CCo level from the Active VIVA OpenHBM were implemented in the head-neck of the VIVA+ model. Optimization simulations (Table 1) were performed to determine the Proportional Derivative (PD) coefficients for the APF controller and optimize the PDE coefficient of the Hill muscle model. Head-neck kinematic comparisons against volunteer responses were also conducted.

**TABLE 1:** Optimization parameter.

Parameter	Symbol	Unit	Initial value	Optimization range
Proportional gain	KPA	%contraction/rad	6	0.01-100
Derivative gain	KDA	%contraction/rad ms^-1^	5	0.01-100
Neural transmission and processing delay	TNDA	ms	20	3.5–20
Parallel Damping Element Coefficient	PDE	kN.ms/mm^2^	0.02	0.01-0.05

To address the second objective, parametric simulations using the model with the highest CORelation and Analysis (CORA) score from the previous step (Table 2) were conducted to analyze the effects of muscle CCo, muscle PDE, and APF controllers on the model’s head and neck kinematics. The four best models (based on CORA score evaluation) were chosen from the eight available models. In each subsequent step, the number of models were halved.

**TABLE 2:** Parametric simulations of model configuration.

Name of simulation	CCo	PDE	APF controller	Notes
PSV + PDE + CCo + APF	yes	yes	yes	The model with the highest CORA score as the result from the first objective
PSV + PDE + APF	no	yes	yes	
PSV + CCo + APF	yes	no	yes	
PSV + CCo + PDE	yes	yes	no	
PSV + APF	no	no	yes	
PSV + PDE	no	yes	no	
PSV + CCo	yes	no	no	
PSV	no	no	no	Original model based on John et al., 2022a; 2022b

The four best models were analyzed further to address the third objective of the present study. The models were analyzed by comparing the models’ cervical spine rotational velocity and head C.G x-linear acceleration to the responses of three different volunteer datasets. This yielded two models with the best agreement with volunteer kinematics. These two models were evaluated further by conducting simulations in the full-body model. Finally, one model (for each gender) with the best agreement to the head and T1 C.G x-acceleration and cervical vertebra rotational velocity was selected. This model was considered appropriate for whiplash injury prediction simulations.

### 2.1 Base model

**FIGURE 2:**
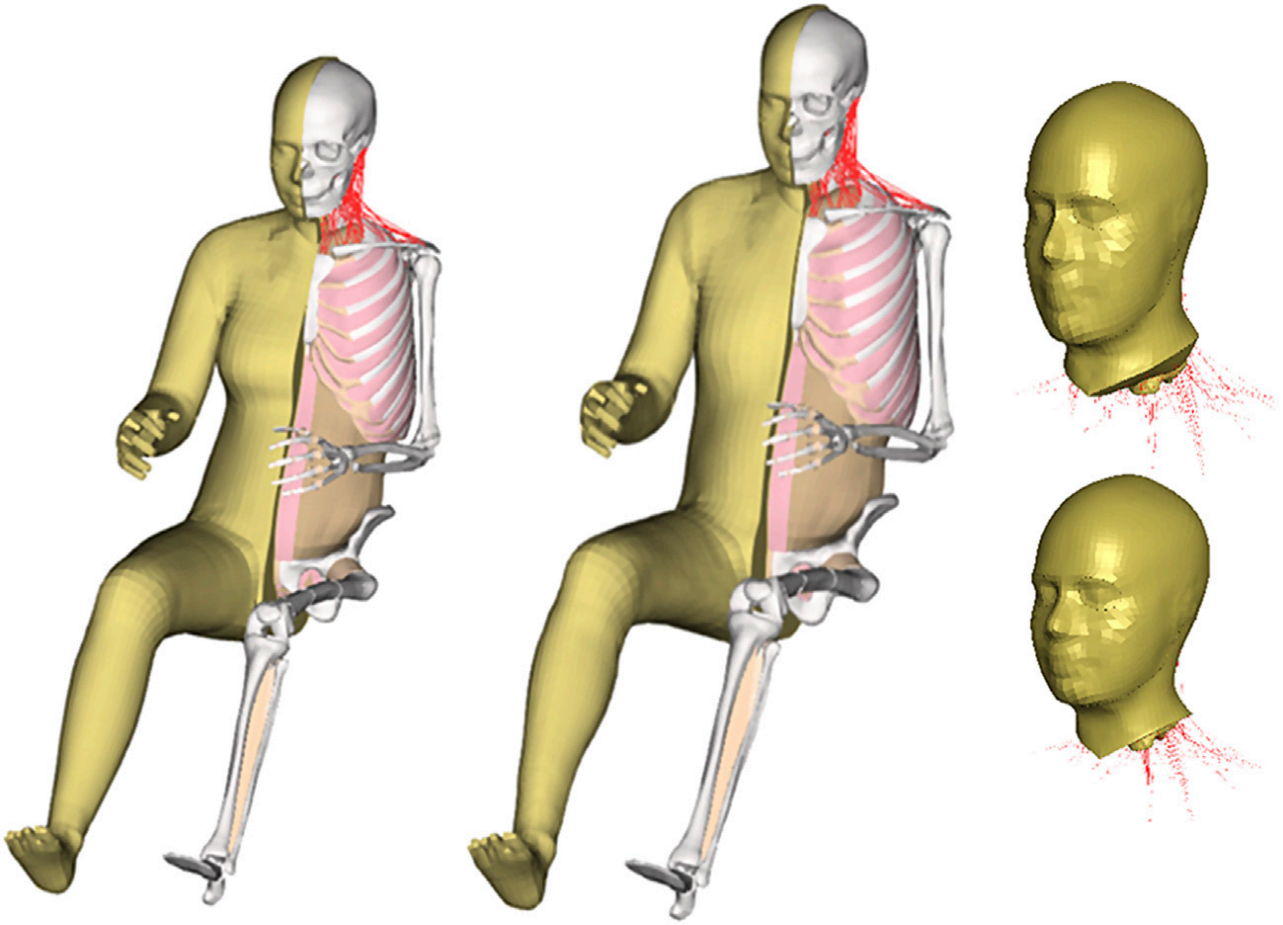
VIVA+ finite element human body models and isolated VIVA+ head-neck models.

The baseline VIVA+ FE HBM is an average 50th percentile female model (Figure 2). This model was morphed to create a derivative model of an average 50th percentile male. The derivative model has identical elements with the baseline model but the nodal coordinates were adjusted according to several statistical shape models describing the outer body shape, ribcage, femur, tibia and pelvis (John et al., 2022a). Besides changes in geometry to develop the average male from an average female, several properties were also updated. These properties included head mass and inertia properties, densities of soft tissues, as well as knee ligament and quadricep muscle properties (John et al., 2022a). The original, passive, VIVA+ models without active neck muscle responses were validated to blunt impacts in different directions (frontal, lateral and back) (John et al., 2022a; 2022b). Specifically, multi-level kinematics validations were conducted for the rear-end impact collision starting from the functional spinal unit (FSU) level, isolated head-neck level, and whole-body level. The details of VIVA+ HBMs developments and validation are described further in John et al. (2022a; 2022b). In the present study, sub-models that consist only of the head-neck were created by cutting both average female and male models above the first thoracic vertebrae (T1).

### 2.2 Reference volunteer data

Models were compared to the reference volunteer data of Sato et al. (2014) and used separately for different sexes. The data of Sato et al. (2014) consisted of two sled test series. The first test series was conducted with twelve males and eight female volunteers seated in a rigid seat (seatback angle was 20° from vertical) without a headrest. Rear-impact tests with delta velocities of 8.1 km/h and 10 km/h were conducted using an inclined sled rail with a 10-degree inclination. The head C.G x-accelerations and T1 C.G displacements data (linear x- and z-displacements), as well as head C.G y-rotational displacement, from Sato et al. (2014) first test series were used for comparison in the present study and were referenced as “Sato et al. (2014) 8.1 km/h”, and “Sato et al. (2014) 10 km/h”

The second volunteer test series of Sato et al. (2014) was based on a low-speed rear-impact test conducted using a mini sled test with a delta velocity of 5.8 km/h. Four male volunteers and two female volunteers were seated in a rigid seat (seatback angle 20° vertical) without a headrest. In the current study, the second test series of Sato et al. (2014) was referenced as “Sato et al. (2014) 5.8 km/h”. Head C.G displacements and accelerations, T1 C.G displacements and C1-C7 y-rotational displacement and velocity data from that test series were used in the present study to calibrate and compare the VIVA+ FE models kinematics.

### 2.3 Simulation setup

**FIGURE 3:**
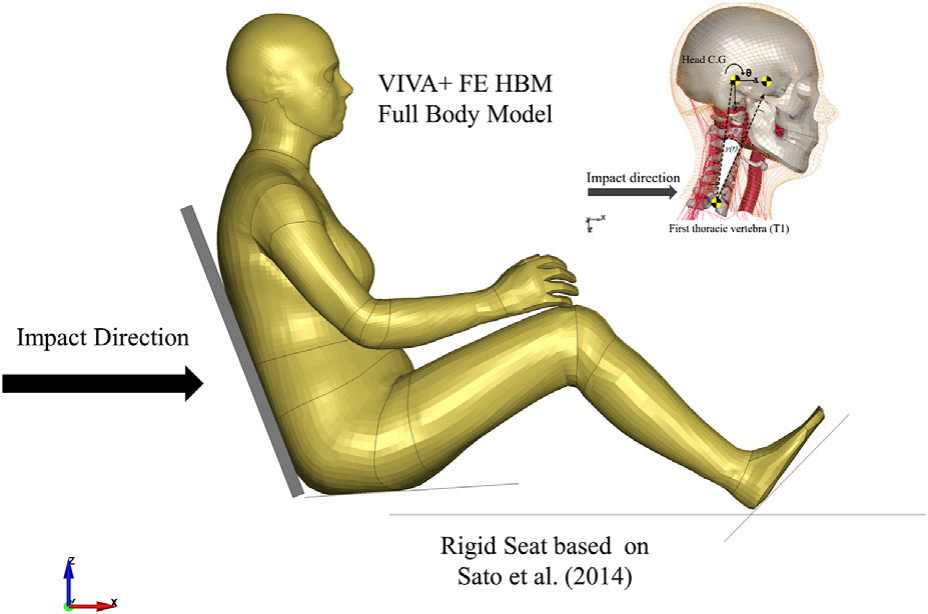
Simulations Setup of VIVA+ Female and Male Head-Neck and Full Body Model based on Sato et al. (2014). Implementation and optimization of Angular-positioned Feedback (APF) controller and parallel damping element (PDE).

The head-neck model was simulated by prescribing the volunteer T1 kinematics on the T1 of the model (Figure 3). The lower nodes of the skin and several nodes in the soft tissues were constrained to move with T1. The termination time of each simulation was 450 ms that includes 150 ms of initial quasi-static equilibrium settling to gravitational acceleration. Full model simulations of both female and male VIVA+ models were done following the Sato et al. (2014) setup (Figure 3). The two models were seated in a rigid seat with a 20-degree seatback angle from the vertical. Sled acceleration from the experiment was prescribed on the seat model. The duration of each simulation was 650 ms, with the first 450 ms used for initial quasi-static equilibrium settling to gravitational acceleration. All simulations were run using LS-DYNA R.9.3 double precision with LS-PrePost 4.8 (64-bit), ANSA v18.1.0 (64-bit) and OriginPro 2019 (64-bit) were used as pre- and post-processing software.

**FIGURE 4:**
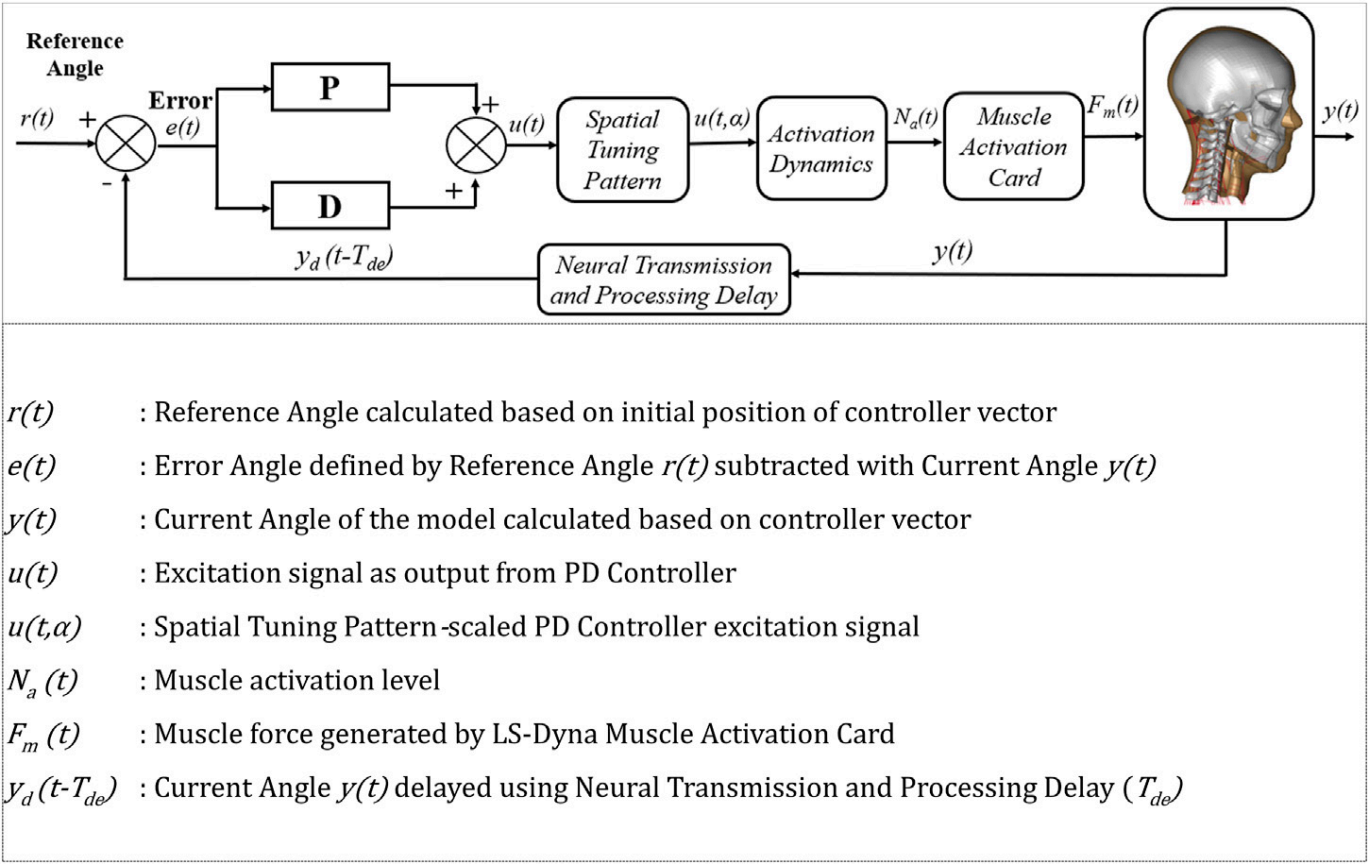
APF PD controler schematic.

The APF controller similar to the VIVA OpenHBM APF Controller (Putra et al., 2020; Putra and Thomson, 2022) was also implemented in VIVA+ Model (Figure 4). The APF Controller was developed to approximate the function of Vestibulocollic reflex (VCR) in humans, which is to maintain head orientation relative to space. In addition to the APF Controller, the optimized muscle CCo level (based on Putra and Thomson, 2022) aimed to maintain an upright model under gravitational loading was added to the VIVA+ model.

It was postulated that the lack of damping contributions of tendon structure and 3D muscle containment in the neck muscle modelling could potentially cause neck buckling when active muscle forces were added to the model. Therefore, a higher damping coefficient than in Putra and Thomson (2022) was implemented in the PDE of LS-DYNA Hill muscle model *MAT_156/*MAT_MUSCLE.

Optimization based parameter identifications were conducted using LS-OPT, a graphical optimization tool (https://www.lsoptsupport.com/) to derive APF controller parameters and update the VIVA OpenHBM controller for the VIVA+ models. The method was adopted from Putra et al. (2020) which derived optimum parameters of: Proportional gain (KPA), Derivative gain (KDA), and the Neural transmission and processing time delay (TNDA) for the PD controller that represents the APF Controller. Parameters of the PDE were also added as an optimization parameter. The full set of parameters for the optimization can be seen in Table 1.

The objective functions for all optimizations were to match the “Sato et al. (2014) 5.8 km/h” volunteer head linear (x- and z-) and rotational displacements as well as cervical spine (C1 to C7) rotational displacements. The initial values of KPA, KDA, and TNDA were based on Putra et al. (2020). The upper and lower range of KPA and KDA for both controllers were set to 0.01 to 100, to make sure a sufficient solution space was available for each parameter (based on the author’s experiences). The range of the TNDA was set from 3.5 ms (Rosengren and Colebatch 2018) to 20 ms (Olafsdottir et al., 2019) and the range of PDE was from 0.01 to 0.05 kN ms/mm^2^ based on preliminary investigation with the VIVA OpenHBM, (Supplementary Table S1).

### 2.4 Selection and evaluation of best model

The head-neck model with the highest CORA scores was selected to be analyzed further. Parametric simulations were conducted with models of different configurations. The purpose of these parametric simulations was to evaluate each feature in the active head-neck models and identify the best agreement with volunteer kinematics data. Six additional simulations were run for each female and male head-neck model (Table 2).

The main purpose of the models is to study neck responses and assess injury risk using simulations at different impact severities. The relation between neck response and injury risk needs further investigation. Neck kinematics should replicate the volunteer responses to generate relevant input to calculate whiplash injury risk. Four head-neck models for each female and male model were selected for evaluation under different loading conditions from those used for calibration. The first evaluation was conducted by comparing the four models to volunteer responses of “Sato et al. (2014) 5.8 km/h” based on head C.G x-acceleration and C1-C7 cervical spine velocities. Those four models were also simulated in higher rear-impact delta velocity based on “Sato et al. (2014) 8.1 km/h”, and “Sato et al. (2014) 10 km/h” However, because the cervical spine information of “Sato et al. (2014) 8.1 km/h”, and “Sato et al. (2014) 10 km/h” were not available, only head C.G x-accelerations were compared. After those evaluations, two of the four models with the highest CORA score were selected and evaluated further. The second evaluation was conducted with the full-body model. Four, full-body simulations for both female and male model based on “Sato et al. (2014) 5.8 km/h” were conducted. CORA evaluations were conducted for relevant injury prediction criteria based on head C.G x-acceleration, T1 C.G x-acceleration and C1-C7 rotational velocities. A female and male configuration with the highest CORA score can then be identified for injury prediction studies such as future accident reconstruction.

Additionally, objective evaluation ratings were conducted using Correlation Analysis (CORAplus) software 4.0.4 with the objective to quantify the similarities of the head-neck model and volunteer kinematics responses (Gehre et al., 2009). Default corridors of CORA (5% inner limit and 50% outer limits) were used.

## 3 Results

### 3.1 *In silico* vs. *in vivo* kinematics

Optimization runs using LS-OPT resulted into sets of APF controller parameters (KPA, KDA, and TNDA) and PDE, one for the female and one for the male VIVA+ models (Table 3).

**TABLE 3:** Active (PSV + PDE + CCo + APF) muscle controller parameter based on optimization.

Model	Parameter			
	Proportional gain/KPA (%contraction/rad)	Derivative gain/KDA (%contraction/rad ms-^1^)	Neural transmission and processing delay/TNDA (ms)	Parallel damping coefficient/PDE (kN.ms/mm^2^)
Active (PSV + PDE + CCo + APF) VIVA+ Female	0.1952	34.093	4.233	0.0303
Active (PSV + PDE + CCo + APF) VIVA + Male	0.01	93.48	19.66	0.0192

**FIGURE 5:**
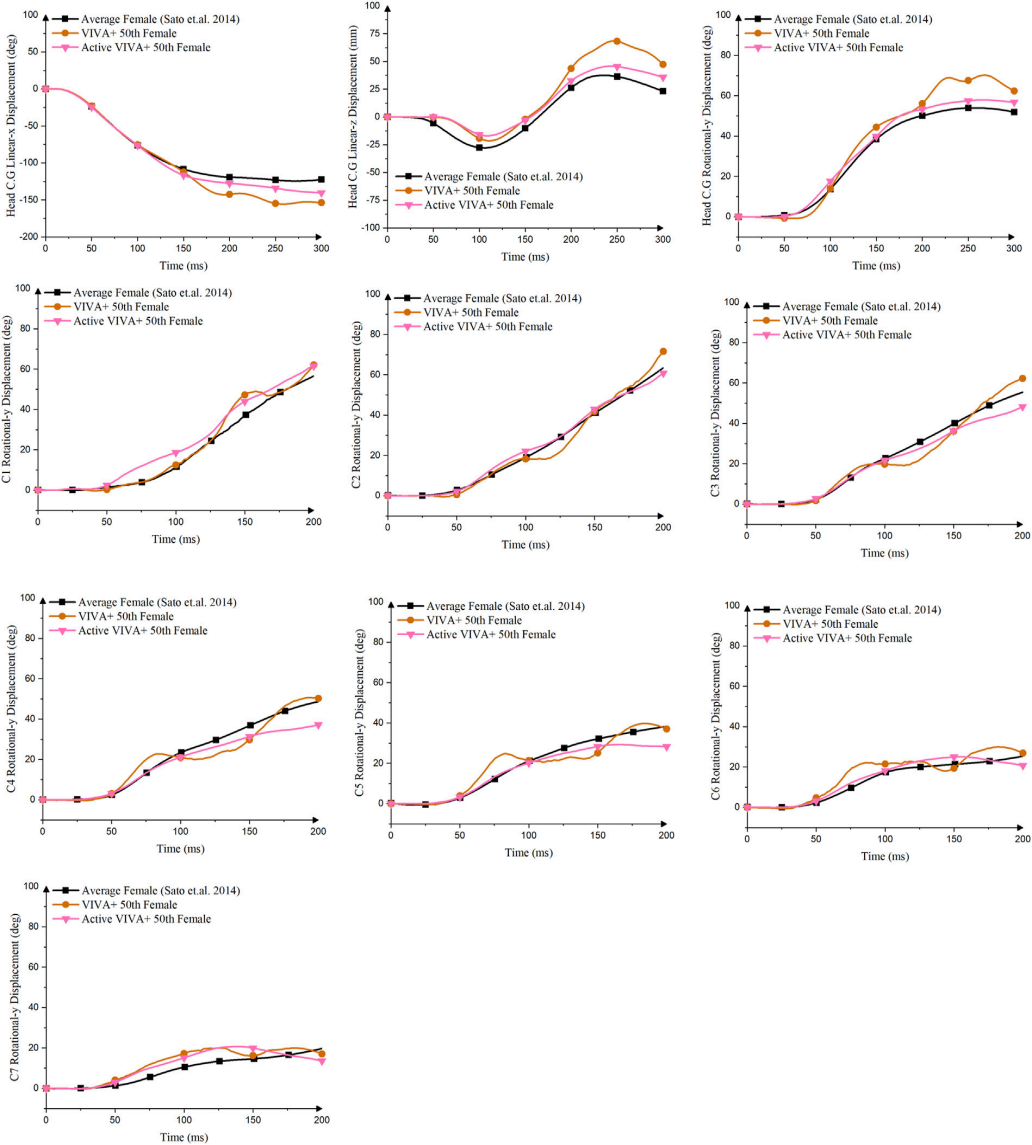
Comparison of Head C.G and Cervical Vertebra C.G linear and rotational displacements between Original VIVA+ Female Model, Active (PSV + PDE + CCo + APF) Female VIVA+ Models and Volunteer Kinematics from Sato et al. (2014) 5.8 km/h.

To objectively compare the different model formulations, Table 4 shows the CORA scores for all displacement data available. For the female and male models, the Active (PSV + PDE + CCo + APF) VIVA+ Female and Active (PSV + PDE + CCo + APF) VIVA+ Male models had the highest total average scores. Noteworthy improvements in terms of agreement with volunteers’ displacements and rotations from Sato et al. (2014) 5.8 km/h were obtained in the Active (PSV + PDE + CCo + APF) Female VIVA+ model compared to the original passive Female VIVA+ model (Figure 5). The VIVA+ model with PDE + CCo + APF controllers also removed the buckling observed in the original model, thus improving the predicted cervical rotations compared to the volunteers. However, the C1 C.G rotates around 4–10° more than the volunteer responses. But, the active model slightly underpredicted the volunteers’ cervical spine rotations from C3 to C7.

**TABLE 4:** CORA Score of Head and Cervical Spine Kinematics of Active (PSV + PDE + CCo + APF) VIVA+ Models compared to “Sato et al. (2014) 5.8 km/h”.

Kinematics	VIVA+ head-neck female model		VIVA+ head-neck male model	
	VIVA+ 50th female	Active (PSV + PDE + CCo + APF) VIVA+ female	VIVA+ 50th male	Active (PSV + PDE + CCo + APF) VIVA+ male
HCG-x	0.832	0.943	0.761	0.864
HCG-z	0.523	0.644	0.836	0.803
HCG-ry	0.816	0.961	0.757	0.813
Average HCG	0.724	0.849	0.785	0.827
C1-ry	0.944	0.880	0.863	0.696
C2-ry	0.972	0.994	0.827	0.833
C3-ry	0.938	0.942	0.873	0.955
C4-ry	0.903	0.883	0.905	0.857
C5-ry	0.877	0.895	0.844	0.872
C6-ry	0.779	0.913	0.670	0.802
C7-ry	0.705	0.707	0.677	0.729
Average Cervical Spine	0.874	0.888	0.808	0.821
Total Average	0.799	0.869*	0.796	0.824**^a^**

^a^Best average score.

The male version of the model exhibited similar characteristics as the female–the optimized controller produced almost identical responses for both head C.G. and cervical spine C.G. kinematics (Supplementary Figure S1) compared to volunteers, better than the passive model. The Active (PSV + PDE + CCo + APF) VIVA+ male model also reduced the cervical vertebral rotations, especially after 150 ms, improving the overall cervical spine rotations compared with volunteers. The original VIVA+ model was better at mimicking the volunteers’ rotational displacements of C1 and C4 than the active (PSV + PDE + CCo + APF) models.

### 3.2 Best model configuration

CORA scores of female and male VIVA+ models with various configurations are presented in Tables 5; Supplementary Table S2. In the female model, the four models with the highest average CORA scores were the models with PDE. The highest average scores were obtained for the PSV + PDE + APF. For the male models the best configuration was the PSV + PDE, followed by the PSV + PDE + APF.

**TABLE 5:** CORA Score of Head and Cervical Spine Kinematics compared to “Sato et al. (2014) 5.8 km/h”.

Kinematics	PSV	PSV + CCo	PSV + PDE	PSV + APF<	PSV + CCo + PDE	PSV + CCo + APF<	PSV + PDE + APF	PSV + CCo + PDE + APF
**HCG-x**	0.832	0.793	0.961	0.868	0.880	0.829	0.994	0.943
**HCG-z**	0.523	0.431	0.810	0.511	0.579	0.428	0.805	0.644
**HCG-ry**	0.816	0.731	0.991	0.820	0.930	0.733	0.989	0.961
**Average HCG**	**0.724**	**0.652**	**0.921**	**0.733**	**0.796**	**0.663**	**0.929**	**0.849**
**C1-ry**	0.944	0.792	0.952	0.925	0.902	0.781	0.970	0.880
**C2-ry**	0.972	0.849	0.937	0.979	0.987	0.861	0.919	0.994
**C3-ry**	0.938	0.877	0.874	0.935	0.981	0.912	0.853	0.942
**C4-ry**	0.903	0.916	0.853	0.863	0.940	0.902	0.818	0.883
**C5-ry**	0.877	0.902	0.905	0.820	0.965	0.832	0.848	0.895
**C6-ry**	0.779	0.799	0.911	0.781	0.855	0.825	0.966	0.913
**C7 -ry**	0.705	0.687	0.692	0.747	0.694	0.752	0.703	0.707
**Average Cervical Spine**	**0.874**	**0.832**	**0.875**	**0.864**	**0.903**	**0.838**	**0.868**	**0.888**
**Total Average**	**0.799**	**0.742**	**0.898**	**0.799**	**0.850**	**0.751**	0.899*	**0.869**

a Best average score.

Four models without PDE (PSV, PSV + CCo, PSV + APF, and PSV + CCo + APF) produced noticeably higher head C.G displacements started at around 100 ms after the impact compared to the models with PDE (PSV + PDE, PSV + CCo + PDE, PSV + PDE + APF, and PSV + CCo + PDE + APF) (Supplementary Figures S2, 3). More pronounced differences were observed in vertebral rotations. Oscillations in the cervical spine occurred in the four models without PDE (PSV, PSV + CCo, PSV + APF, and PSV + CCo + APF) but were removed when PDE was added to the model.

### 3.3 Evaluations outcome of the best model

#### 3.3.1 Head neck model

**FIGURE 6:**
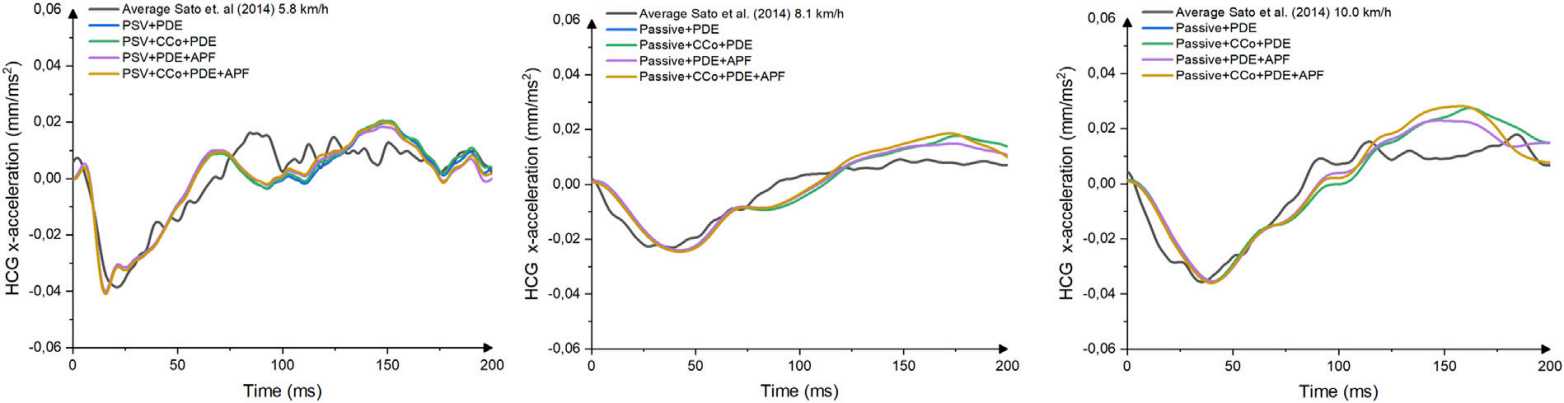
Comparison of Head C.G x-Acceleration between Female Models with Various Complexities and Volunteer Kinematics from Sato et al. (2014) 5.8 km/h, Sato et al. (2014) 8.1 km/h, and Sato et al. (2014) 10 km/h.

**FIGURE 7:**
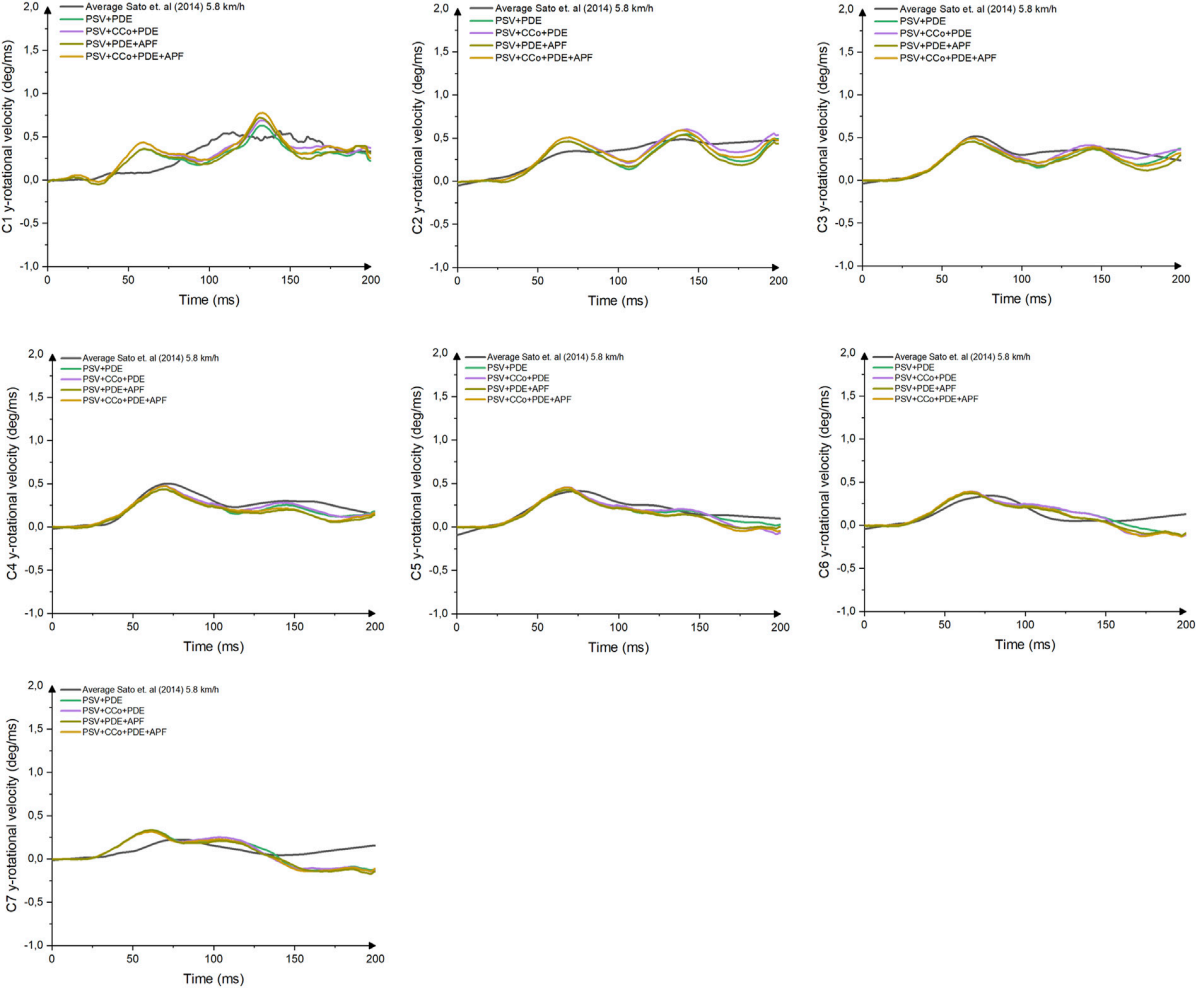
Comparison of Cervical Vertebral Rotational y-Velocity between Female Models with Various Complexities and Volunteer Kinematics.

Comparison of head C.G x-acceleration and cervical spine rotational velocities were conducted to evaluate the performance of VIVA+ models for predicting whiplash injuries criteria (NIC and pressure transient in vertebral canal) (Figures 6, 7 and Supplementary Figures S4,5). The four models with PDE had slightly different responses compared to each other. However, when the head C.G x-acceleration of those models was compared to volunteers’ responses, none of them perfectly matched the volunteers’ acceleration.

A comparison of cervical vertebral rotational velocities (Figure 6) revealed high gradients for velocities in the VIVA+ female upper vertebrae. However, in the lower cervical spine (C4-C7), the VIVA+ rotational velocities response follows the results in Sato et al. (2014) 5.8 km/h. No pronounced differences between the four different models were observed.

The differences between various complexities of the model’s head C.G x-acceleration of the VIVA+ male models (Supplementary Figure S4) were more pronounced after 150 ms of impact. The model with damping and an APF controller was the model that produced the closest response to volunteer head C.G x-accelerations.

All VIVA+ male models generated almost identical cervical spine responses up until 150 ms (Supplementary Figure S5) before they started to deviate from the volunteer reference data. In addition, the PSV + PDE model had almost similar rotational velocities throughout the simulation’s time to the PSV + CCo + PDE and the PSV + PDE + APF models.

CORA score evaluations showed similar results between female and male models when the head C.G x-acceleration and cervical vertebral rotational y-velocities were compared to the volunteer responses (Table 6; Supplementary Table S3). In both models, the model that best replicates the volunteers was the model with PDE and APF controller (PSV + PDE + APF). The CORA scores were slightly different between the female and male model, with the female and male model obtaining CORA score of 0.667 and 0.653, respectively which were lower than the previous CORA score evaluation of the head-neck displacements.

**TABLE 6:** CORA score of female head-neck model injury criteria input.

Kinematics	PSV + PDE	PSV + CCo + PDE	PSV + PDE + APF	PSV + CCo + PDE + APF
HCG x-acceleration (5.8 km/h)	0.70	0.703	0.717	0.726
C1-ry velocity (5.8 km/h)	0.660	0.713	0.648	0.671
C2-ry velocity (5.8 km/h)	0.719	0.788	0.691	0.770
C3-ry velocity (5.8 km/h)	0.804	0.907	0.757	0.852
C4-ry velocity (5.8 km/h)	0.800	0.867	0.713	0.765
C5-ry velocity (5.8 km/h)	0.822	0.780	0.721	0.708
C6-ry velocity (5.8 km/h)	0.637	0.562	0.654	0.60
C7-ry velocity (5.8 km/h)	0.383	0.381	0.398	0.392
Average Cervical Spine (5.8 km/h)	0.689	0.714	0.655	0.680
**Average (5.8 km/h)**	**0.694**	**0.708**	**0.686**	**0.703**
**HCG x-acceleration (8.1 km/h)**	**0.603**	**0.603**	**0.620**	**0.606**
**HCG x-acceleration (10.0 km/h)**	**0.625**	**0.625**	**0.696**	**0.647**
**Total Average (5.8, 8.1, 10.0 km/h)**	**0.641**	**0.645**	**0.667** *	**0.652**

a Best average score.

#### 3.3.2 Full body model

No notable difference in trends between the model with PDE + APF and PDE + CCo + APF was observed in female and male VIVA+ full-body head and neck responses, only a slight offset in magnitudes (Table 7, Supplementary Figures S6, 7). It was observed that the VIVA+ model with PDE + APF controller best matched volunteer kinematics. The models’ head C.G x-accelerations followed the volunteers’ acceleration closely. In comparison, the model T1 C.G x-accelerations lagged behind the volunteers’. Comparison of cervical spine rotational velocities showed that the model followed the reference velocity profiles well but did not perfectly match their peaks.

**TABLE 7:** CORA score of female and male full-body model for whiplash injury assessment simulation.

Kinematics	VIVA+ female full-body model		VIVA+ male full-body model	
	PSV + PDE +APF	PSV + CCo + PDE + APF	PSV + PDE +APF	PSV + CCo + PDE + APF
HCG x-acceleration	0.751	0.662	0.727	0.763
C1-ry velocity	0.769	0.764	0.794	0.784
C2-ry velocity	0.632	0.631	0.772	0.805
C3-ry velocity	0.764	0.736	0.661	0.695
C4-ry velocity	0.730	0.685	0.670	0.670
C5-ry velocity	0.689	0.610	0.743	0.726
C6-ry velocity	0.675	0.684	0.750	0.807
C7-ry velocity	0.456	0.477	0.644	0.671
**Average Cervical Spine**	**0.674**	**0.655**	**0.719**	**0.737**
T1 x acceleration	0.519	0.498	0.567	0.557
**Total Average**	**0.648** *	**0.605**	**0.671**	**0.686** *

a Best average score.

Objective CORA ratings (Table 7) showed that female and male VIVA+ full-body models had different scores when the models were compared to Sato et al. (2014) 5.8 km/h volunteers’ data. The best model based on the CORA scores was achieved by the PSV + PDE + APF model for the female VIVA+ model. However, for the male VIVA+ model, the best agreement with volunteers’ kinematics was obtained by the PSV + CCo + PDE + APF model. Although, the difference with the PSV + PDE + APF model was not as pronounced as in the female VIVA+ models. The male models had higher CORA score than the female models.

## 4 Discussion

### 4.1 Improving agreement between *in silico* and *in vivo* kinematics

In previous studies the intervertebral kinematics in the lower cervical spine (C4–C6) of VIVA OpenHBM did not agree with volunteer responses (Putra et al., 2019, 2020; Putra and Thomson, 2022), and APF controller caused oscillations and buckling. It was postulated that the buckling and instabilities were caused by the limitations of the current HIll muscle model implementation in LS-DYNA, which neglected serial elastic and damping elements to represent the tendon structures (Kleinbach et al., 2017). In addition, the muscle model in VIVA OpenHBM was modeled as 1D Truss elements and the model lacked damping and stiffness effects from 3D muscle containment of the vertebrae. Another study found that the 3D muscle models could stiffen the neck model response compared to 1D muscle (Hedenstierna and Halldin, 2008). The damping value of 0.004 kN ms/mm^2^ initially used to define PDE for the VIVA OpenHBM was based on Östh et al. (2012). It was derived from simulations to achieve reasonable agreement to Hayes and Hatze (1977) experimental studies. However, this value may not be suitable for the present model as the study conducted by Östh et al. (2012) was based on simulations of a human arm. The new results found the suitable range was 5–10 times higher than previously used (0.02 kN ms/mm^2^ to 0.04 kN ms/mm^2^). With the new value of the PDE, the oscillations and buckling on the cervical spine rotational displacements were minimized or even removed.

Tuning of the APF controller by including the damping values produced much better performance of the models. It was found that a lower PDE damping coefficient was needed for the male model compared to the female model. There are two main reasons why the VIVA+ male model needed less damping coefficient than the female model. The first reason was that no oscillations occurred in the passive male VIVA+ model. The second reason could be the male’s neck larger anatomical geometry provides more damping properties than the female’s. With higher passive dampening effects in the neck, the male VIVA+ model needed less damping explicitly modelled in the muscle elements than the female model as observed in the lower of PDE coefficient.

Based on the head-neck kinematics and CORA score evaluations, the active models for female and male models produced better head-neck kinematics agreement with the volunteers than the original passive VIVA+ models. Furthermore, the active models have more representative cervical spine rotations as measured by the Sato et al. (2014) 5.8 km/h volunteer test without any oscillations or buckling. These results are very important if the model from the current study is used for the whiplash injury prediction. Apart from a good prediction of head C.G and T1 C.G horizontal acceleration, the correct prediction of the cervical vertebral sagittal rotations are necessary for analyzing proposed injury mechanism hypotheses based on cervical spine motion, such as a transient pressure at the spinal root ganglion (Yao et al., 2016). Compared to previous publications that implemented APF controllers for low-speed rear-impact applications (Putra et al., 2019, 2020; Putra and Thomson, 2022), the models from the current study produced much better agreements with volunteer kinematics and show the importance of PDE in the model with APF controllers.

Hessel et al. (2017) showed an almost instant muscle force build up as the muscle is exposed to sudden rapid involuntary eccentric elongation. This phenomenon was shown to be independent of the muscle EMG activity. The neck muscle would probably experience a similar type of involuntary eccentric elongation in the typical rear-end impact that could lead to whiplash injuries. The inclusion of PDE in the present study could potentially be a way to substitute the phenomenon presented by Hessel et al. (2017). However, the inclusion of PDE may have limitations since it is more velocity-dependent than the force from eccentric muscle reaction.

### 4.2 Role of added features in the active model for improved kinematics

Further analyses were conducted to analyze the kinematics performance of models with different complexities. Both Active VIVA+ 50th Female and Male were developed by adding muscle CCo level, PDE, and APF controller to the passive models. However, it was not clear how each additional feature contributed to the model’s head-kinematics agreement with volunteers’ kinematics.

Eight simulations were generated to investigate the relevance of each model feature and their combinations. These simulations again revealed the role of the PDE since it was observed that any model that included PDE improved head and neck kinematics compared to the original passive model. No optimization run was conducted to re-optimize the damping coefficient or the APF Controller for these additional conditions. Further optimizations would be expected to improve the model performance, but the marginal improvements expected were not considered to justify the considerable computational resources needed.

Muscle CCo has been known to maintain the neck’s spinal stability by simultaneously activating agonistic and antagonistic muscles (Le et al., 2017). In the present study, the muscle CCo level was adapted from the previous work using the VIVA OpenHBM model (Putra and Thomson, 2022) to ensure that the model stays upright during gravitational loading. The CCo level based on Putra and Thomson (2022) was used because no published experimental data was found that can be directly applied to the present study’s model. The CCo ratios in neck muscle published by Choi (2003) could not be directly adapted for the activation level for Hill’s muscle model used in the current model. It was observed when CCo was included in the passive model without PDE, objective rating scores were below the original passive model. In parallel, when all models with PDE were compared, lower average CORA scores were observed when the CCo was included. This result implies that the CCo level based on another model was not appropriate to be used for the present study model. The difference in model behaviour after gravity settling could be the main reason. Thus, the present models only need CCo level during gravity settling to maintain a stable initial position before the pulse is applied.

Four evaluations of head acceleration and cervical kinematics at higher impact severities simulations (8.1 and 10 km/h) were conducted. Head C.G x-acceleration and cervical spine rotational velocities are the key kinematic inputs. The primary input for Neck Injury Criteria (NIC) (Boström et al., 2000) is head and T1 C.G x-acceleration. For the indirect tissue-based injury assessment, such as the analysis of pressure gradients in the spinal canal (Svensson et al., 1998; Yao et al., 2016), the cervical vertebral rotational velocities are input into the calculation. Therefore, it is crucial to have the head C.G x-acceleration and cervical vertebral rotational velocities as close as possible to volunteer kinematics responses.

The four best models were compared with volunteer data at higher delta velocities (8.1 and 10.0 km/h). Only head kinematics between the volunteer and models were compared since the cervical spine vertebral rotational velocities were unavailable in those datasets. Despite this limitation, comparing model performance at higher delta velocity is vital to ensure that the developed model also had a good head kinematics agreement. Nevertheless, the current model developed in this study had been validated for the cervical spine rotations despite a lower delta velocity. At a delta velocity of 5.8 km/h, it was known that the model had replicated the volunteer head and neck kinematics reasonably well. In higher delta velocities, the model was again proved to have a good head kinematics agreement with volunteer data. Although there is no guarantee, the neck rotations will also have a good agreement at that higher speed. In addition, evaluating models at higher delta velocities is vital because whiplash injury cases have been reported to occur at delta velocities up to 25 km/h (Kullgren et al., 2003). The volunteer datasets of Sato et al. (2014) with a delta velocity of 10 km/h are one of the few published low-speed rear-end impact volunteer tests with a relatively high delta velocity and reproducible test setup.

Full-body model evaluations were conducted as the final evaluation to select the models, including the metrics to be used for whiplash injury simulation and assessment. Due to the lack of validated FE seat models available for the other published volunteers’ rear-impact tests, the data of Sato et al. (2014) with a delta velocity of 5.8 km/h was again used to evaluate the models. Simple test setup and complete datasets, which also included measured cervical spine rotational y-displacements, are the main benefits of these datasets. The full-body model can be further validated with other volunteer datasets to increase the user’s confidence in the model.

Based on CORA evaluation in the full-body model simulations, the best model for conducting injury prediction studies was the model with PDE and an APF controller for the female model. In parallel, for the male VIVA+ model, the best model was one with CCo, PDE, and APF controller. However, since the CORA score differences between the male VIVA+ models with PDE + APF controller and with CCo + PDE + APF controller were very small (0.015), it is strategic to use the VIVA+ male model with PDE + APF controller. Using a simpler male model is beneficial from the modeling point of view. In addition, using a model with similar features for both female and male VIVA+ models will allow for more direct comparison of results evaluating the influence of sex to whiplash injury risk.

In summary, the model with APF controller and PDE developed in the present study was suitable for whiplash injuries simulations in low-speed rear-end impacts. The model replicated volunteers’ essential kinematics, usually used as input for global kinematics-based injury criteria and indirect local injury prediction. The developed model also showed relatively good agreement with volunteers kinematics at higher impact severities. Therefore, FE HBMs representing the average female and male with active reflexive neck muscles are now available as open access and can be used in future whiplash injury prediction studies.

## Data Availability

The original contributions presented in the study are included in the article/Supplementary Material, further inquiries can be directed to the corresponding author.

## References

[B1] Blouin J. S. Inglis J. T. Siegmund G. P. (2006). Auditory startle alters the response of human subjects exposed to a single whiplash-like perturbation. Spine 31, 146–154. 10.1097/01.brs.0000195157.75056.df 16418632

[B2] Boström O. Fredriksson R. Håland Y. Jakobsson L. Krafft M. Lövsund P. (2000). Comparison of car seats in low speed rear-end impacts using the BioRID dummy and the new neck injury criterion (NIC). Accid. Analysis Prev. 32, 321–328. 10.1016/S0001-4575(99)00105-0 10688488

[B3] Brault J. R. Siegmund G. P. Wheeler J. B. (2000). Cervical muscle response during whiplash: Evidence of a lengthening muscle contraction. Clin. Biomech. (Bristol, Avon. 15, 426–435. 10.1016/S0268-0033(99)00097-2 10771121

[B4] Carlsson A. Linder A. Davidsson J. Hell W. Schick S. Svensson M. (2011). Dynamic kinematic responses of female volunteers in rear impacts and comparison to previous male volunteer tests. Traffic Inj. Prev. 12, 347–357. 10.1080/15389588.2011.585408 21823943

[B5] Cassidy J. D. Linda J. C. Coté P. Lemstra M. Berglund A. Nygren Å. (2000). Effect of eliminating compensation for pain and suffering on the outcome of insurance claims for whiplash injury. N. Engl. J. Med. 342, 1179–1186. 10.1056/NEJM200004203421606 10770984

[B6] Choi H. (2003). Quantitative assessment of co-contraction in cervical musculature. Med. Eng. Phys. 25, 133–140. 10.1016/S1350-4533(02)00151-0 12538067

[B7] Dehner C. Schick S. Kraus M. Scola A. Hell W. Kramer M. (2013). Muscle activity influence on the kinematics of the cervical spine in frontal tests. Traffic Inj. Prev. 14, 607–613. 10.1080/15389588.2012.734937 23859764

[B8] Forman J. Poplin G. S. Shaw C. G. McMurry T. L. Schmidt K. Ash J. (2019). Automobile injury trends in the contemporary fleet: Belted occupants in frontal collisions. Traffic Inj. Prev. 20, 607–612. 10.1080/15389588.2019.1630825 31283362

[B9] Gehre C. Gades H. Wernicke P. (2009). Objective rating of signals using test and simulation responses. Int. Tech. Conf. Enhanc. Saf. Veh., 09-0407.

[B10] Gisolf-Berecki J. Collie A. McClure R. (2013). Work disability after road traffic injury in a mixed population with and without hospitalisation. Accid. Analysis Prev. 51, 129–134. 10.1016/j.aap.2012.11.010 23220006

[B11] Grauer J. N. Panjabi M. M. Cholewicki J. Nibu K. Dvorak J. (1997). Whiplash produces an S-shaped curvature of the neck with hyperextension at lower levels. Spine, 22 (21), 2489–2494. 10.1097/00007632-199711010-00005 9383854

[B12] Günther M. Schmitt S. Wank V. (2007). High-frequency oscillations as a consequence of neglected serial damping in Hill-type muscle models. Biol. Cybern. 97, 63–79. 10.1007/s00422-007-0160-6 17598125

[B13] Hayes K. C. Hatze H. (1977). Passive visco-elastic properties of the structures spanning the human elbow joint. Eur. J. Appl. Physiol. Occup. Physiol. 37, 265–274. 10.1007/BF00430956 598364

[B14] Hedenstierna S. Halldin P. (2008). How does a three-dimensional continuum muscle model affect the kinematics and muscle strains of a finite element neck model compared to a discrete muscle model in rear-end, frontal, and lateral impacts. Spine 33, E236–E245. 10.1097/BRS.0b013e31816b8812 18404093

[B15] Hessel A. L. Lindstedt S. L. Nishikawa K. C. (2017). Physiological mechanisms of eccentric contraction and its applications: A role for the giant titin protein. Front. Physiol. 8, 70. 10.3389/fphys.2017.00070 28232805 PMC5299520

[B16] John J. Klug C. Kranjec M. Svenning E. Iraeus J. (2022a). Hello, world! VIVA+: A human body model lineup to evaluate sex-differences in crash protection. Front. Bioeng. Biotechnol 10, 918904. 10.3389/fbioe.2022.918904 35928956 PMC9343945

[B17] John J. Putra I. P. A. Iraeus J. (2022b). Finite element human body models to study sex-differences in whiplash: Validation of VIVA+ passive responses in rear-impact,” in IRCOBI Conference. IRC-22-36, Porto, Portugal

[B18] Kleinbach C. Martynenko O. Promies J. Haeufle D. F. B. Fehr J. Schmitt S. (2017). Implementation and validation of the extended Hill-type muscle model with robust routing capabilities in LS-DYNA for active human body models. Biomed. Eng. Online 16, 109–128. 10.1186/s12938-017-0399-7 28865494 PMC5581498

[B19] Kullgren A. Krafft M. Tingvall C. Lie A. (2003). “Combining crash recorder and paired comparison technique: Injury risk functions in frontal and rear impacts with special reference to neck injuries” in Proceeding 18th International Research Council on the Biomechanics of Injury Conference, IRCOBI, Gothenburg, Sweden, 51–62.

[B20] Kullgren A. Stigson H. Krafft M. (2013). Development of whiplash associated disorders for male and female car occupants in cars launched since the 80s in different impact directions. Traffic Inj. Prev. 46, 51.

[B21] Le P. Best T. M. Khan S. N. Mendel E. Marras W. S. (2017). A review of methods to assess coactivation in the spine. J. Electromyogr. Kinesiol. 32, 51–60. 10.1016/j.jelekin.2016.12.004 28039769

[B22] Mang D. W. H. Siegmund G. P. Brown H. J. Goonetilleke S. C. Blouin J. S. (2015). Loud preimpact tones reduce the cervical multifidus muscle response during rear-end collisions: A potential method for reducing whiplash injuries. Spine J. 15, 153–161. 10.1016/j.spinee.2014.08.002 25110275

[B23] Mörl F. Siebert T. Schmitt S. Blickhan R. Günther M. (2012). Electro-mechanical delay in hill-type muscle models. J. Mech. Med. Biol. 12, 1250085. 10.1142/S0219519412500856

[B24] Ólafsdóttir J. M. Östh J. Brolin K. (2019). Modelling reflex recruitment of neck muscles in a finite element human body model for simulating omnidirectional head kinematics. Conf. Proc. Int. Res. Counc. Biomech. Inj. IRCOBI, 308.

[B25] Ono K. Ejima S. Suzuki Y. Kaneoka K. Fukushima M. Ujihashi S. (2006). Prediction of neck injury risk based on the analysis of localized cervical vertebral motion of human volunteers during low-speed rear impacts. IRCOBI Conf. Proc., 103.

[B26] Ono K. Kaneoka K. Wittek A. Kajzer J. (1997). Cervical injury mechanism based on the analysis of human cervical vertebral motion and head-neck-torso kinematics during low speed rear impacts SAE Technical Paper, 973340. 10.4271/973340

[B27] Östh J. Brolin K. Happee R. (2012). Active muscle response using feedback control of a finite element human arm model. Comput. Methods Biomech. Biomed. Engin. 15, 347–361. 10.1080/10255842.2010.535523 21294008

[B28] Östh J. Mendoza-Vazquez M. Linder A. Svensson M. Y. Brolin K. (2017a). “The VIVA OpenHBM finite element 50th percentile female occupant model: Whole body model development and kinematic validation,” in IRCOBI Conference.

[B29] Östh J. Mendoza-Vazquez M. Sato F. Svensson M. Y. Linder A. Brolin K. (2017b). A female head–neck model for rear impact simulations. J. Biomech. 51, 49–56. 10.1016/j.jbiomech.2016.11.066 27988036

[B30] Putra I. P. A. Thomson R. (2022). Analysis of control strategies for VIVA OpenHBM with active reflexive neck muscles. Biomech. Model. Mechanobiol. 10.1007/s10237-022-01616-y PMC9700582 35927540

[B31] Putra I. P. A. Iraeus J. Sato F. Svensson M. Y. Linder A. Thomson R. (2020). Optimization of female head–neck model with active reflexive cervical muscles in low severity rear impact collisions. Ann. Biomed. Eng. 1–14, 115–128. 10.1007/s10439-020-02512-1 PMC7773618 32333133

[B32] Putra I. P. A. Iraeus J. Thomson R. Svensson M. Y. Linder A. Sato F. (2019). Comparison of control strategies for the cervical muscles of an average female head-neck finite element model. Traffic Inj. Prev. 20, S116–S122. 10.1080/15389588.2019.1670818 31617760

[B33] Quinlan K. P. Annest J. L. Myers B. Ryan G. Hill H. (2004). Neck strains and sprains among motor vehicle occupants - United States, 2000. Accid. Analysis Prev. 36, 21–27. 10.1016/S0001-4575(02)00110-0 14572823

[B34] Rosengren S. M. Colebatch J. G. (2018). The contributions of vestibular evoked myogenic potentials and acoustic vestibular stimulation to our understanding of the vestibular system. Front. Neurol. 9, 481. 10.3389/fneur.2018.00481 30013504 PMC6037197

[B35] Sato F. Nakajima T. Ono K. Svensson M. (2014). Dynamic cervical vertebral motion of female and male volunteers and analysis of its interaction with head/neck/torso behavior during low-speed rear. Ircobi.Org, 227. Available at: http://www.ircobi.org/wordpress/downloads/irc14/pdf_files/31.pdf.

[B36] Siegmund G. P. Sanderson D. J. Myers B. S. Inglis J. T. (2003). Awareness affects the response of human subjects exposed to a single whiplash-like perturbation. Spine 28, 671–679. 10.1097/01.BRS.0000051911.45505.D3 12671354

[B37] Siegmund G. P. (2011). What occupant kinematics and neuromuscular responses tell us about whiplash injury. Spine 36, S175–S179. 10.1097/BRS.0b013e3182387d71 22020610

[B38] Siegmund G. P. Winkelstein B. A. Ivancic P. C. Svensson M. Y. Vasavada A. (2009). The anatomy and biomechanics of acute and chronic whiplash injury. Traffic Inj. Prev. 10, 101–112. 10.1080/15389580802593269 19333822

[B39] Styrke J. Stålnacke B. M. Bylund P. O. Sojka P. Björnstig U. (2012). A 10-year incidence of acute whiplash injuries after road traffic crashes in a defined population in Northern Sweden. PM&R 4, 739–747. 10.1016/j.pmrj.2012.05.010 22819305

[B40] Svensson M. Y. Aldman B. Boström O. Davidsson J. Hansson H. A. Lövsund P. (1998). Nerve cell damages in whiplash injuries. Animal experimental studies. Orthopade 27, 820–826. 10.1007/pl00003469 9894236

[B41] Svensson M. Y. Aldman B. Hansson H. a. Lövsund P. Seeman T. Suneson a. (1993). “Pressure effects in the spinal canal during whiplash extension motion: A possible cause of injury to the cervical spinal ganglia,” in Int. IRCOBI Conf. Biomech. Impacts, 189.

[B42] Wittek A. Kajzer J. Haug E. (2000). Hill-type muscle model for analysis of mechanical effect of muscle tension on the human body response in a car collision using an explicit finite element code. JSME International Journal Series A Solid Mechanics and Material Engineering 43 (1), 8–18. 10.1299/jsmea.43.8

[B43] Yao H. D. Svensson M. Y. Nilsson H. (2016). Transient pressure changes in the vertebral canal during whiplash motion - a hydrodynamic modeling approach. J. Biomech. 49, 416–422. 10.1016/j.jbiomech.2016.01.005 26827171

[B44] Yoganandan N. Pintar F. A. Stemper B. D. Schlick M. B. Philippens M. Wismans J. (2000). Biomechanics of human occupants in simulated rear crashes: Documentation of neck injuries and comparison of injury criteria. Stapp Car Crash J. 44, 189–204. 10.4271/2000-01-SC14 17458727

