# Supplementary material for: Finite element human body models with active reflexive muscles suitable for sex based whiplash injury prediction
Source: Front Bioeng Biotechnol. 2022 Sep 29;10:968939. doi: 10.3389/fbioe.2022.968939 (PMC9557094; doi:10.3389/fbioe.2022.968939)
Supplement: Supplementary file 1 [file DataSheet1.docx]

Supplementary Material

| **Supplementary Table 1. CORA Score of Head and Cervical Spine Kinematics of APF Controller with Different damping Level** | | | | | | |
| --- | --- | --- | --- | --- | --- | --- |
| **Kinematics** | **APF+Co** | **APF+Co 0.01 Damping** | **APF+Co 0.02 Damping** | **APF+Co 0.03 Damping** | **APF+Co 0.04 Damping** | **APF+Co 0.05 Damping** |
| HCG-x | 0.864 | 0.917 | 0.933 | 0.918 | 0.887 | 0.853 |
| HCG-z | 0.465 | 0.551 | 0.558 | 0.547 | 0.535 | 0.517 |
| HCG-ry | 0.780 | 0.843 | 0.859 | 0.834 | 0.798 | 0.757 |
| **Average HCG** | **0.703** | **0.770** | **0.783** | **0.766** | **0.740** | **0.709** |
| C1-ry | 0.750 | 0.781 | 0.836 | 0.879 | 0.895 | 0.900 |
| C2-ry | 0.865 | 0.922 | 0.966 | 0.977 | 0.953 | 0.926 |
| C3-ry | 0.914 | 0.979 | 0.993 | 0.962 | 0.915 | 0.878 |
| C4-ry | 0.966 | 0.944 | 0.906 | 0.872 | 0.834 | 0.808 |
| C5-ry | 0.824 | 0.814 | 0.795 | 0.781 | 0.759 | 0.747 |
| C6-ry | 0.794 | 0.819 | 0.826 | 0.842 | 0.843 | 0.848 |
| C7 -ry | 0.774 | 0.724 | 0.707 | 0.695 | 0.702 | 0.709 |
| **Average Cervical Spine** | **0.841** | **0.855** | **0.861** | **0.858** | **0.843** | **0.831** |
| **Total Average** | **0.772** | **0.812** | **0.822^*^** | **0.812** | **0.791** | **0.767** |

*** Best average score**

| **Supplementary Table 2. CORA Score of Male Head-Neck Model Displacements** | | | | | | | | |
| --- | --- | --- | --- | --- | --- | --- | --- | --- |
| **Kinematics** | **PSV** | **PSV+ CCo** | **PSV+**  **PDE** | **PSV+**  **APF** | **PSV+**  **CCo+**  **PDE** | **PSV+**  **CCo+**  **APF** | **PSV+**  **PDE+**  **APF** | **PSV+**  **CCo+**  **PDE+ APF** |
| **HCG-x** | 0.761 | 0.790 | 0.807 | 0.791 | 0.817 | 0.812 | 0.850 | 0.864 |
| **HCG-z** | 0.836 | 0.656 | 0.794 | 0.771 | 0.858 | 0.737 | 0.718 | 0.803 |
| **HCG-ry** | 0.757 | 0.582 | 0.839 | 0.759 | 0.786 | 0.680 | 0.874 | 0.813 |
| **Average HCG** | **0.785** | **0.676** | **0.813** | **0.774** | **0.820** | **0.743** | **0.814** | **0.827** |
| **C1-ry** | 0.863 | 0.696 | 0.915 | 0.786 | 0.732 | 0.636 | 0.838 | 0.696 |
| **C2-ry** | 0.827 | 0.704 | 0.905 | 0.895 | 0.781 | 0.742 | 0.972 | 0.833 |
| **C3-ry** | 0.873 | 0.773 | 0.957 | 0.924 | 0.895 | 0.848 | 0.912 | 0.955 |
| **C4-ry** | 0.905 | 0.881 | 0.911 | 0.761 | 0.947 | 0.791 | 0.766 | 0.857 |
| **C5-ry** | 0.844 | 0.839 | 0.922 | 0.786 | 0.902 | 0.799 | 0.842 | 0.872 |
| **C6-ry** | 0.670 | 0.694 | 0.738 | 0.770 | 0.725 | 0.757 | 0.830 | 0.802 |
| **C7 -ry** | 0.677 | 0.728 | 0.699 | 0.679 | 0.719 | 0.722 | 0.698 | 0.729 |
| **Average Cervical Spine** | **0.808** | **0.759** | **0.864** | **0.800** | **0.814** | **0.756** | **0.837** | 0.821 |
| **Total Average** | **0.796** | **0.718** | **0.839*** | **0.787** | **0.817** | **0.749** | **0.825** | **0.824** |

*** Best average score**

| **Supplementary Table 3. CORA Score of Male Head-Neck Model with Various Complexities and Volunteer Datasets** | | | | |
| --- | --- | --- | --- | --- |
| **Kinematics** | **PSV+PDE** | **PSV+CCo+ PDE** | **PSV+PDE+ APF** | **PSV+CCo+**  **PDE+APF** |
| HCG x-acceleration (5.8 km/h) | 0.70 | 0.703 | 0.717 | 0.726 |
| C1-ry velocity (5.8 km/h) | 0.716 | 0.656 | 0.686 | 0.656 |
| C2-ry velocity (5.8 km/h) | 0.860 | 0.846 | 0.920 | 0.899 |
| C3-ry velocity (5.8 km/h) | 0.637 | 0.632 | 0.675 | 0.709 |
| C4-ry velocity (5.8 km/h) | 0.673 | 0.733 | 0.576 | 0.623 |
| C5-ry velocity (5.8 km/h) | 0.751 | 0.751 | 0.639 | 0.675 |
| C6-ry velocity (5.8 km/h) | 0.577 | 0.538 | 0.635 | 0.586 |
| C7-ry velocity (5.8 km/h) | 0.549 | 0.575 | 0.540 | 0.558 |
| Average Cervical Spine (5.8 km/h) | 0.680 | 0.676 | 0.667 | 0.672 |
| **Average (5.8 km/h)** | **0.679** | **0.672** | **0.679** | **0.702** |
| **HCG x-acceleration (8.1 km/h)** | **0.681** | **0.620** | **0.781** | **0.713** |
| **HCG x-acceleration (10.0km/h)** | **0.490** | **0.460** | **0.498** | **0.473** |
| **Total Average**  **(5.8 km/h, 8.1 km/h, 10.0 km/h)** | **0.617** | **0.584** | **0.653*** | **0.629** |

*** Best average score**


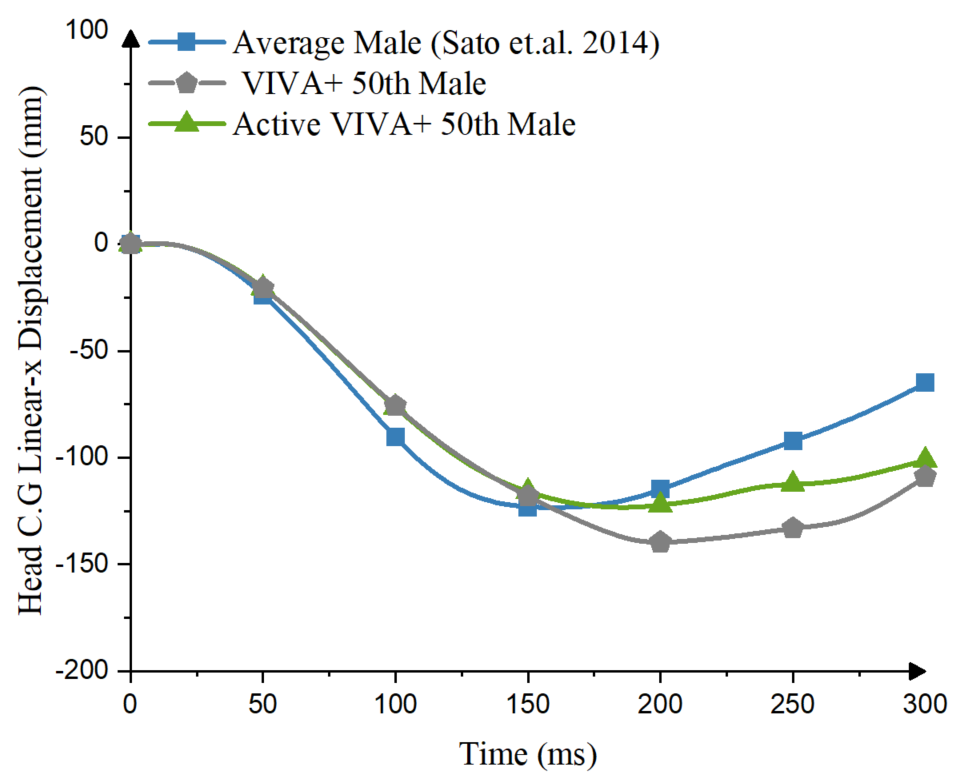

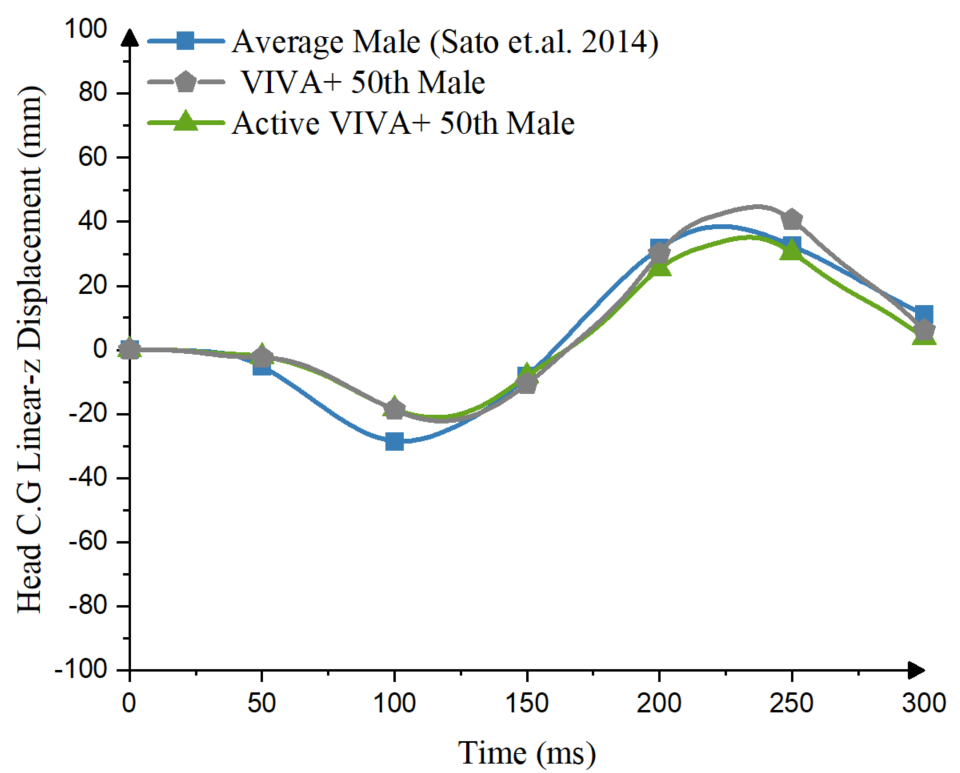

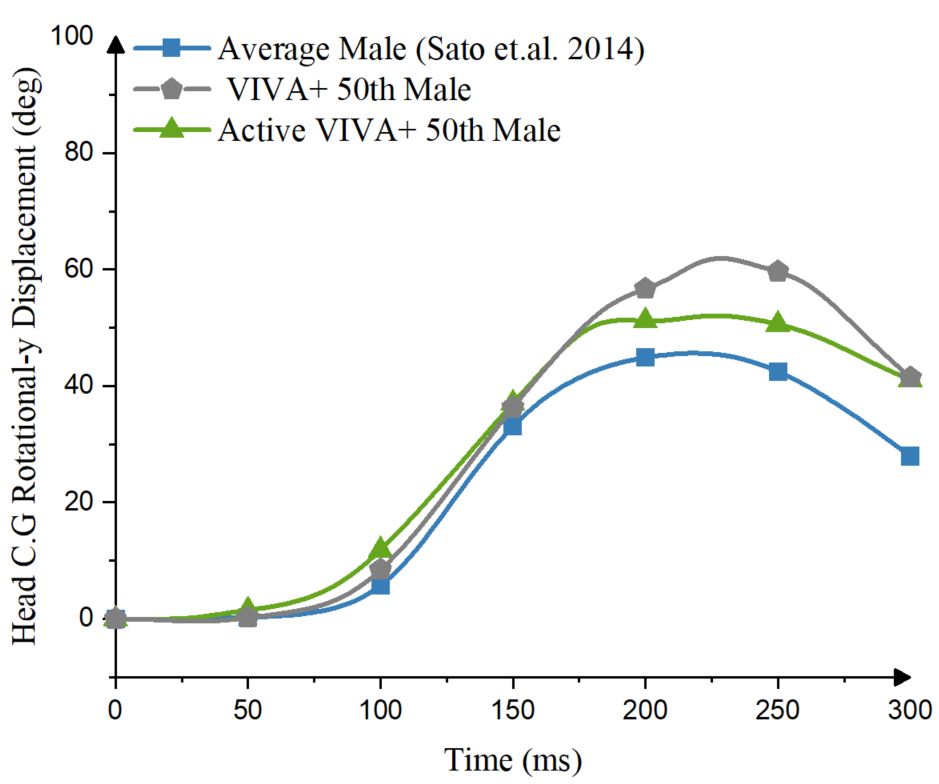


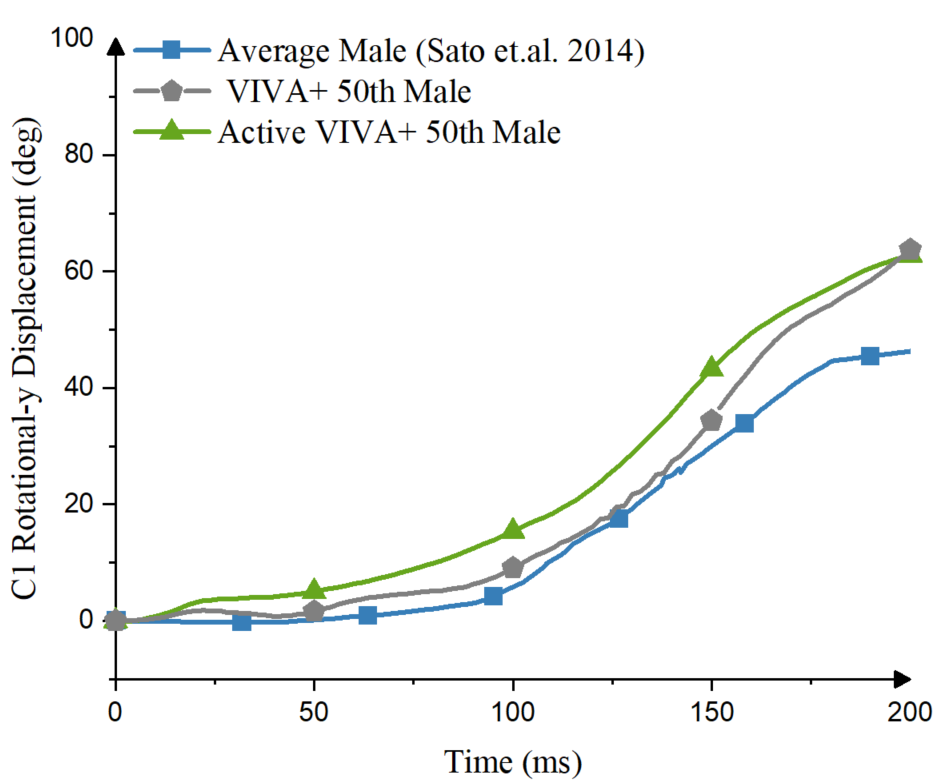

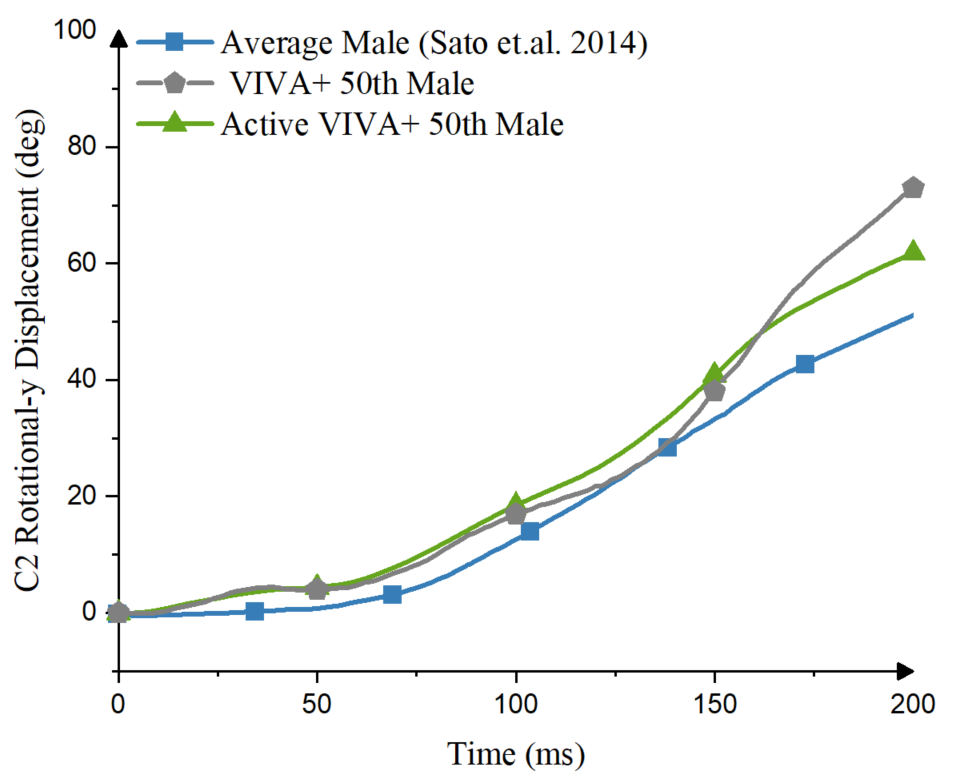

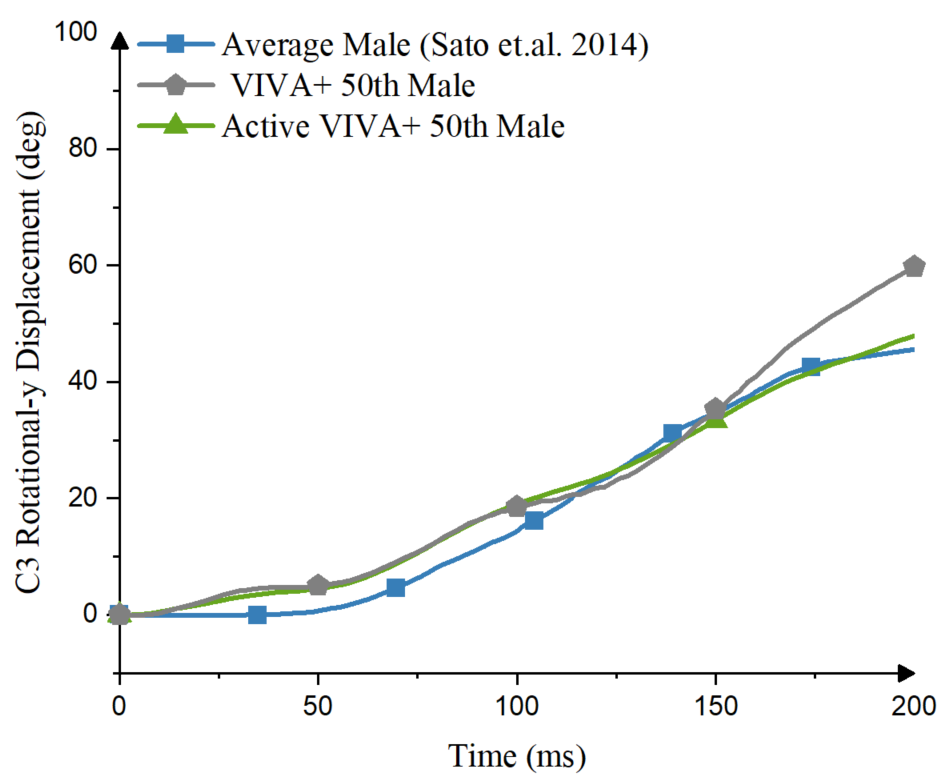


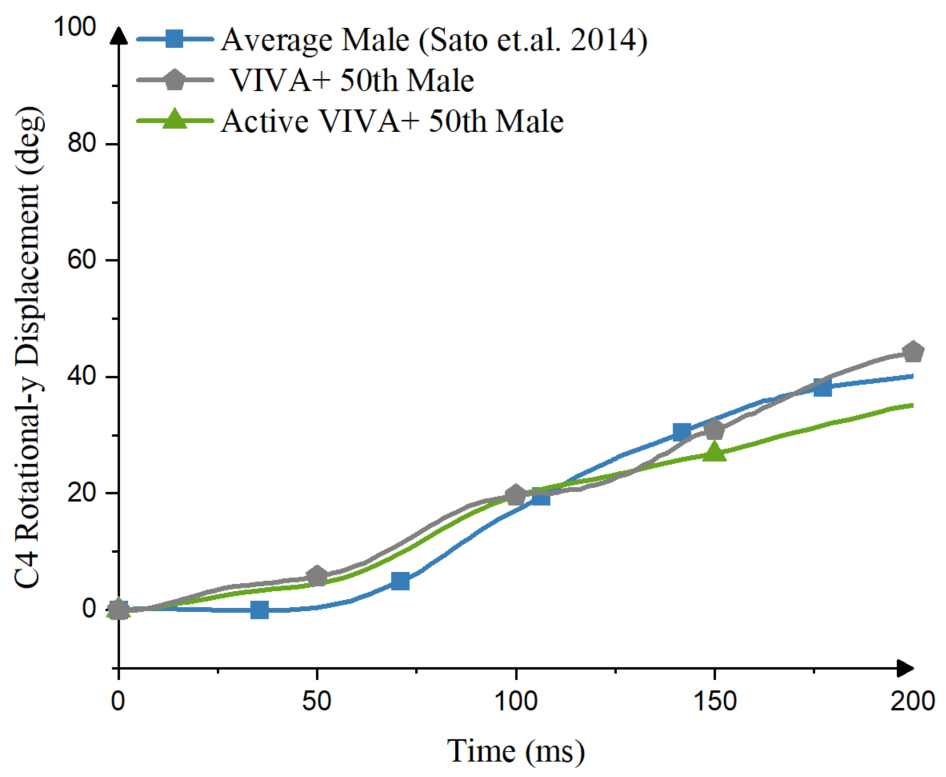

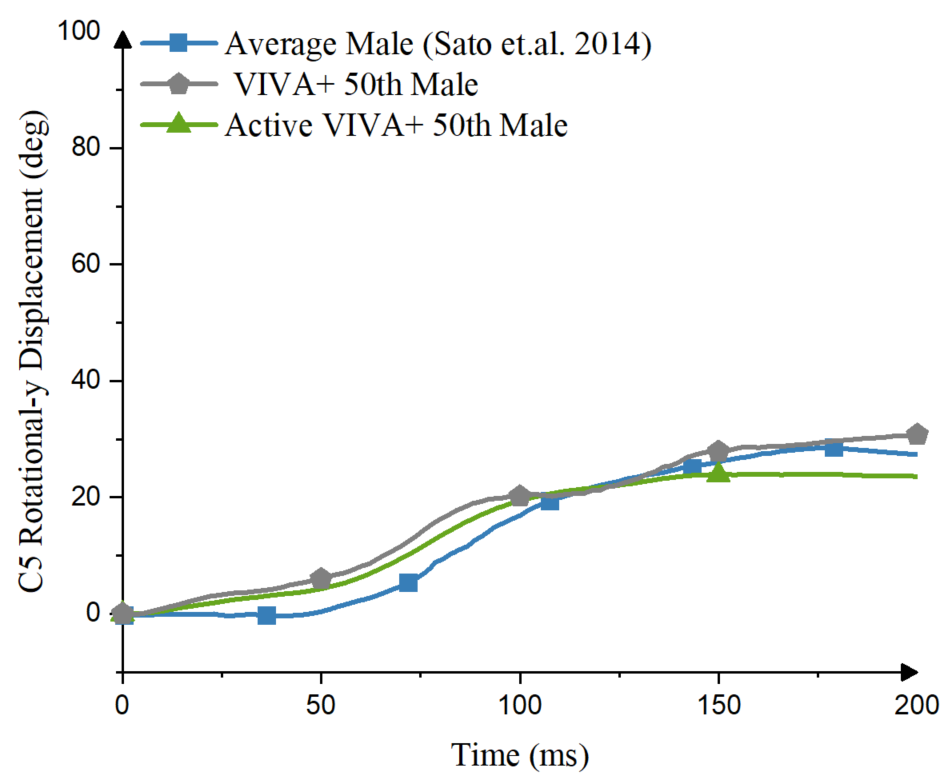

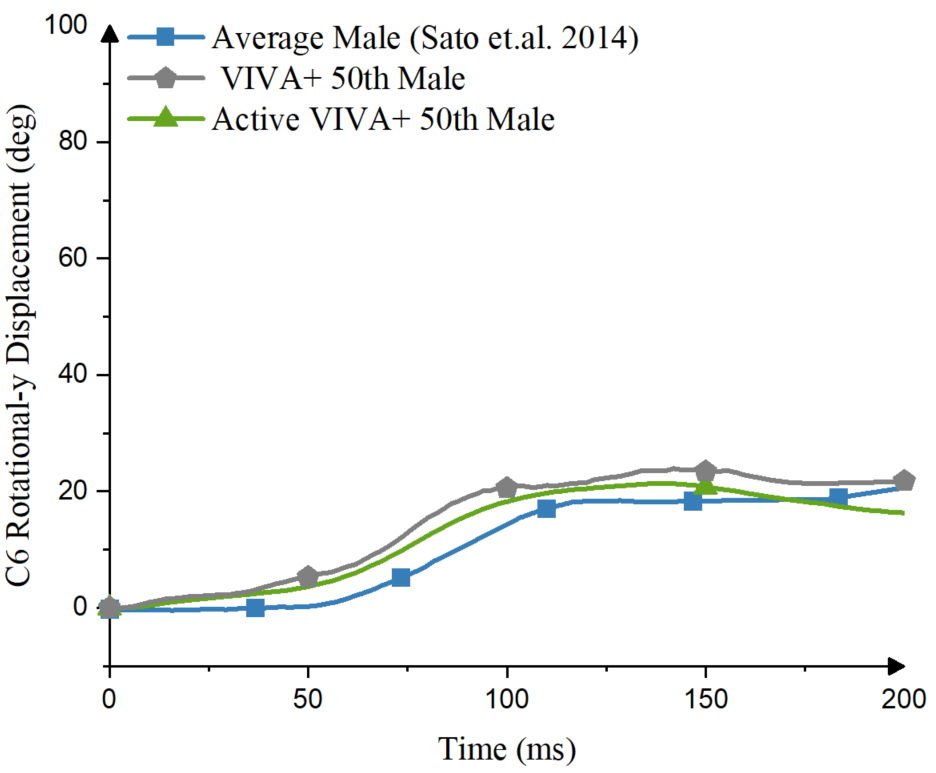


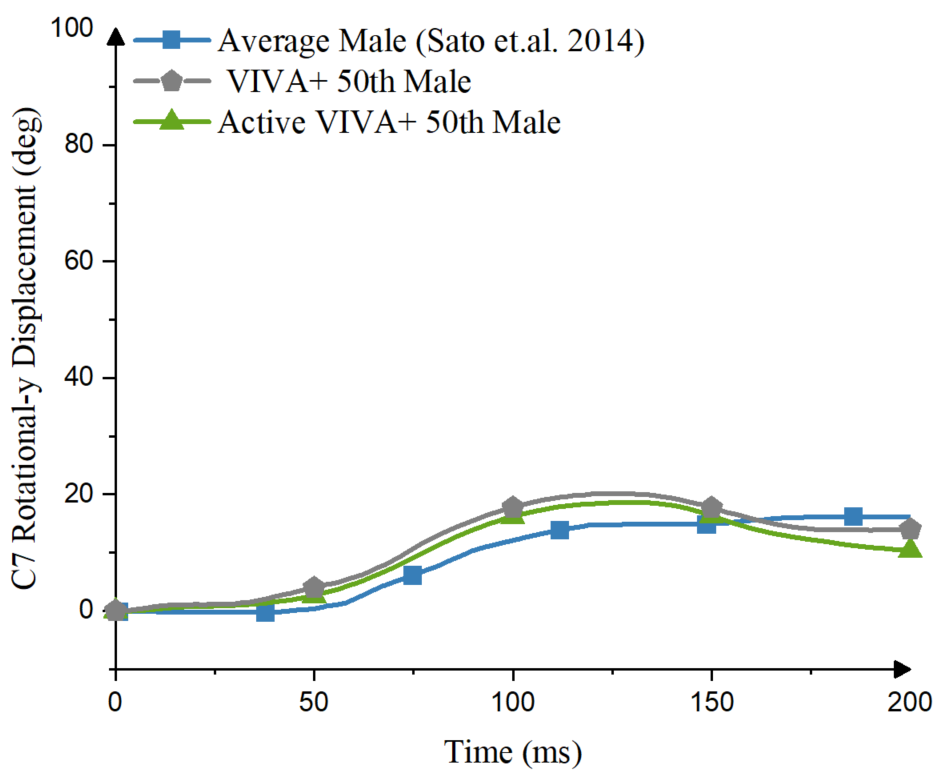


**Supplementary Figure 1.** Comparison of Head C.G and Cervical Vertebra C.G linear and rotational displacements between Original VIVA+ Male Model, Active Male VIVA+ Models and Volunteer Kinematics from Sato et al. (2014) 5.8km/h


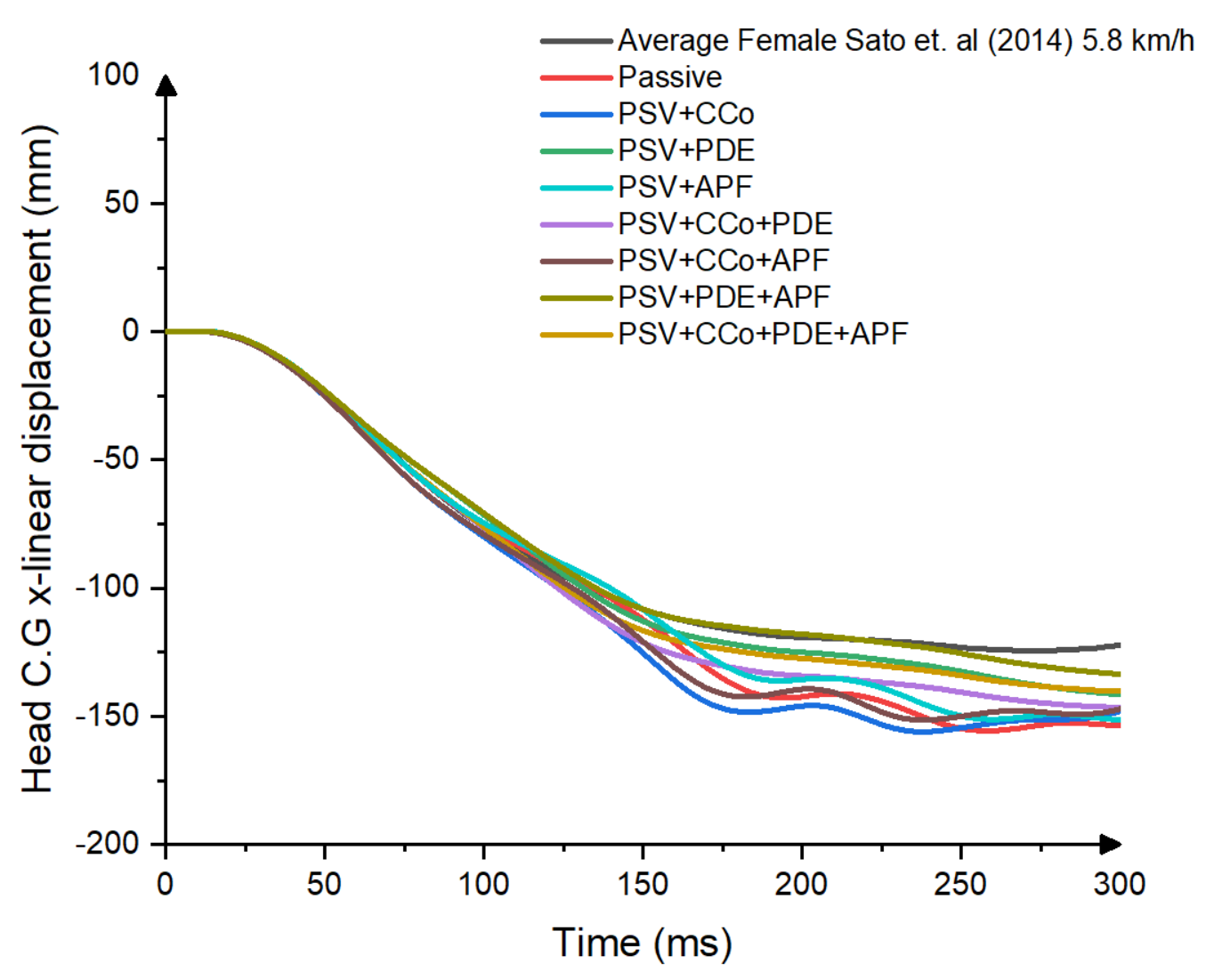

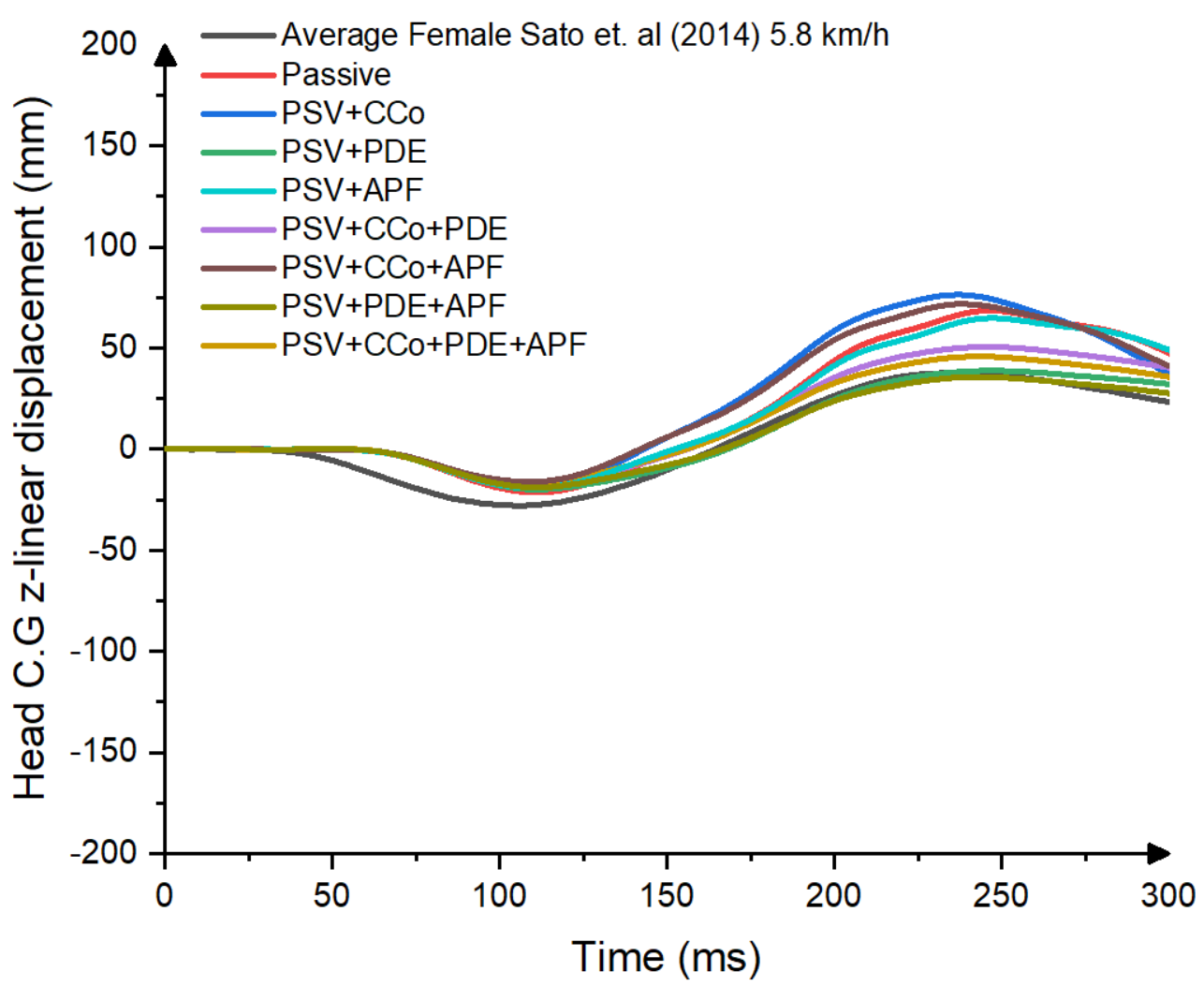

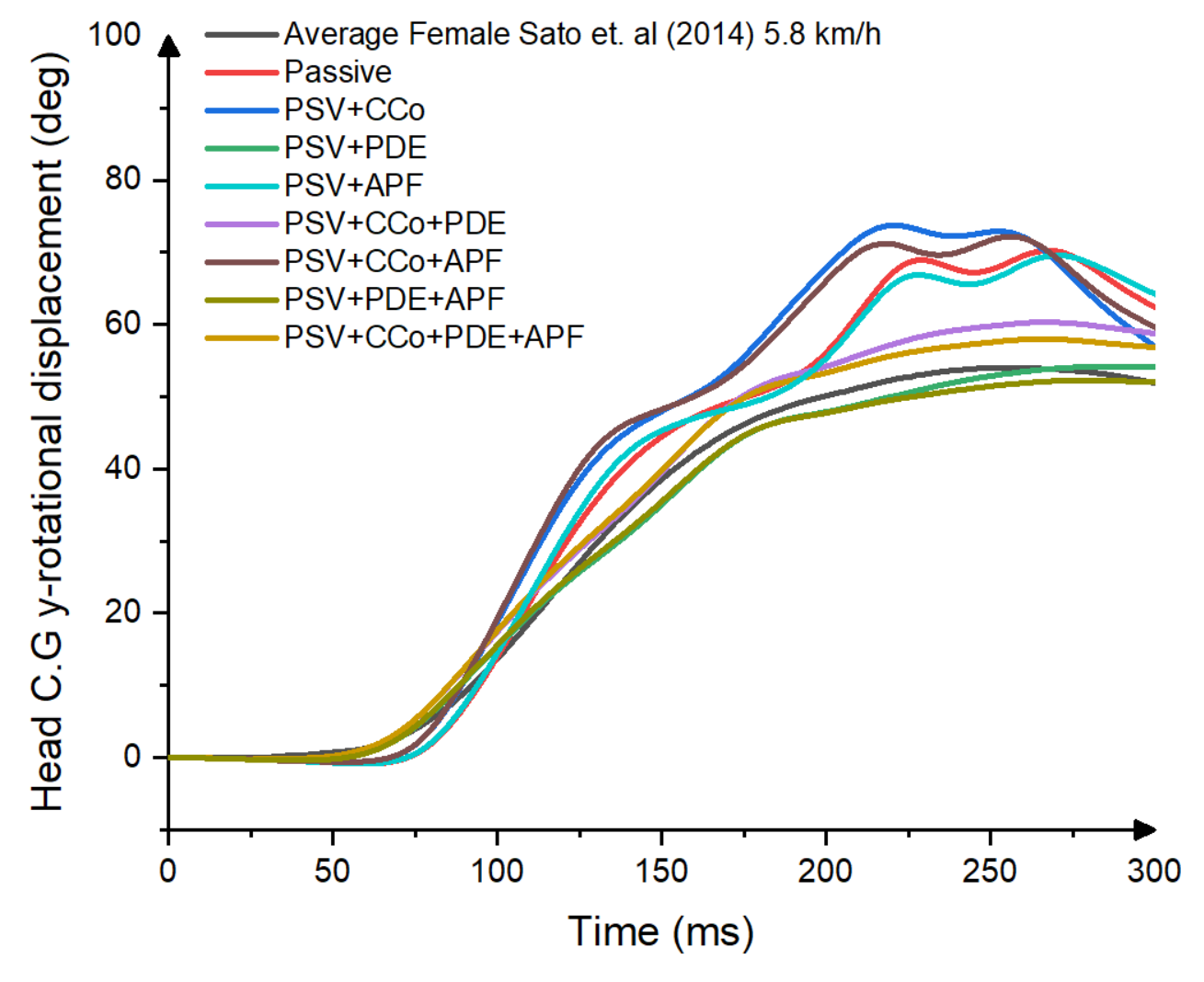


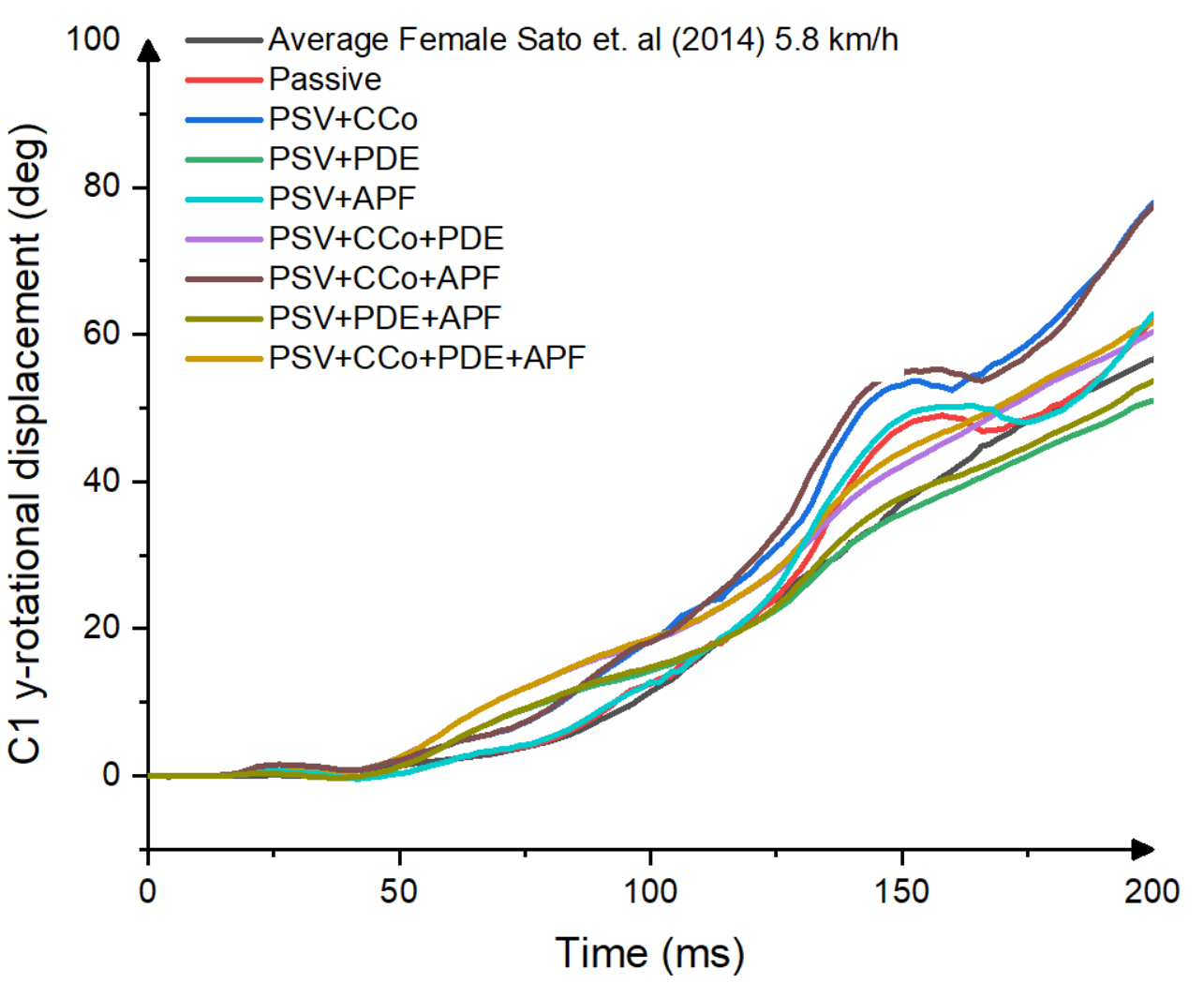

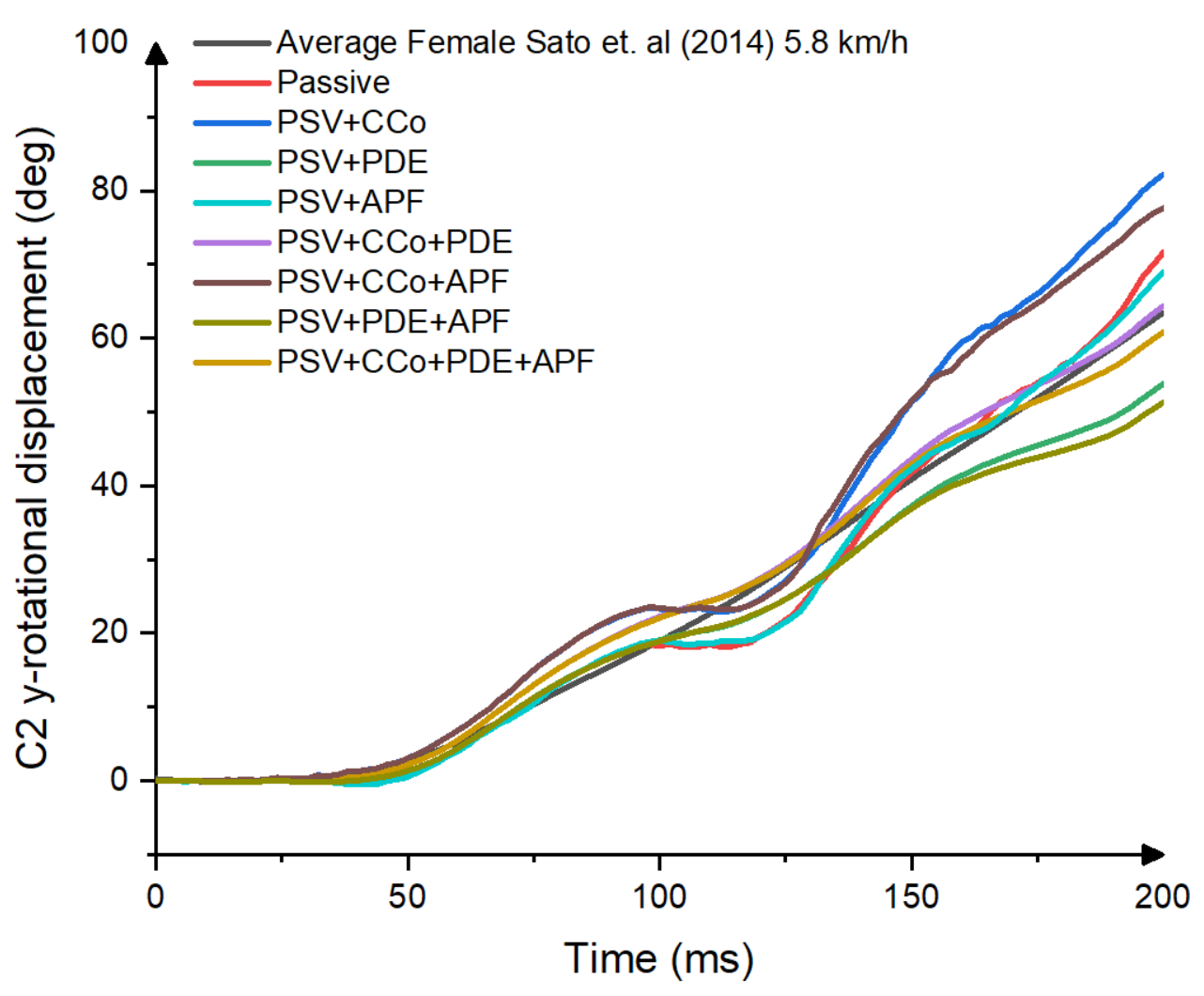

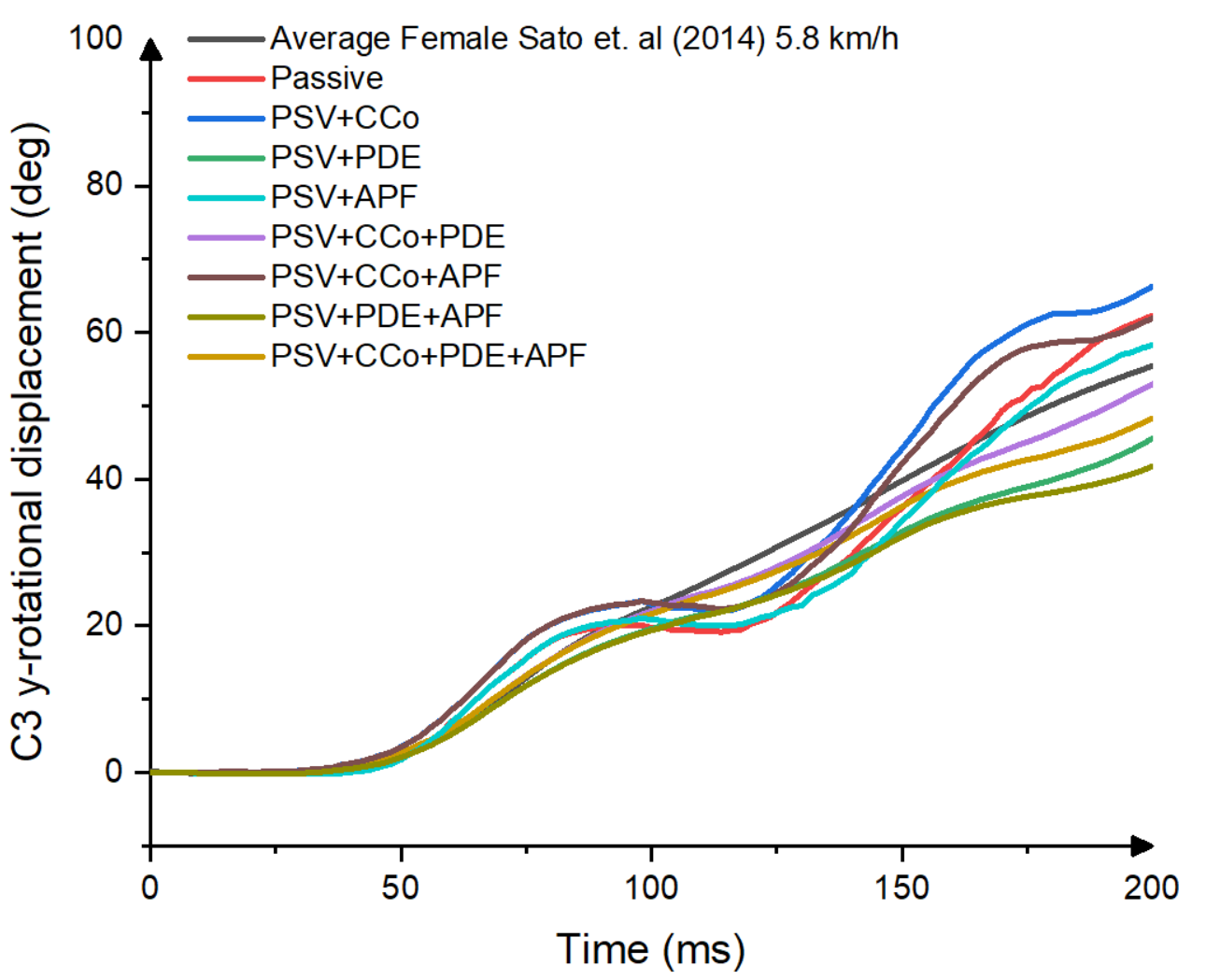


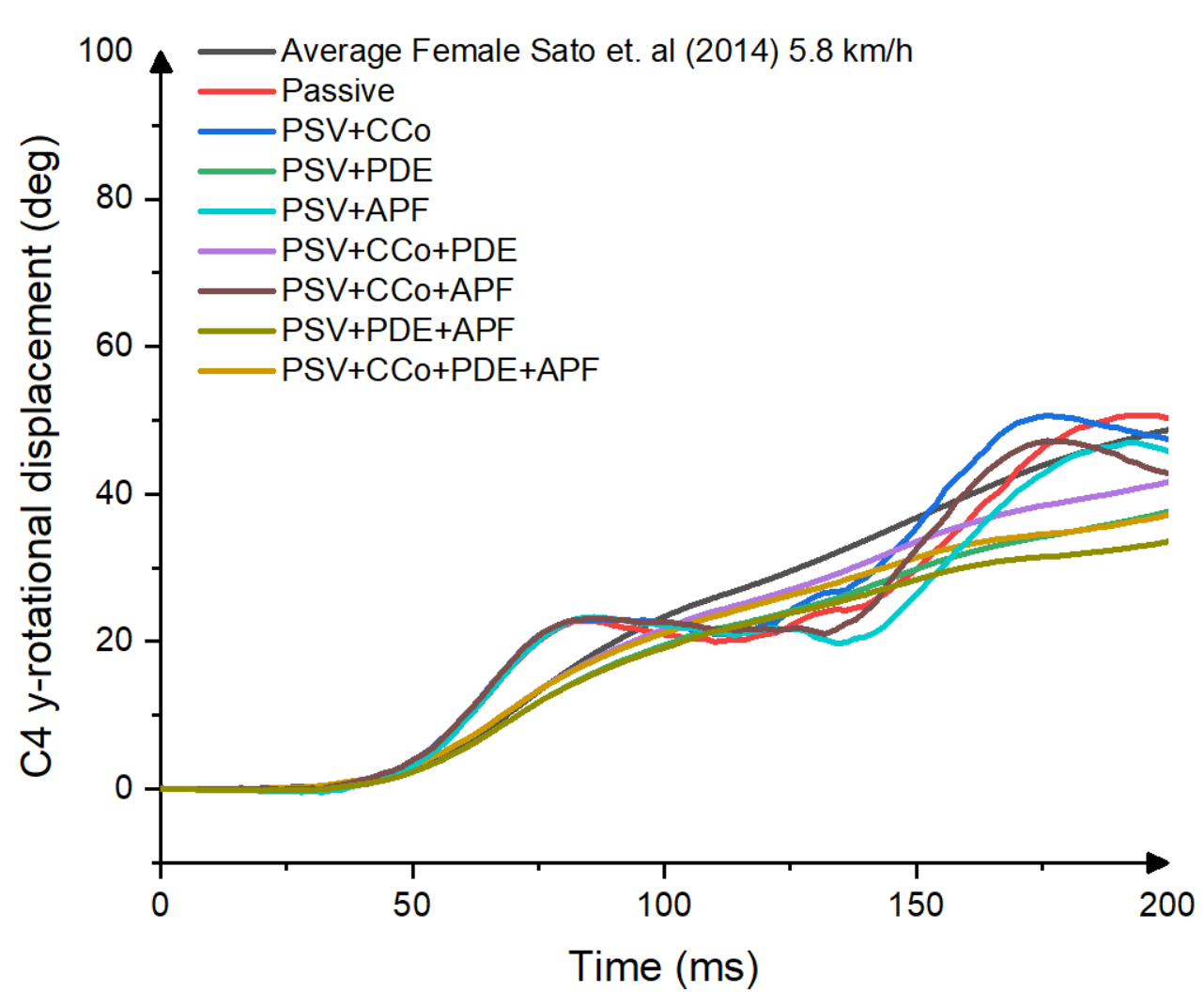

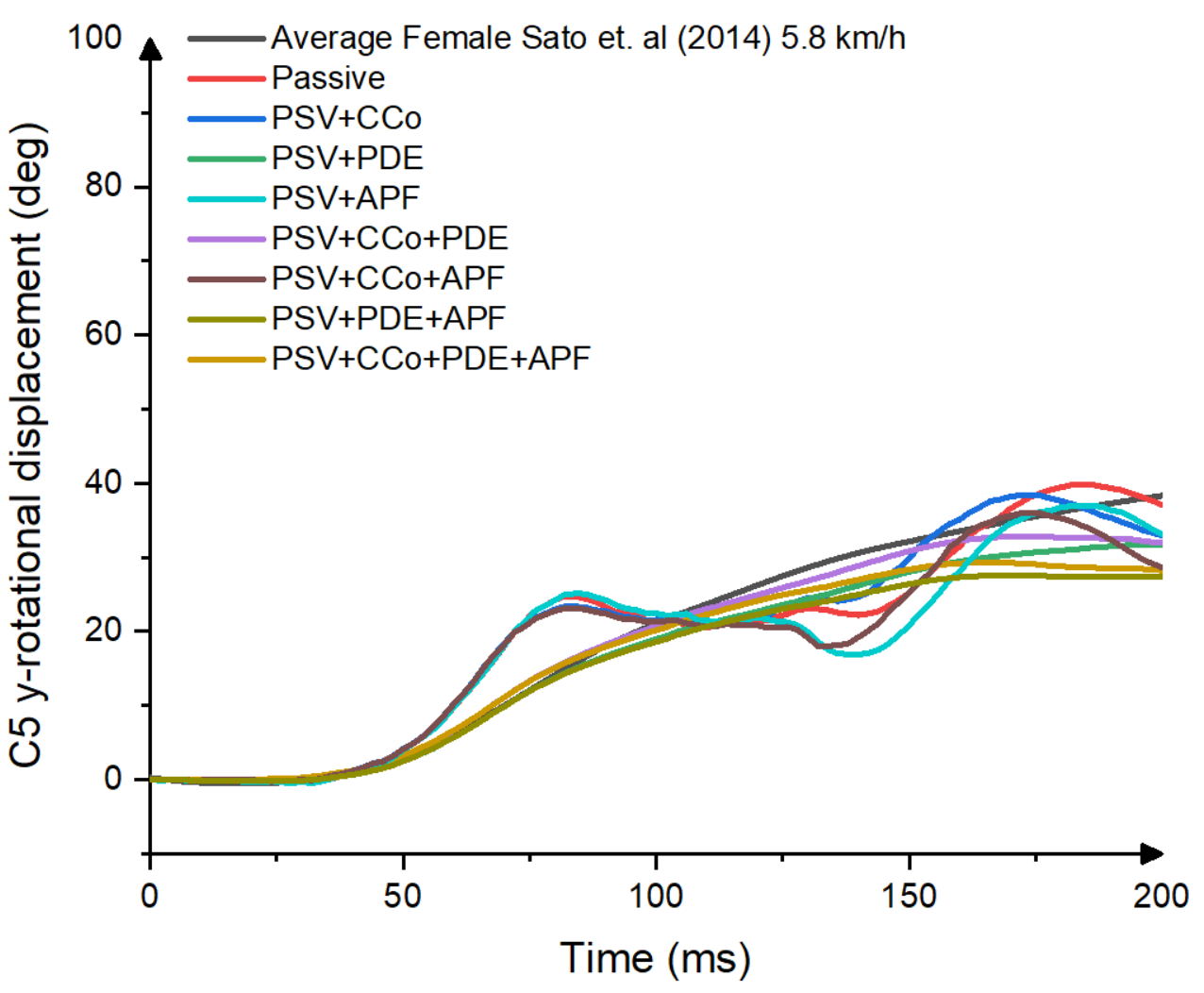

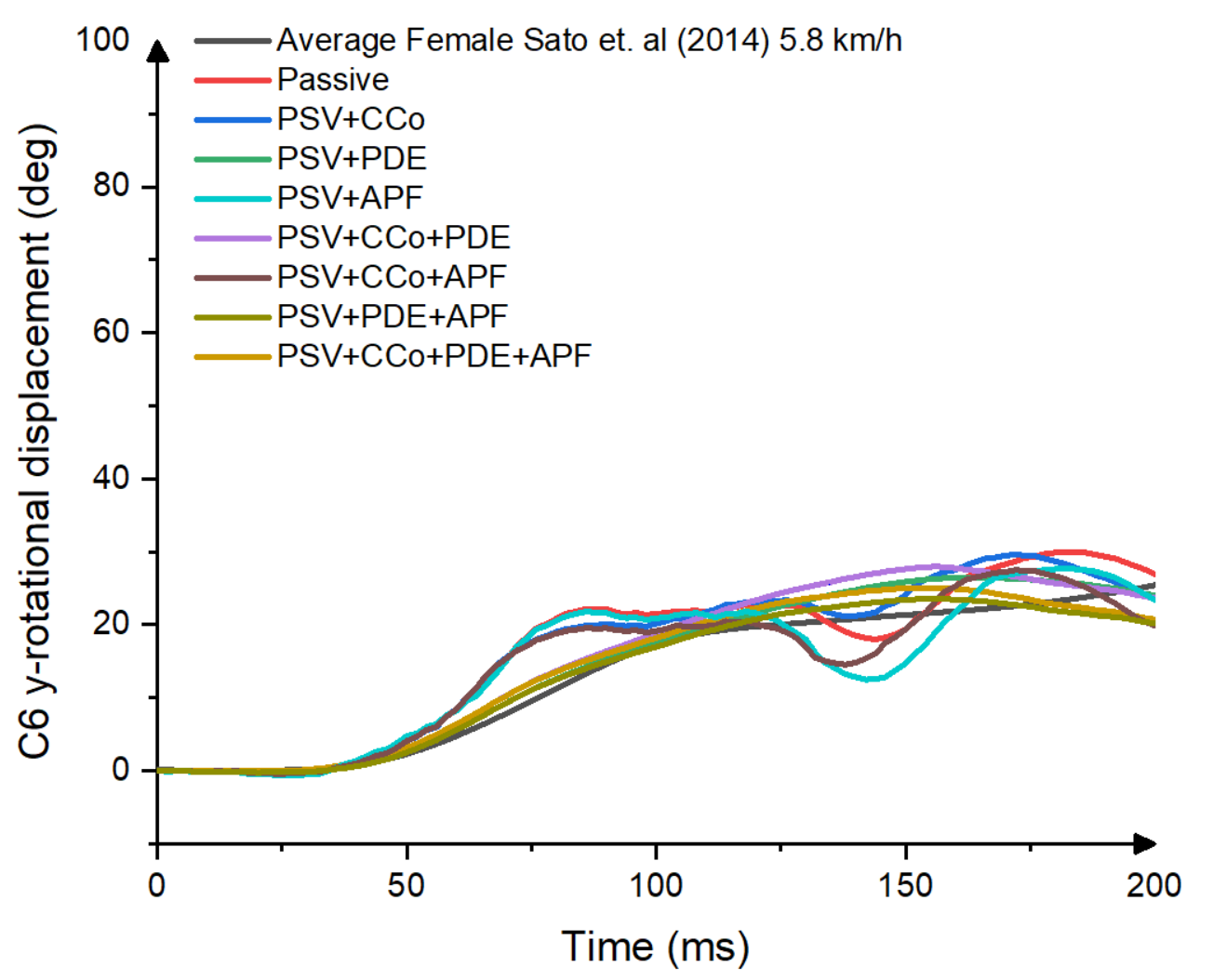


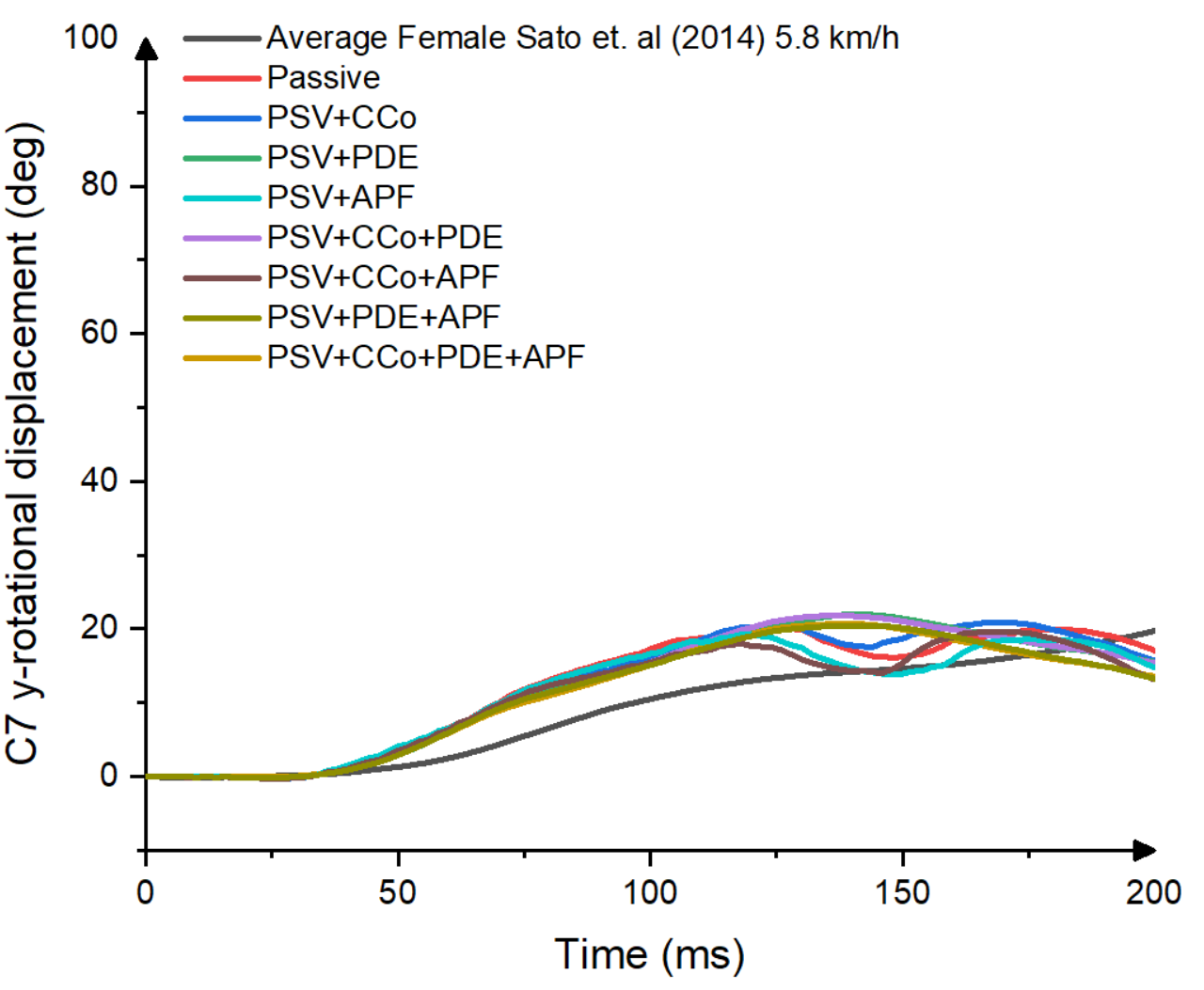


**Supplementary Figure 2.** Comparison of Head C.G and Cervical Vertebra C.G linear and rotational displacements of Active VIVA+ Female Model with Various Complexities and Volunteer Kinematics from Sato et al. (2014) 5.8km/h


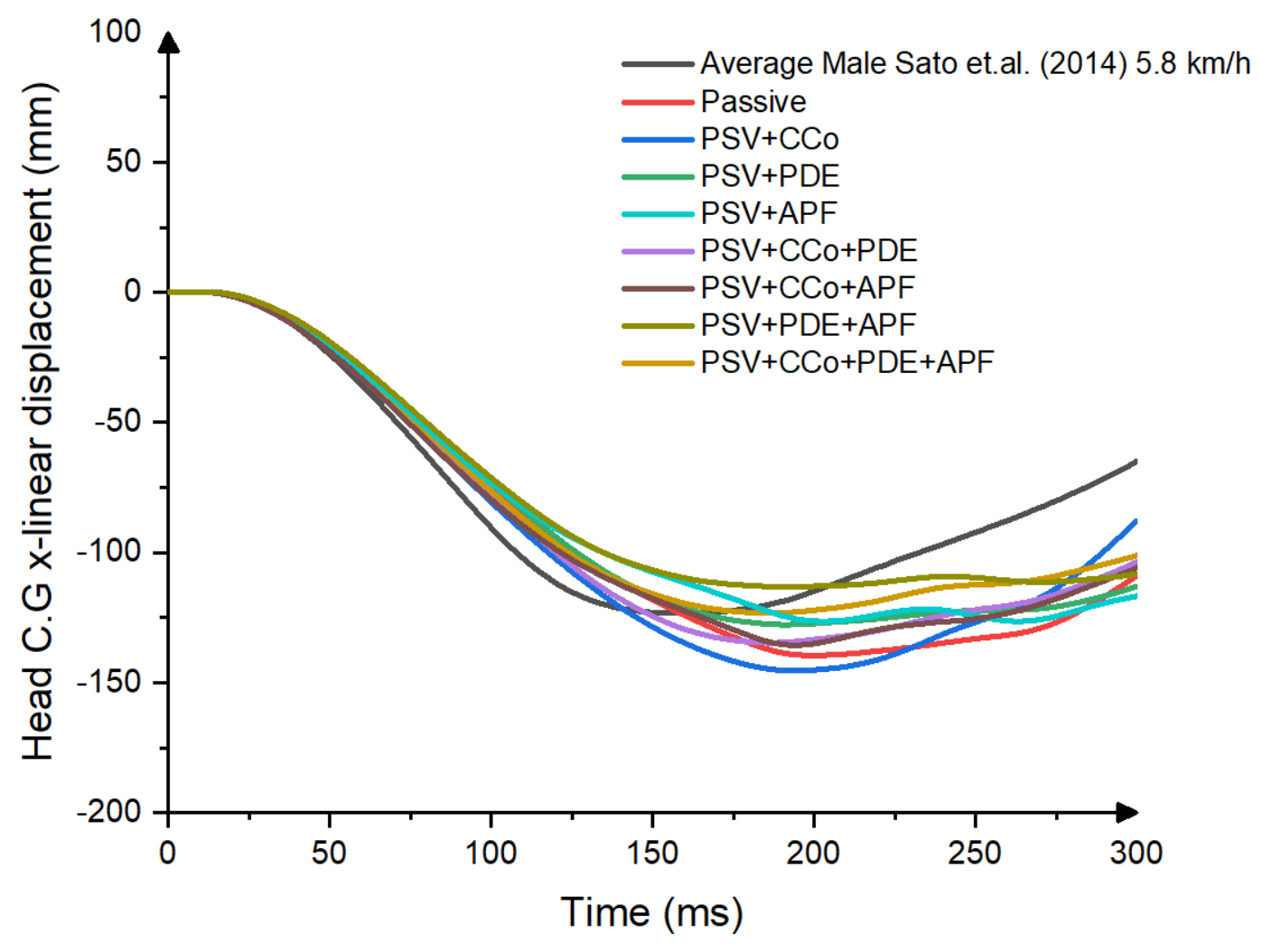

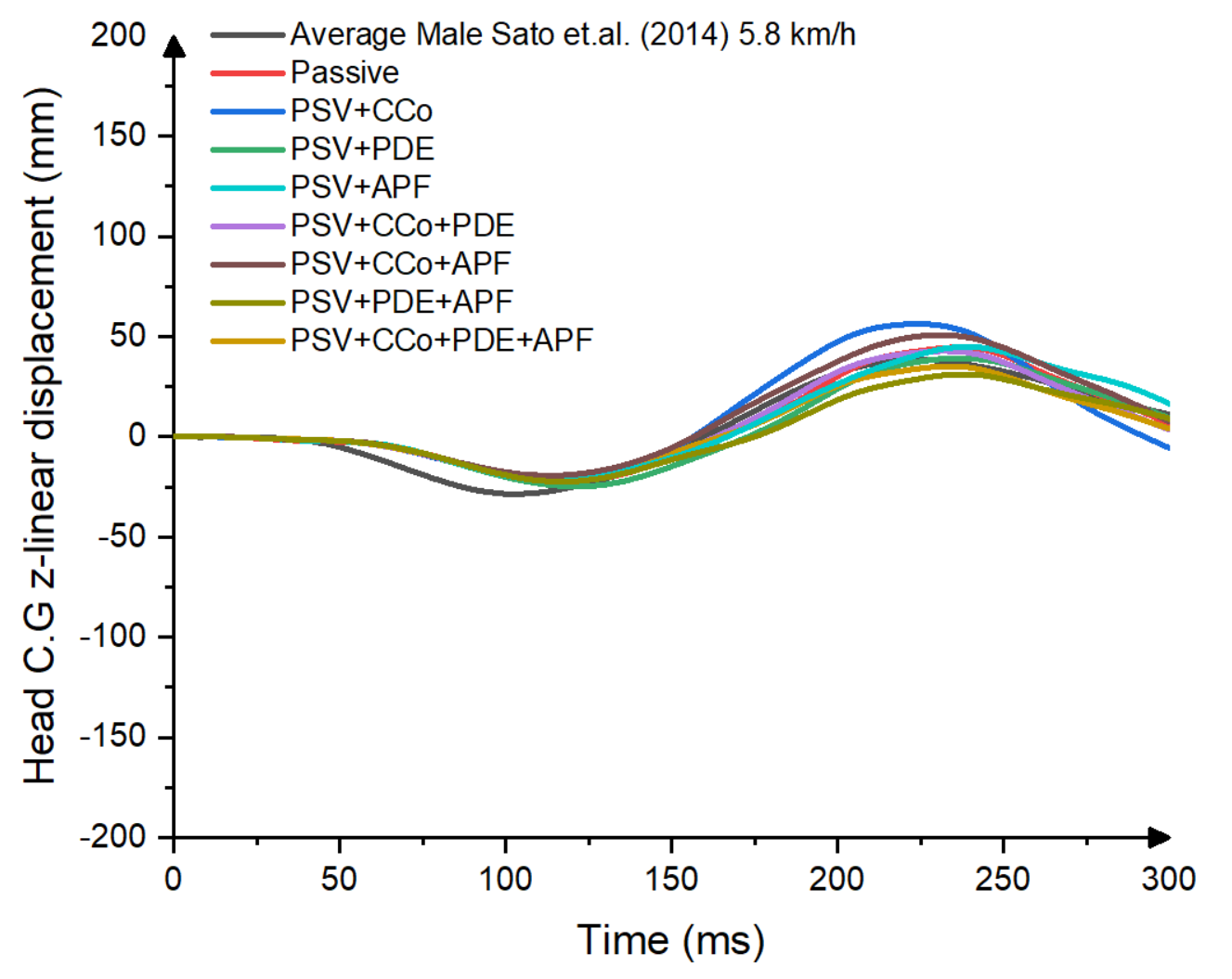

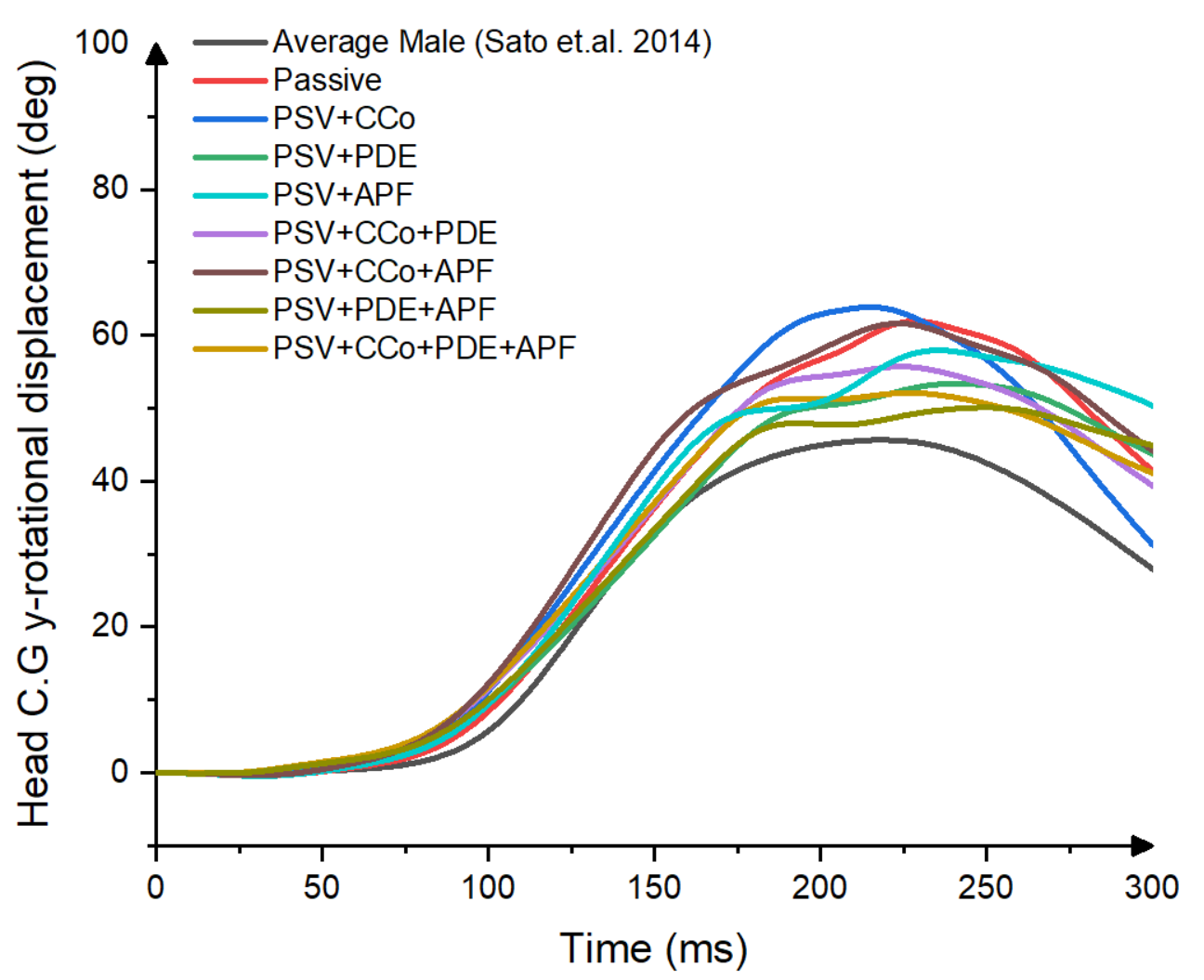


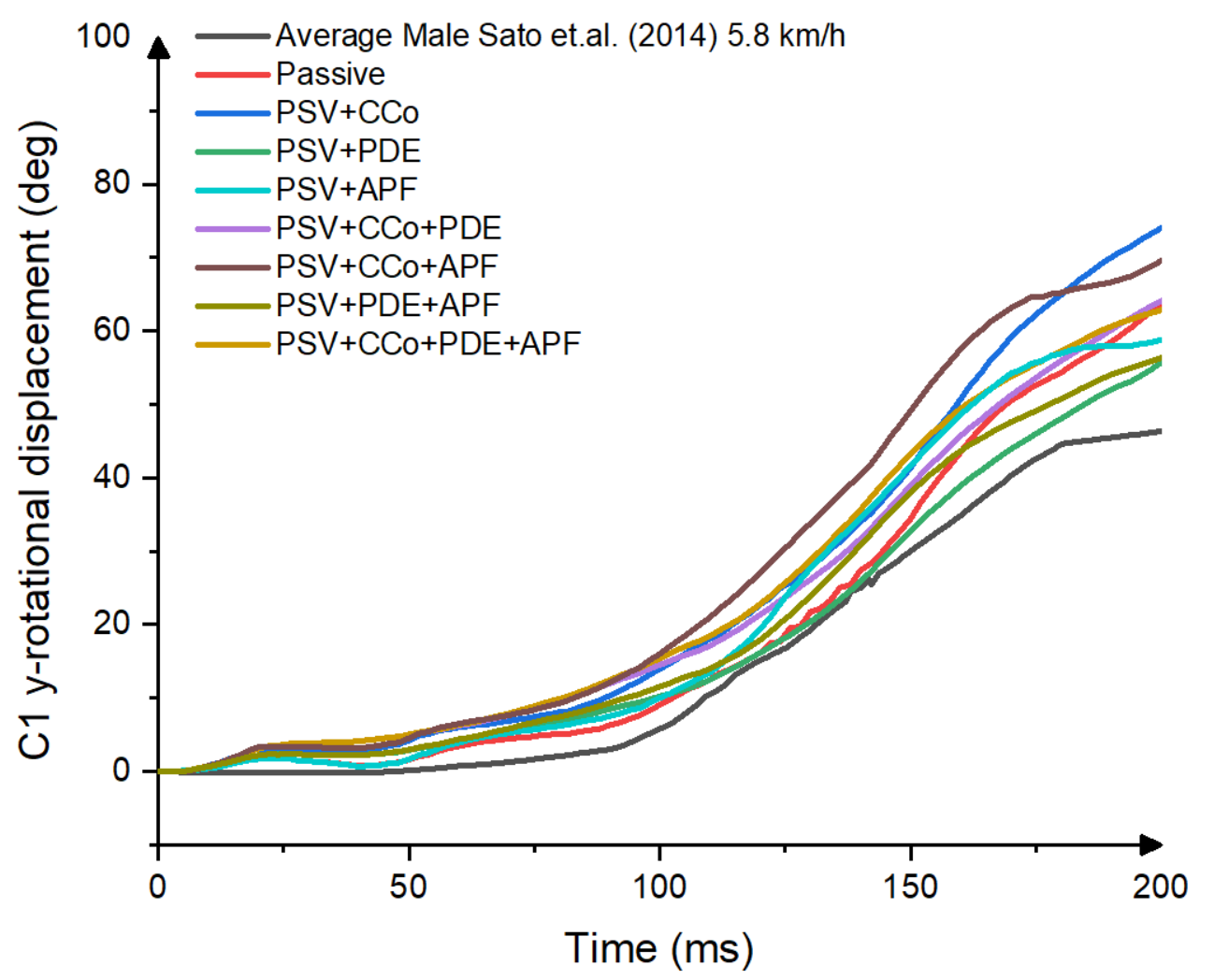

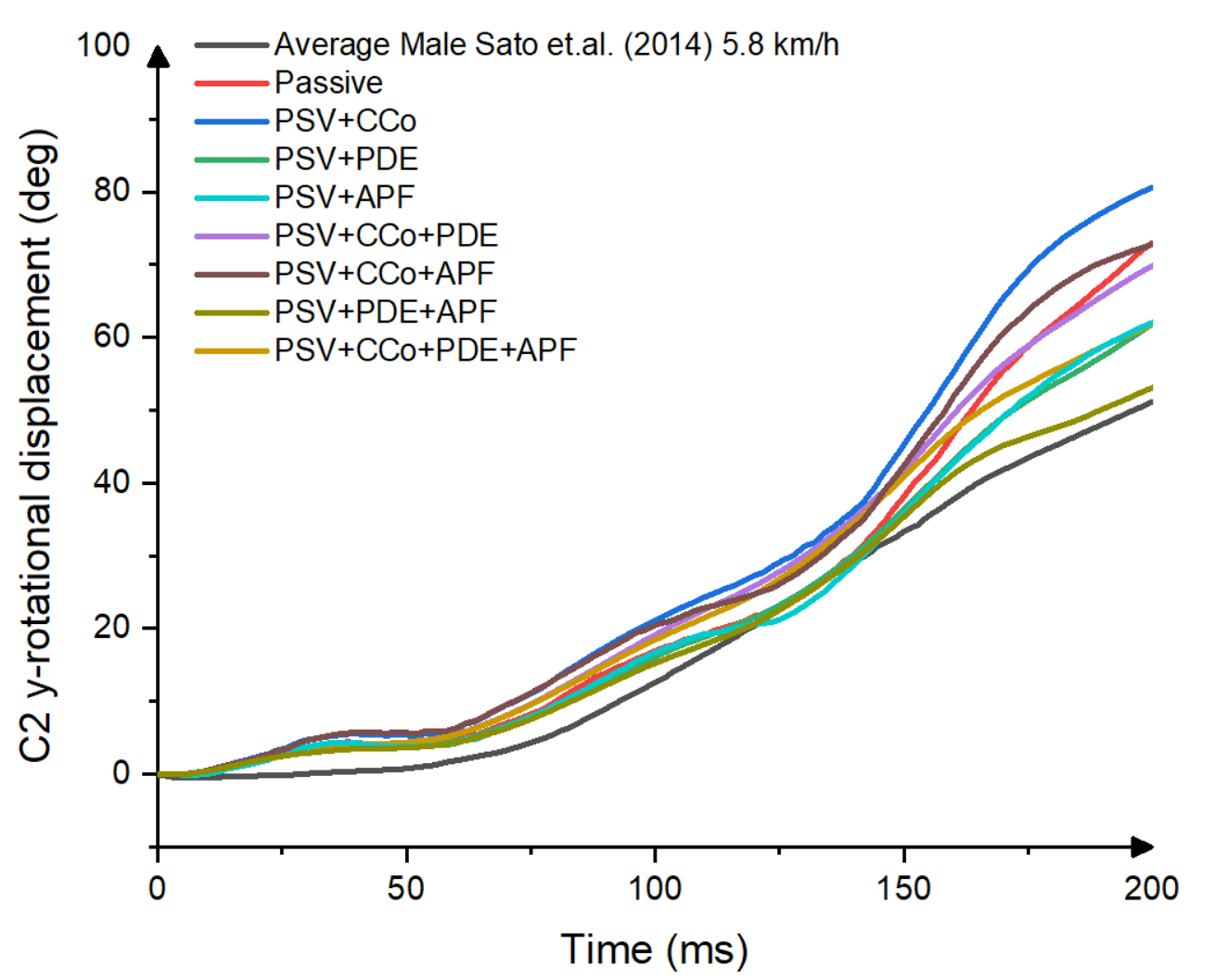

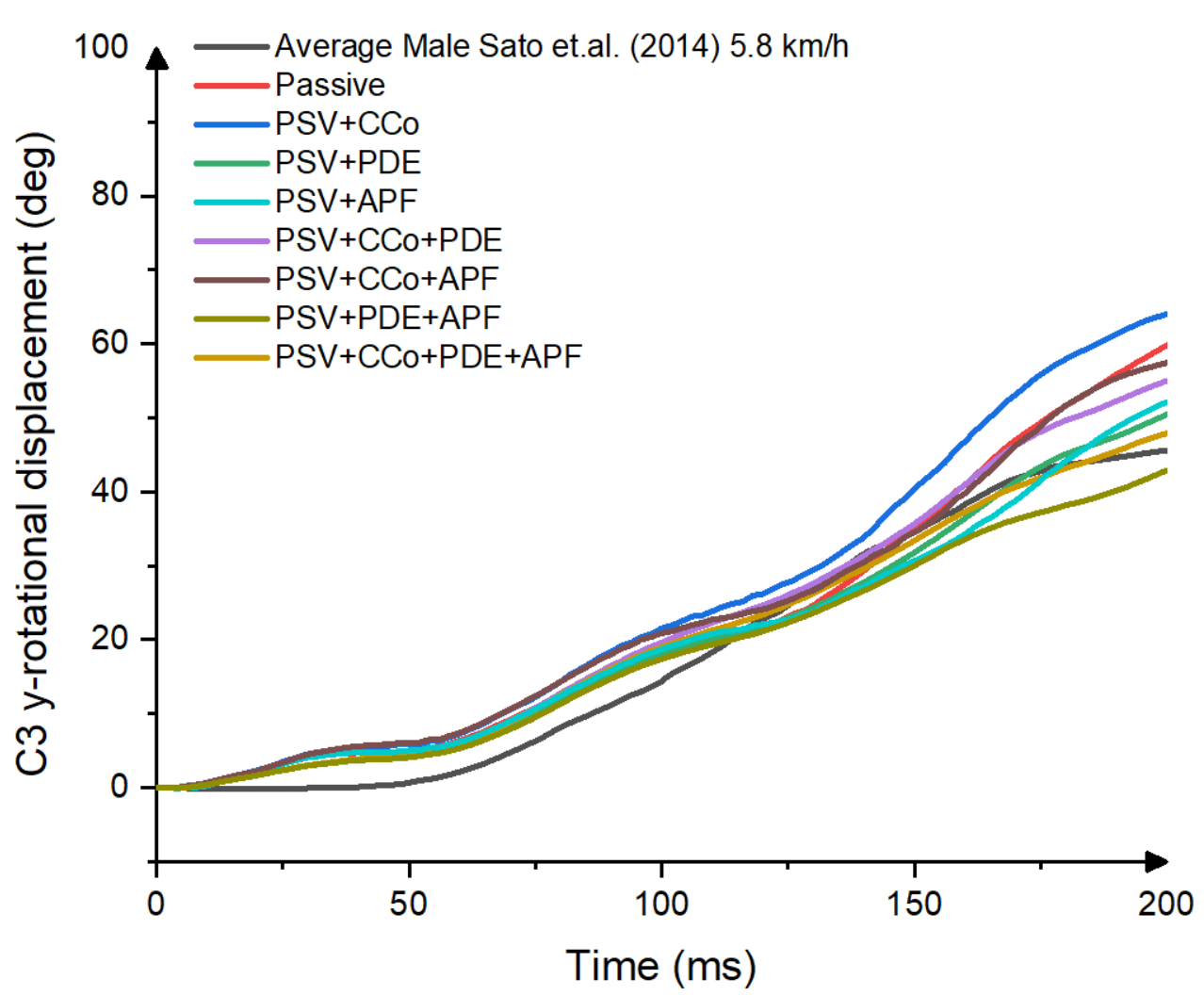


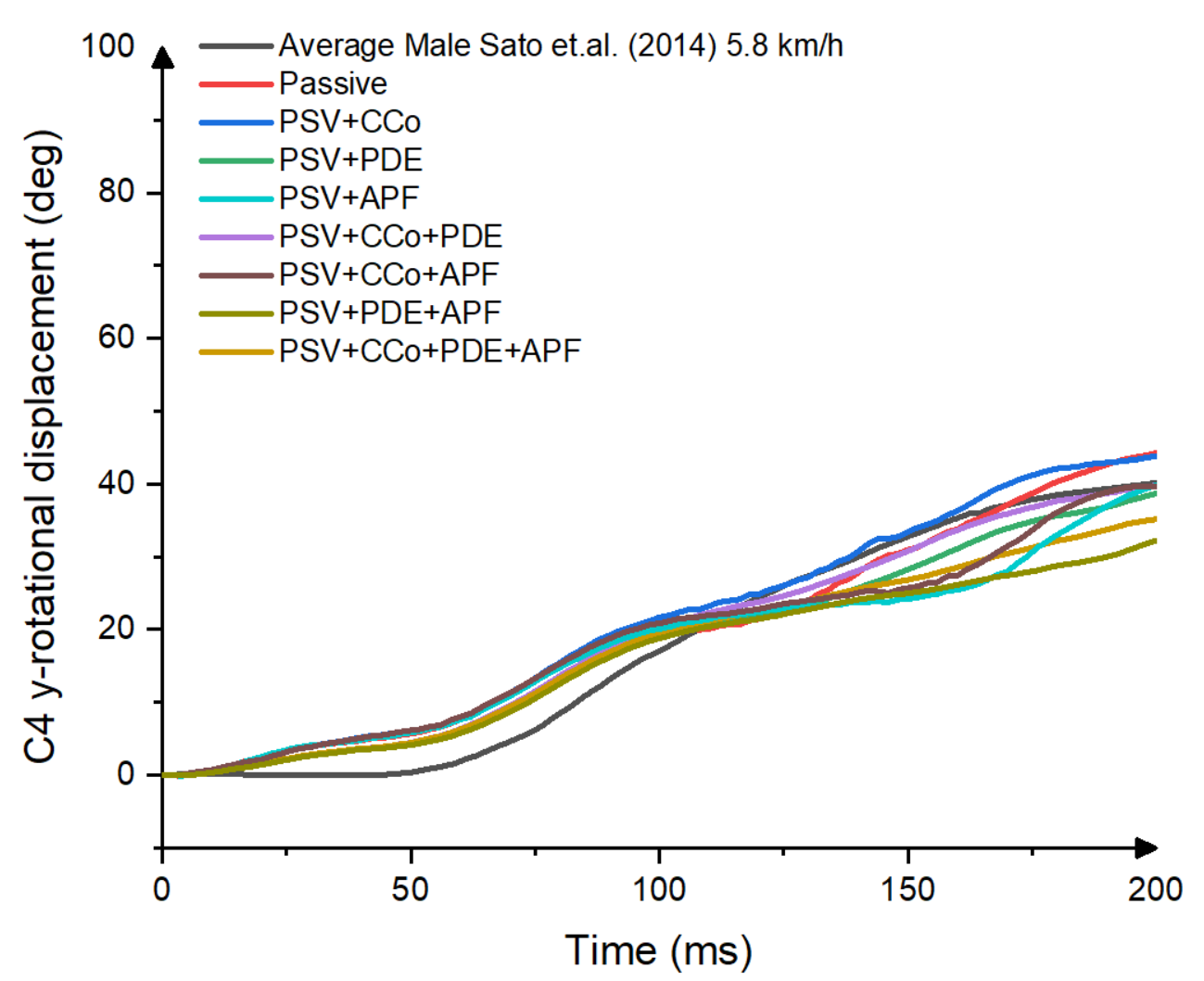

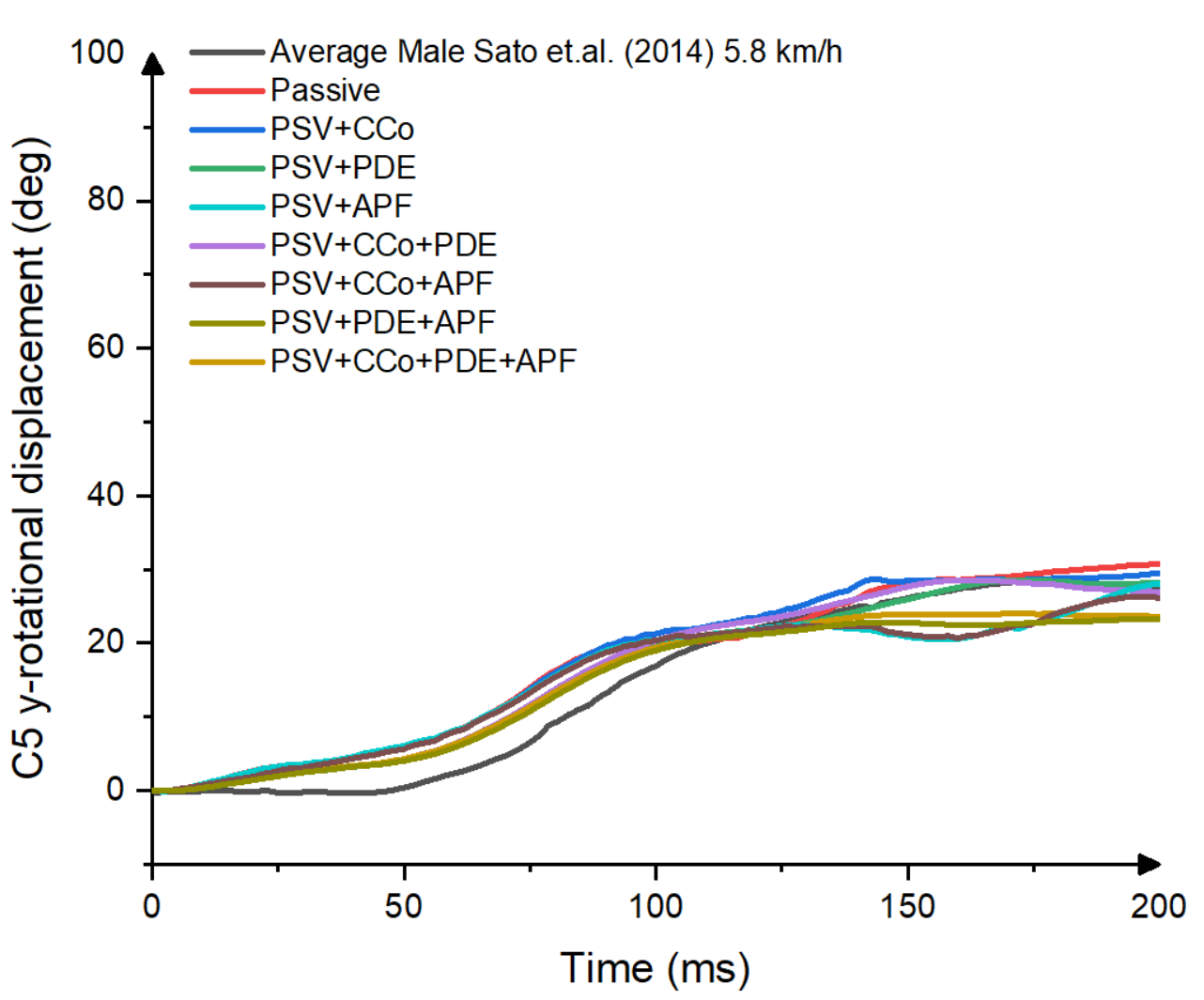

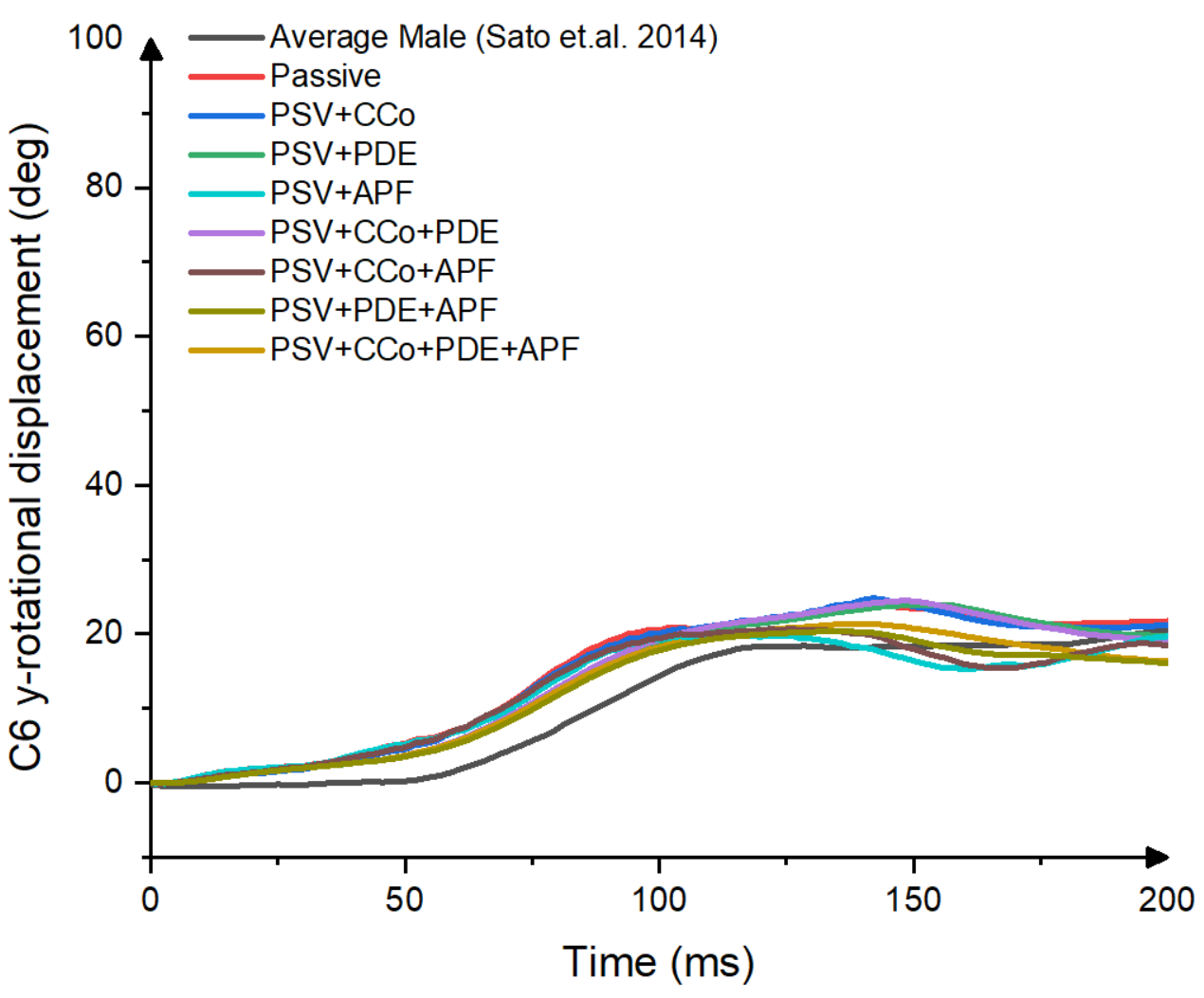


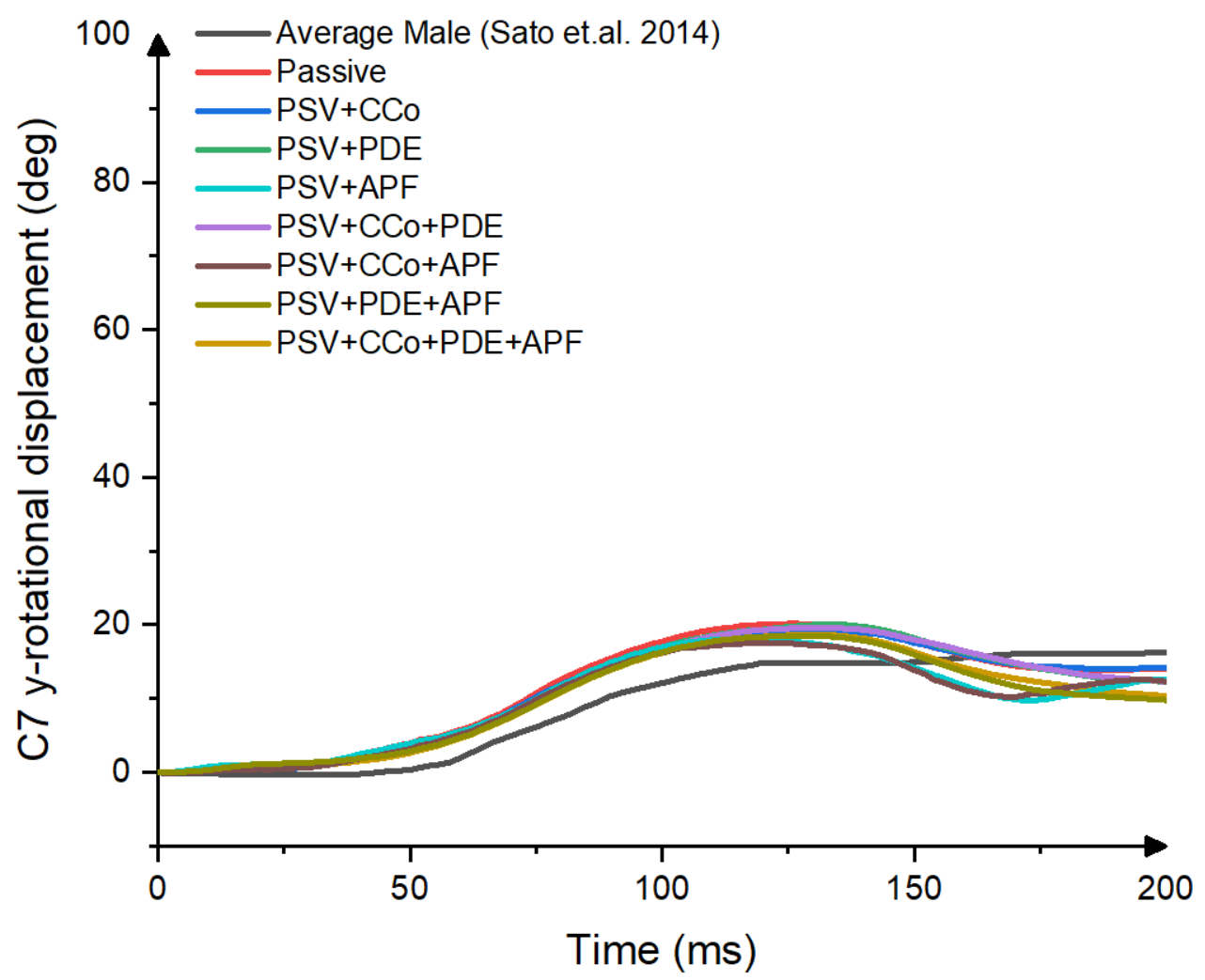


**Supplementary Figure 3.** Comparison of Head C.G and Cervical Vertebra C.G linear and rotational displacements of Active VIVA+ Male Model with Various Complexities and Volunteer Kinematics from Sato et al. (2014) 5.8km/h


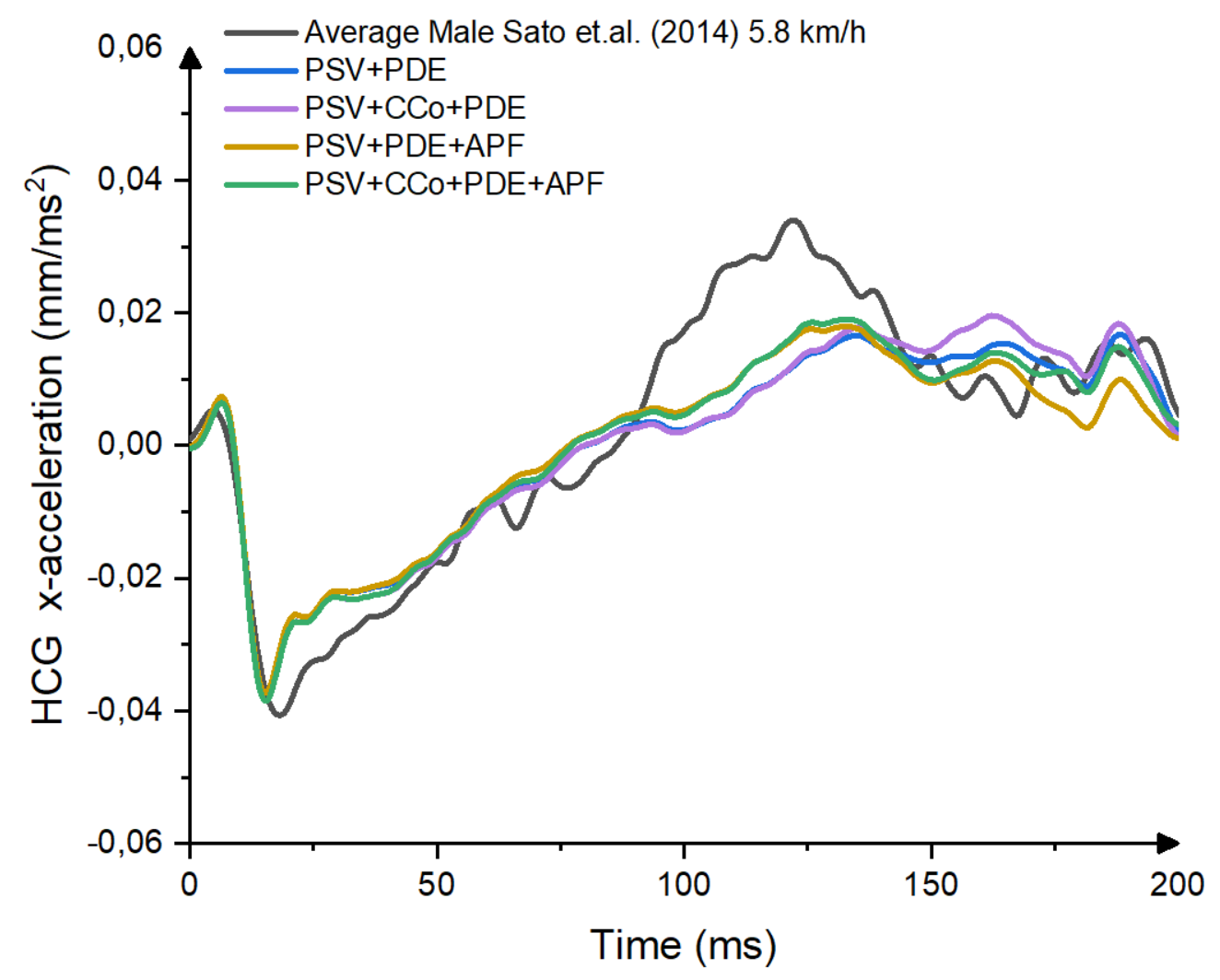

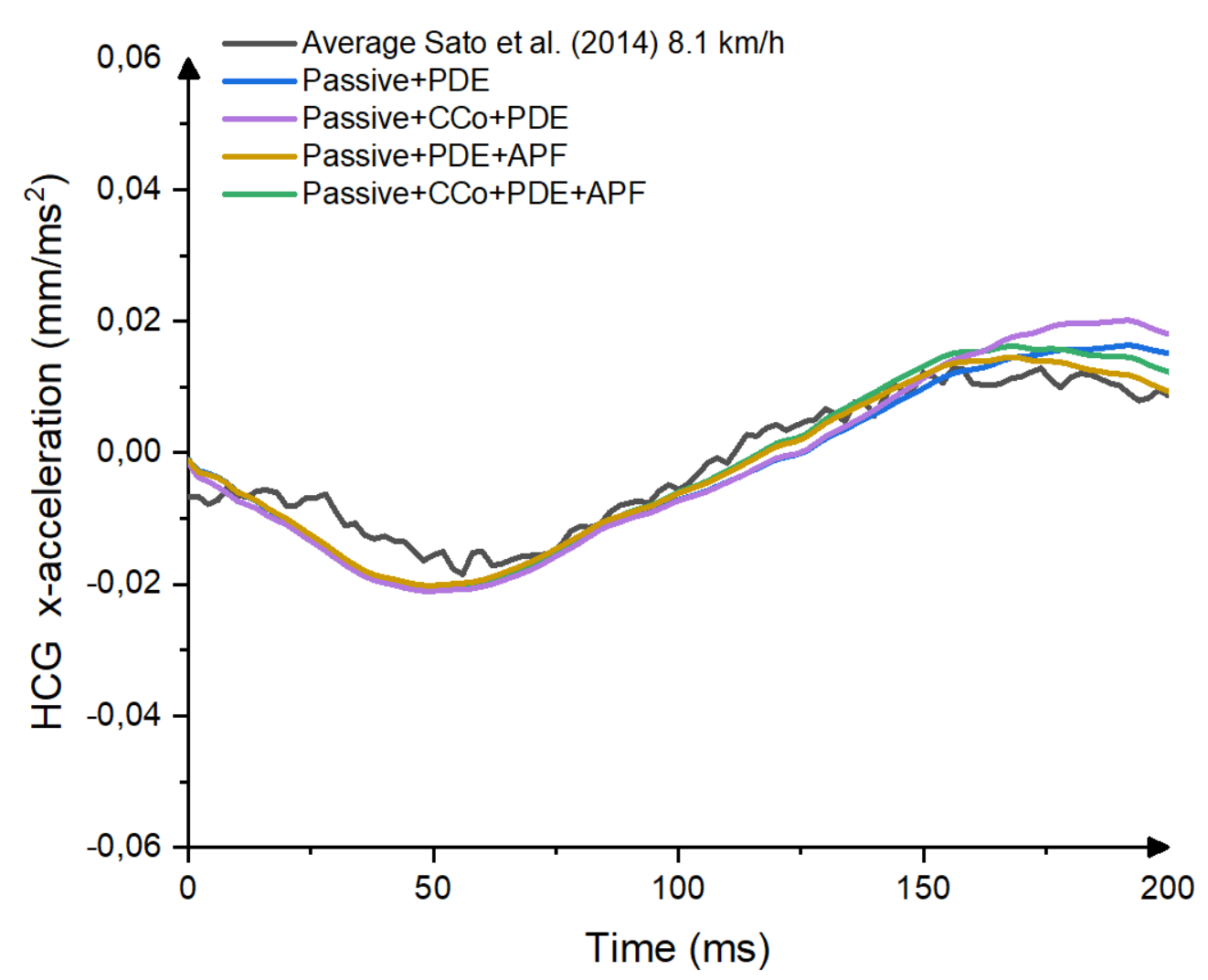

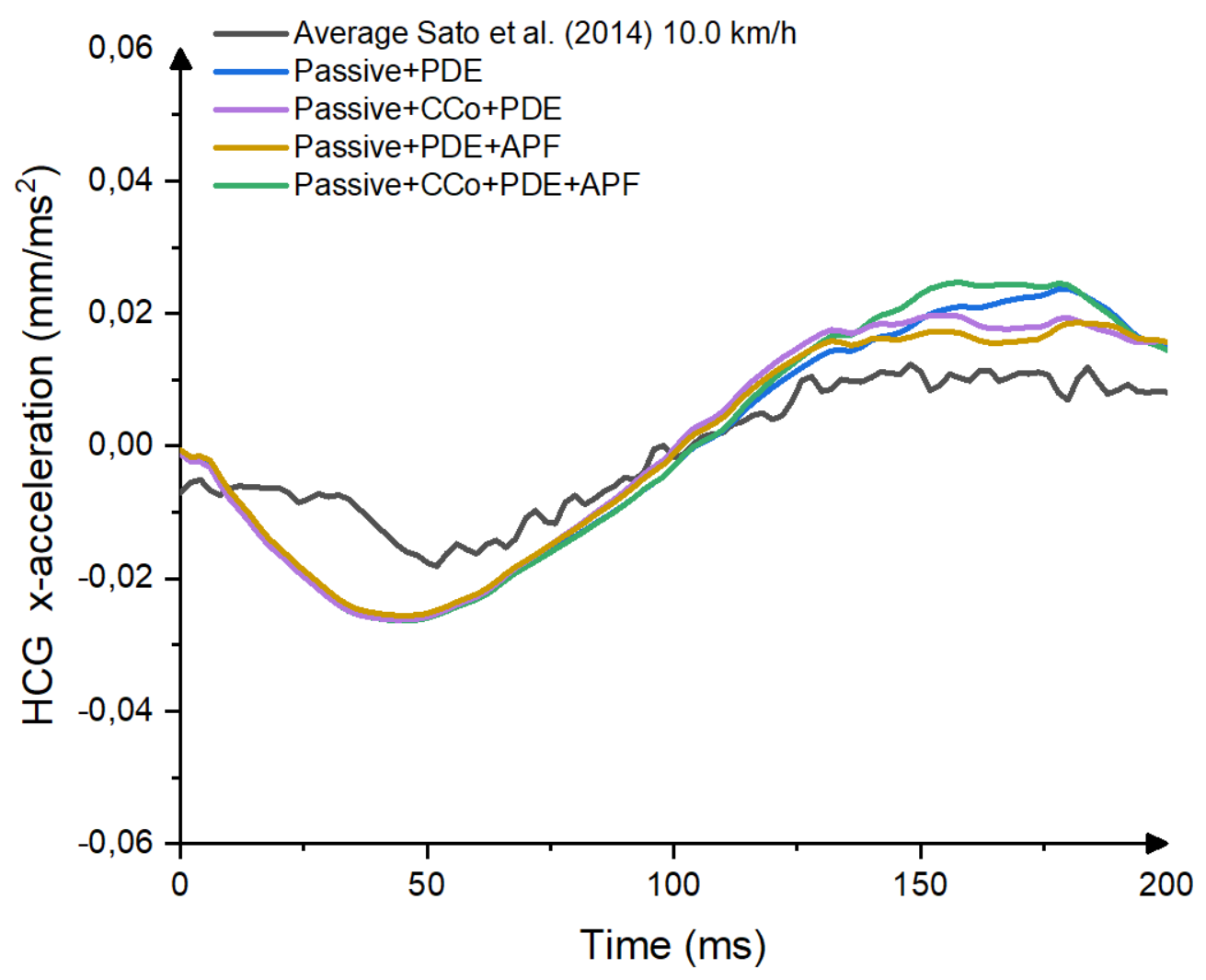


**Supplementary Figure.** 4 Comparison of Head C.G x-Acceleration between male Models with Various Complexities and Volunteer Kinematics from Sato et al. (2014) 5.8km/h, Sato et al. (2014) 8.1km/h, and Sato et al. (2014) 10km/h.


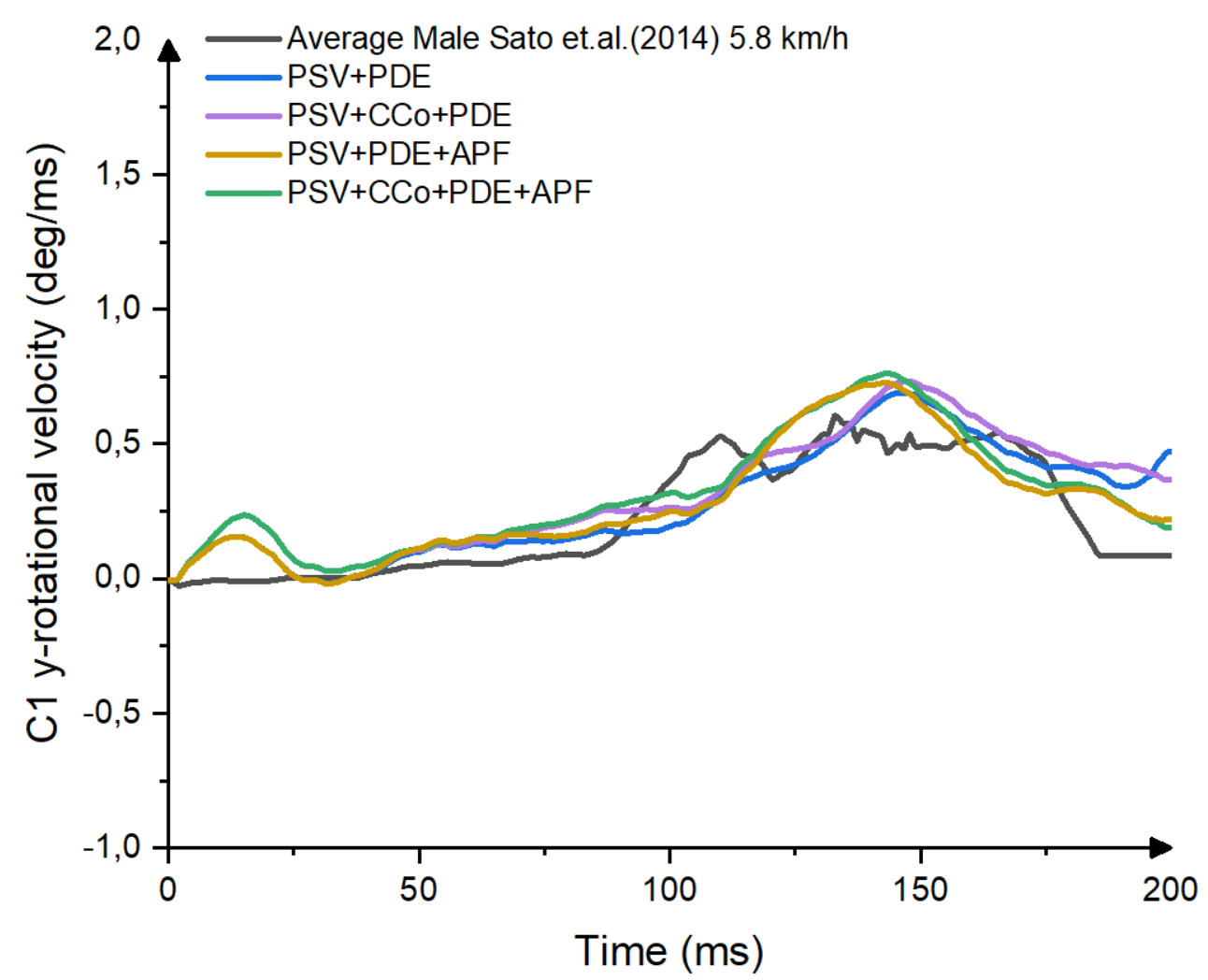

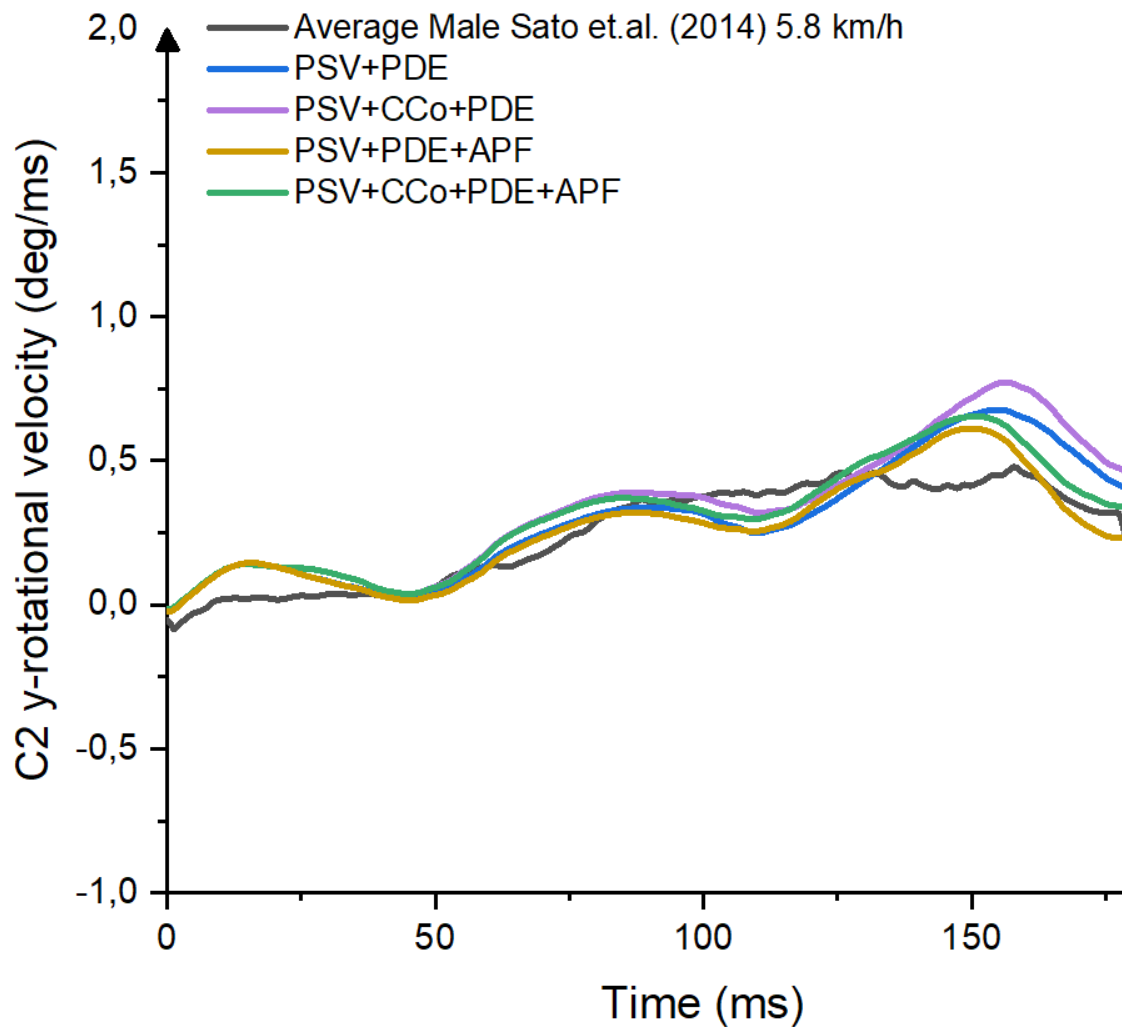

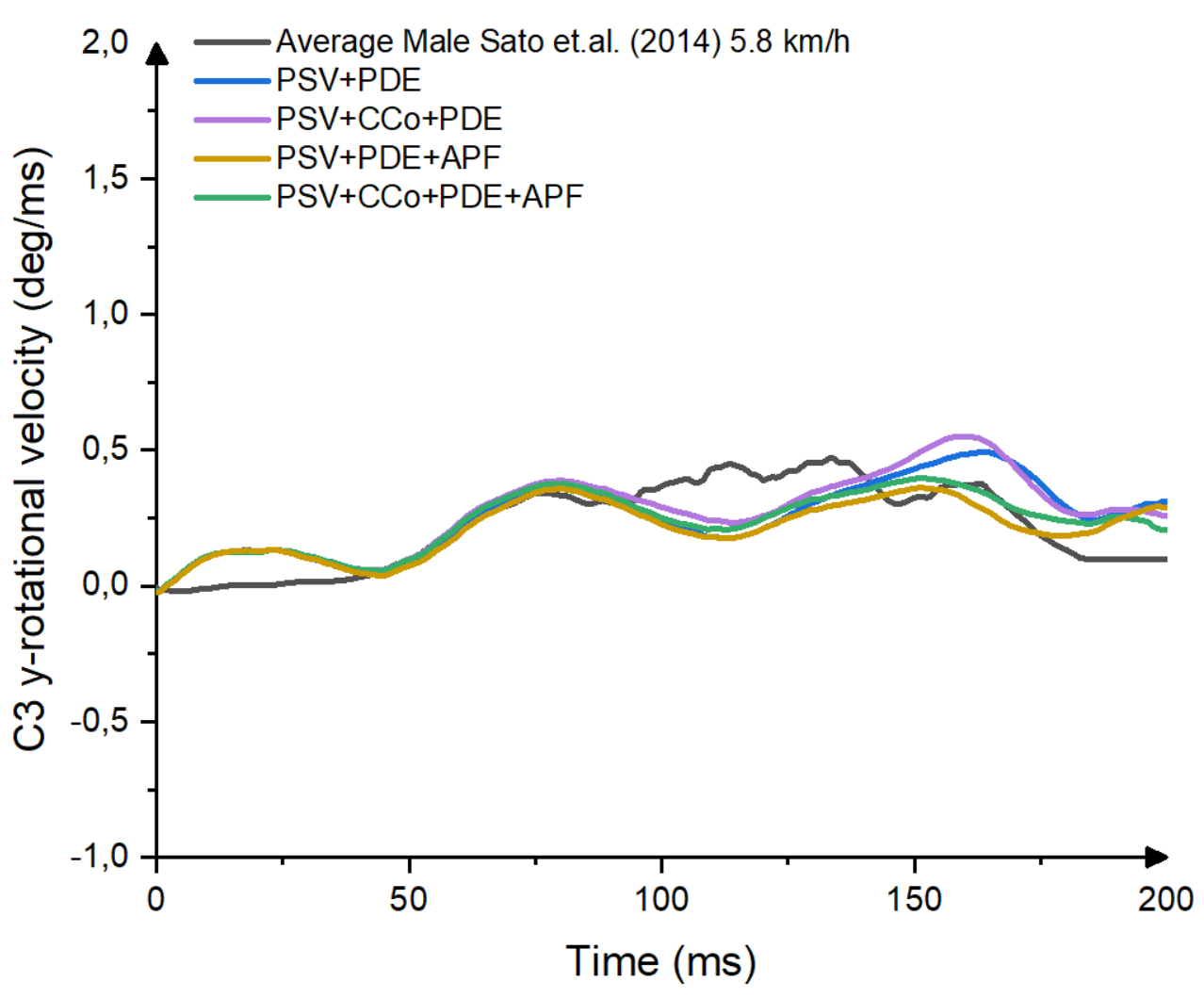


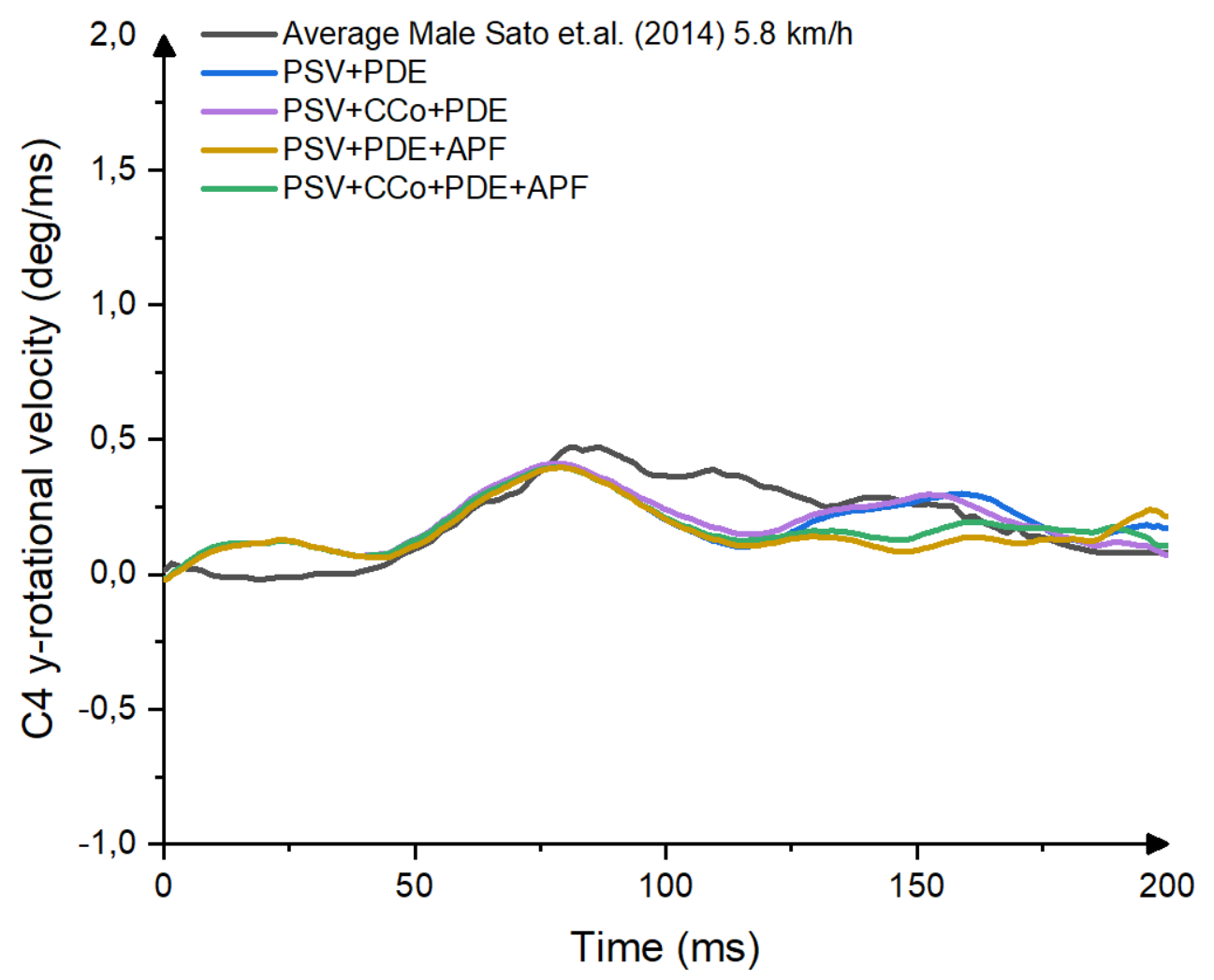

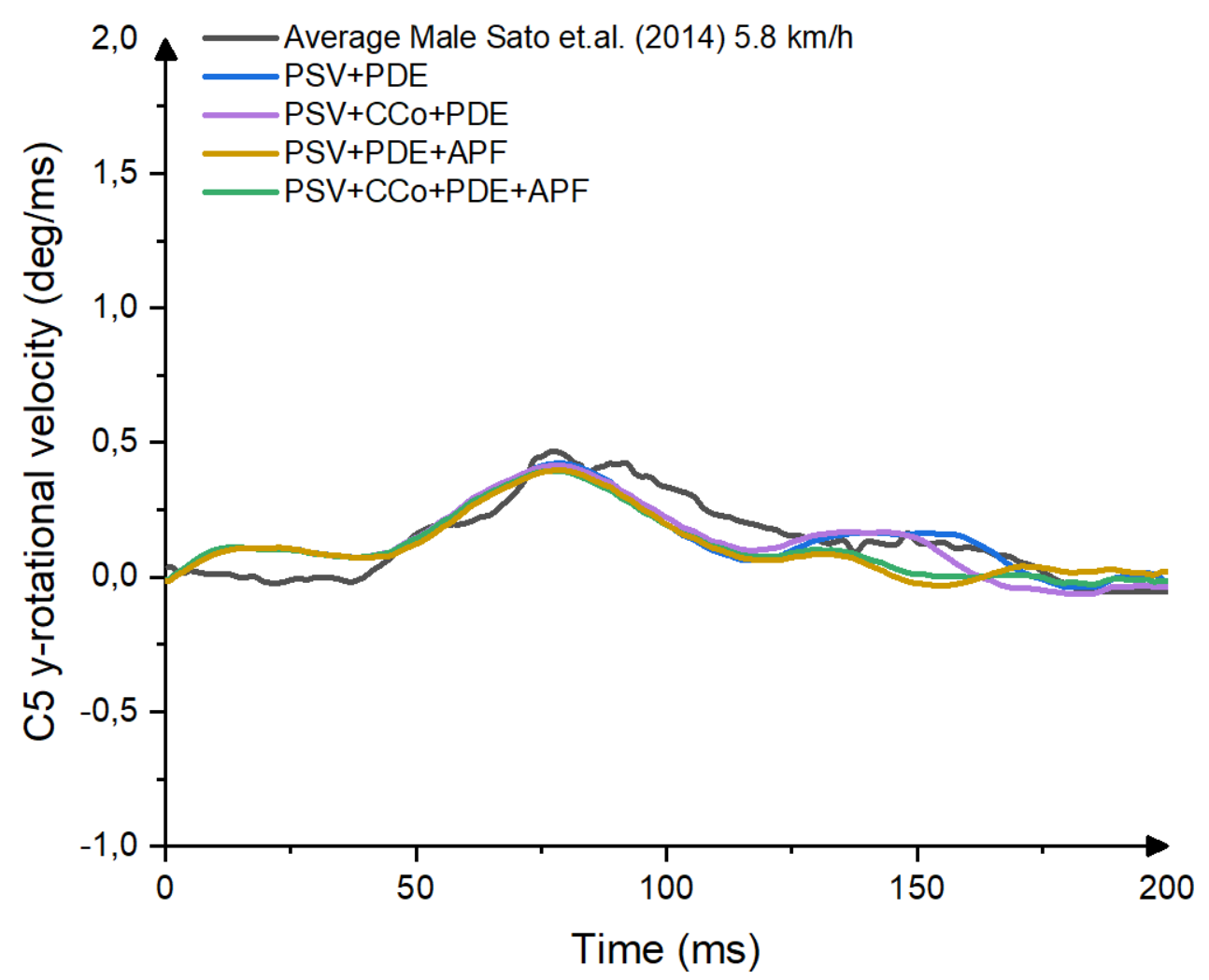

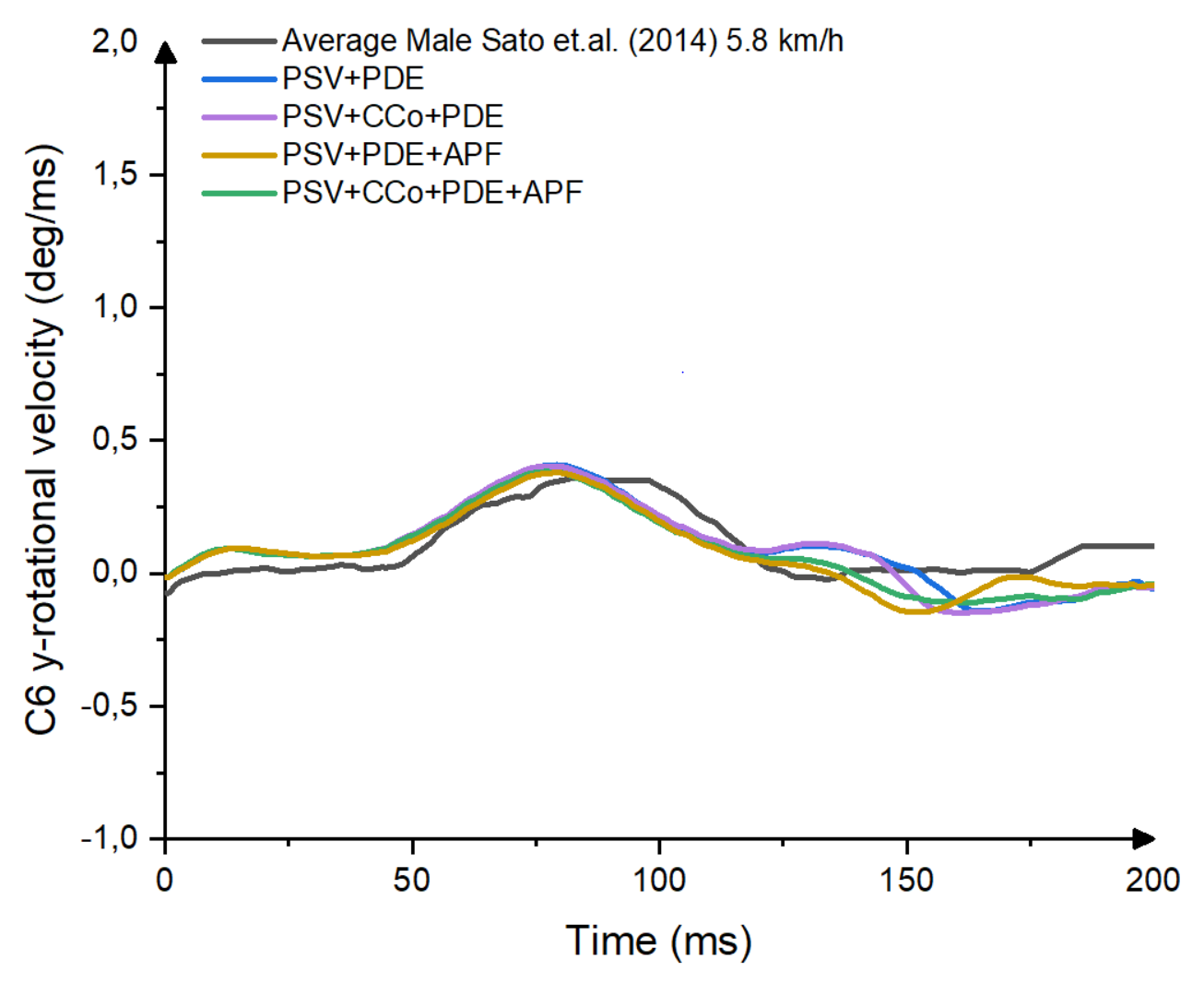


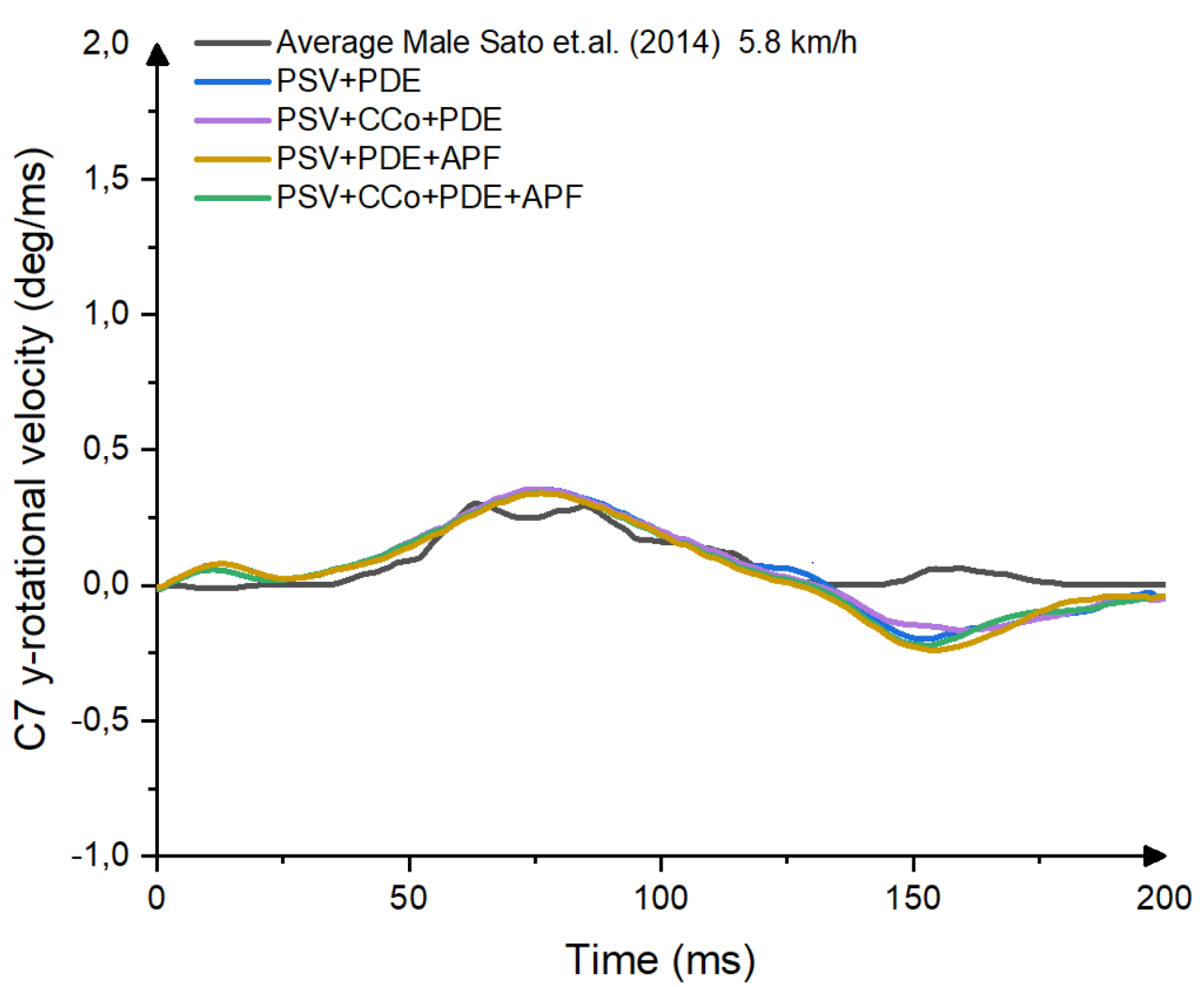


**Supplementary Figure 5.** Comparison of Cervical Vertebral Rotational y-Velocity between Male Models with Various Complexities and Volunteer Kinematics from Sato et al. (2014) 5.8km/h


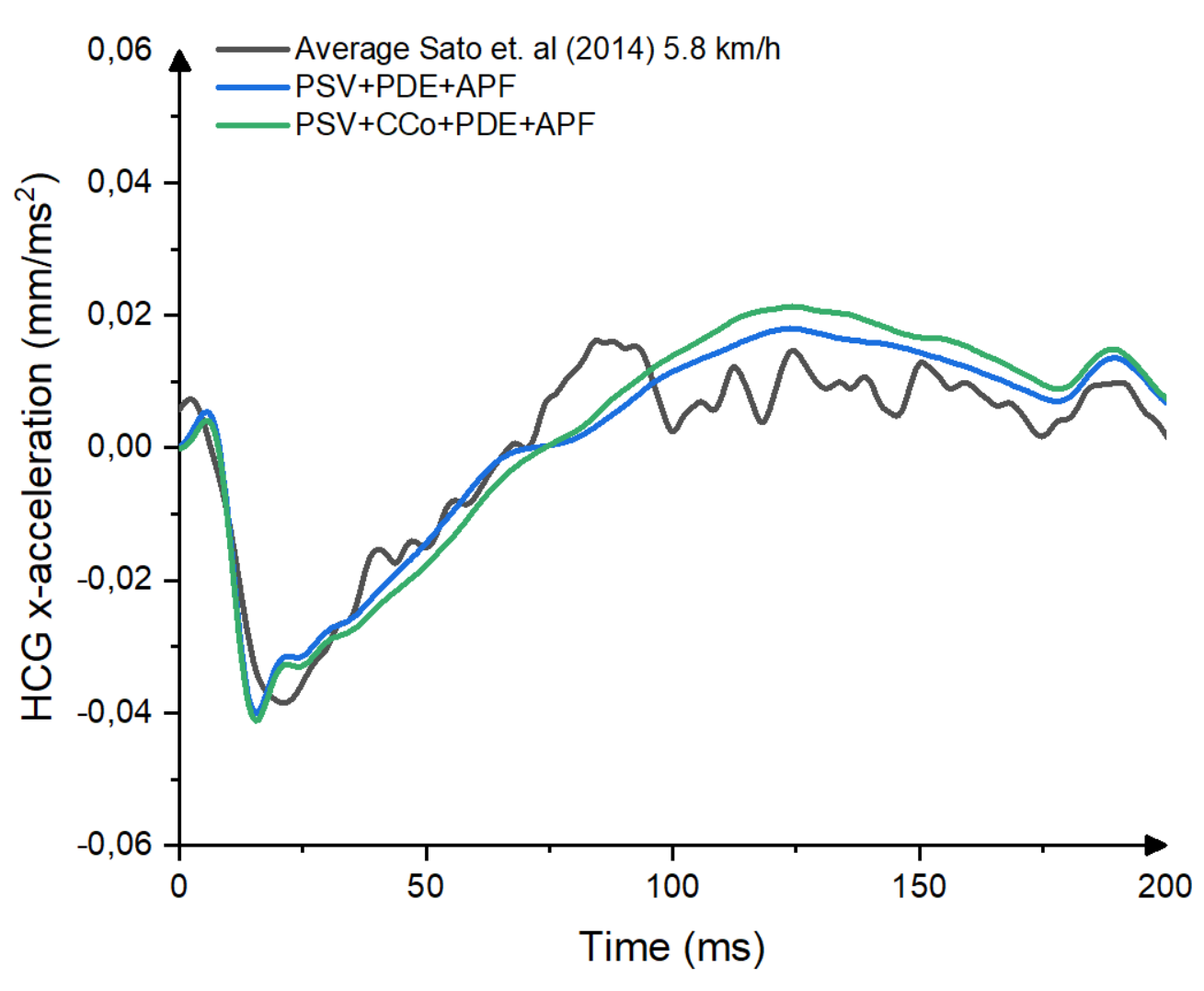

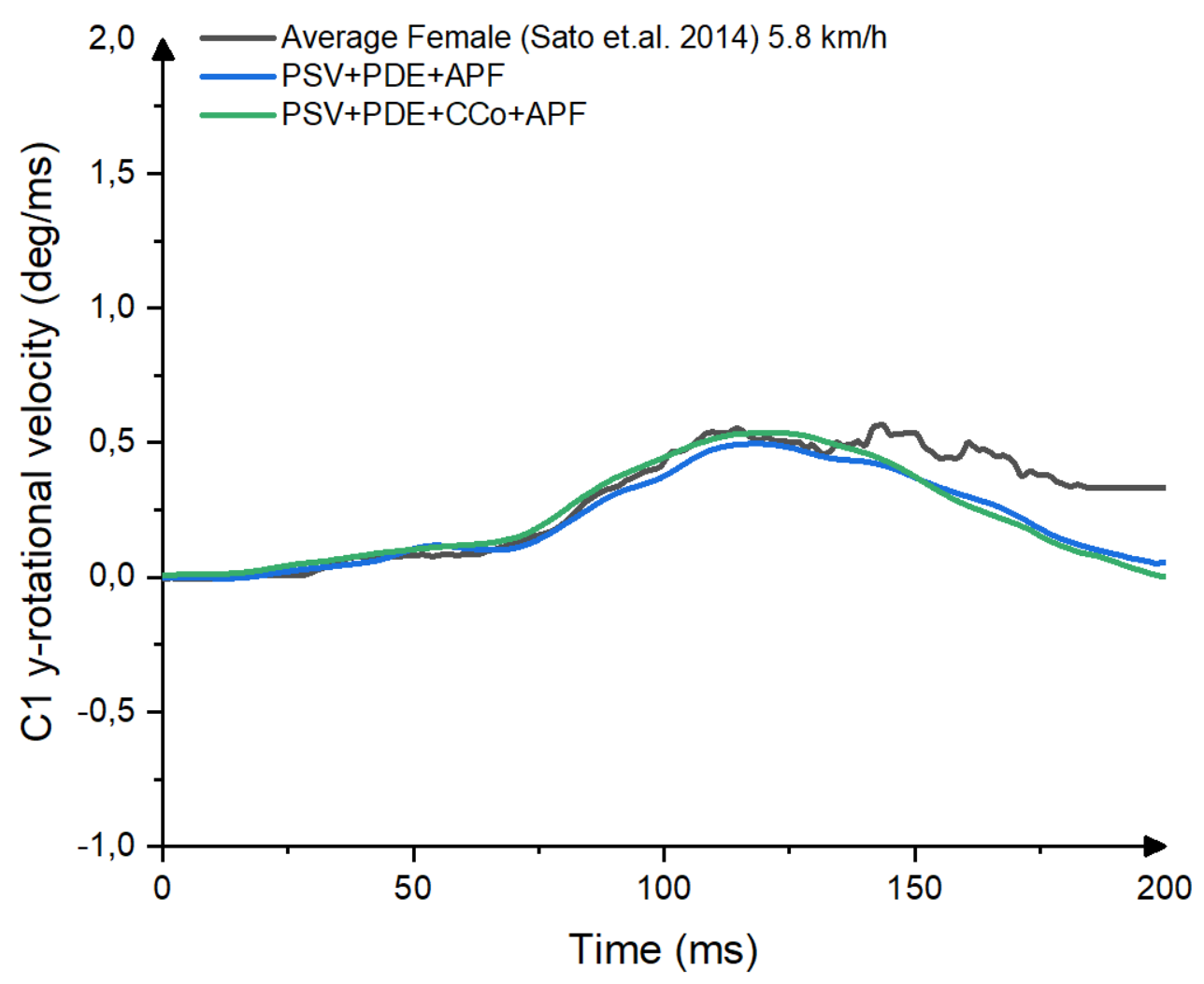

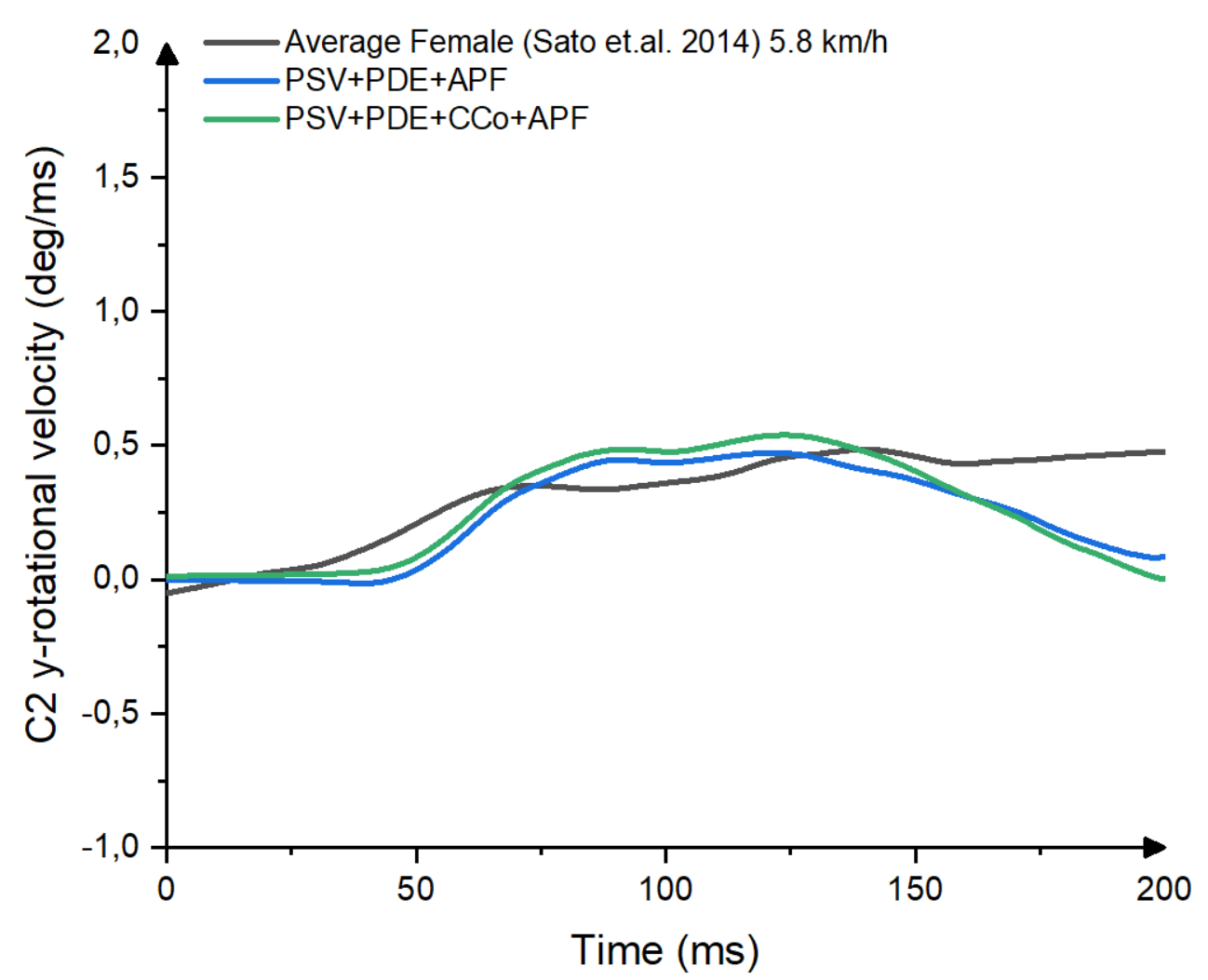


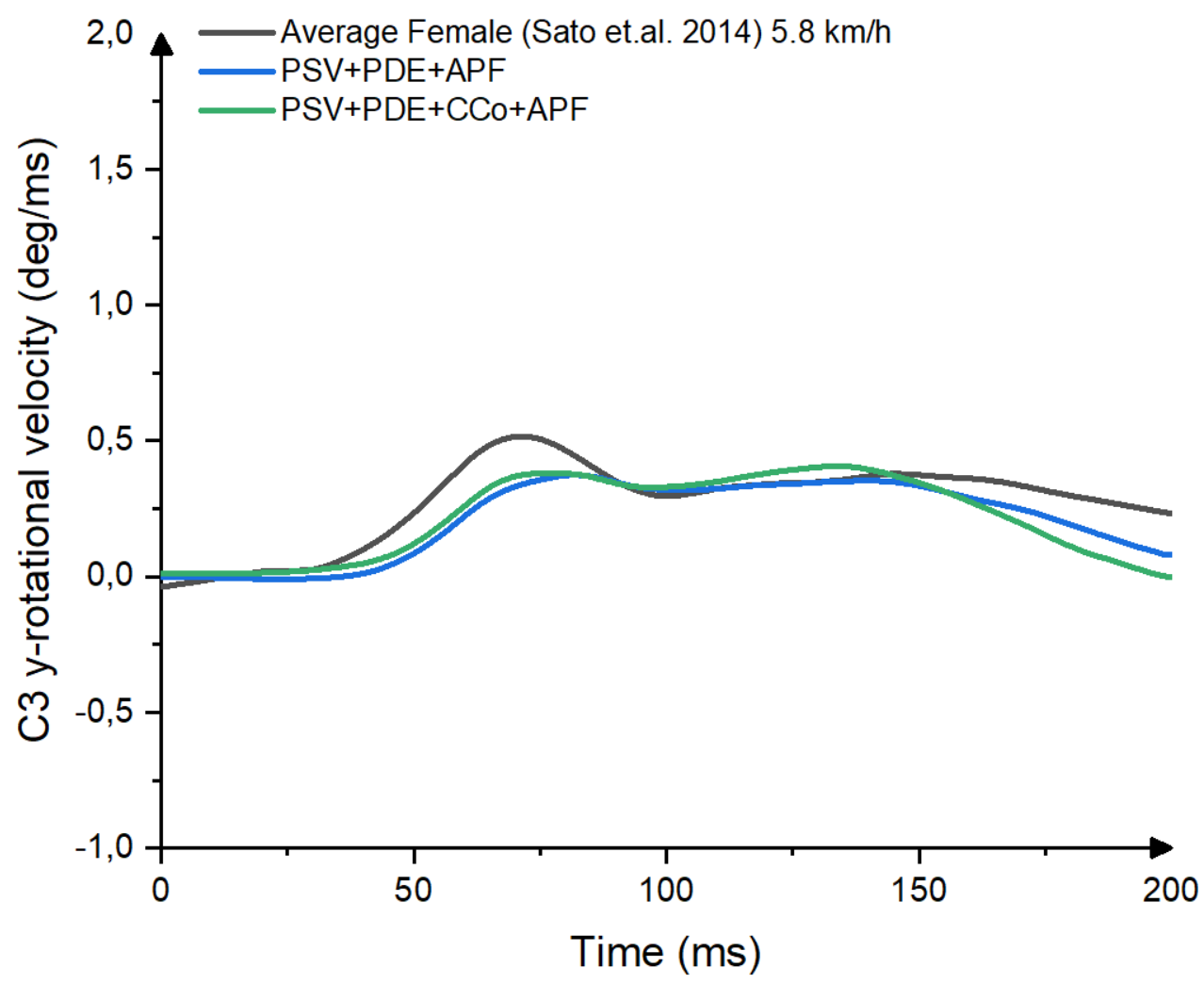

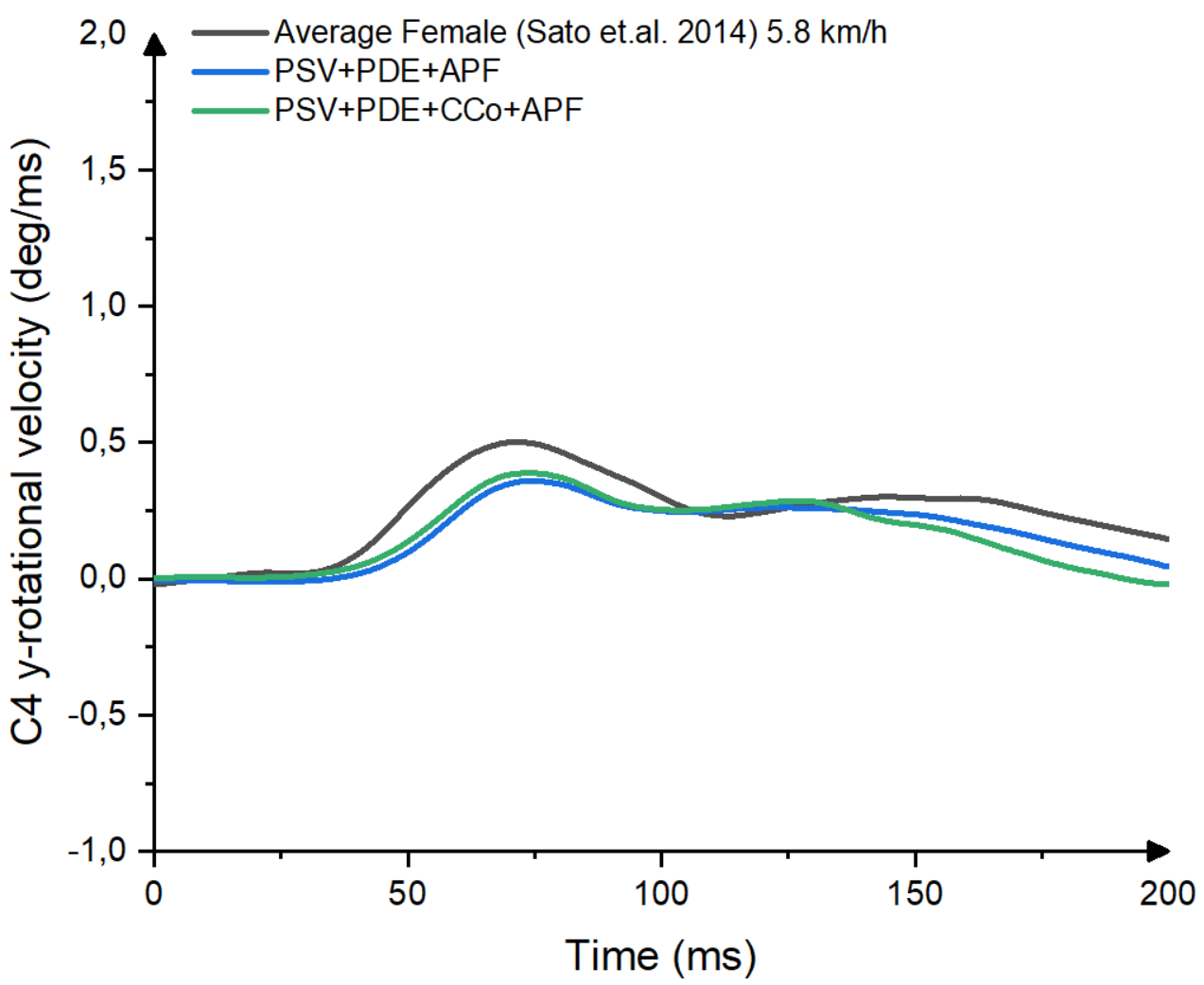

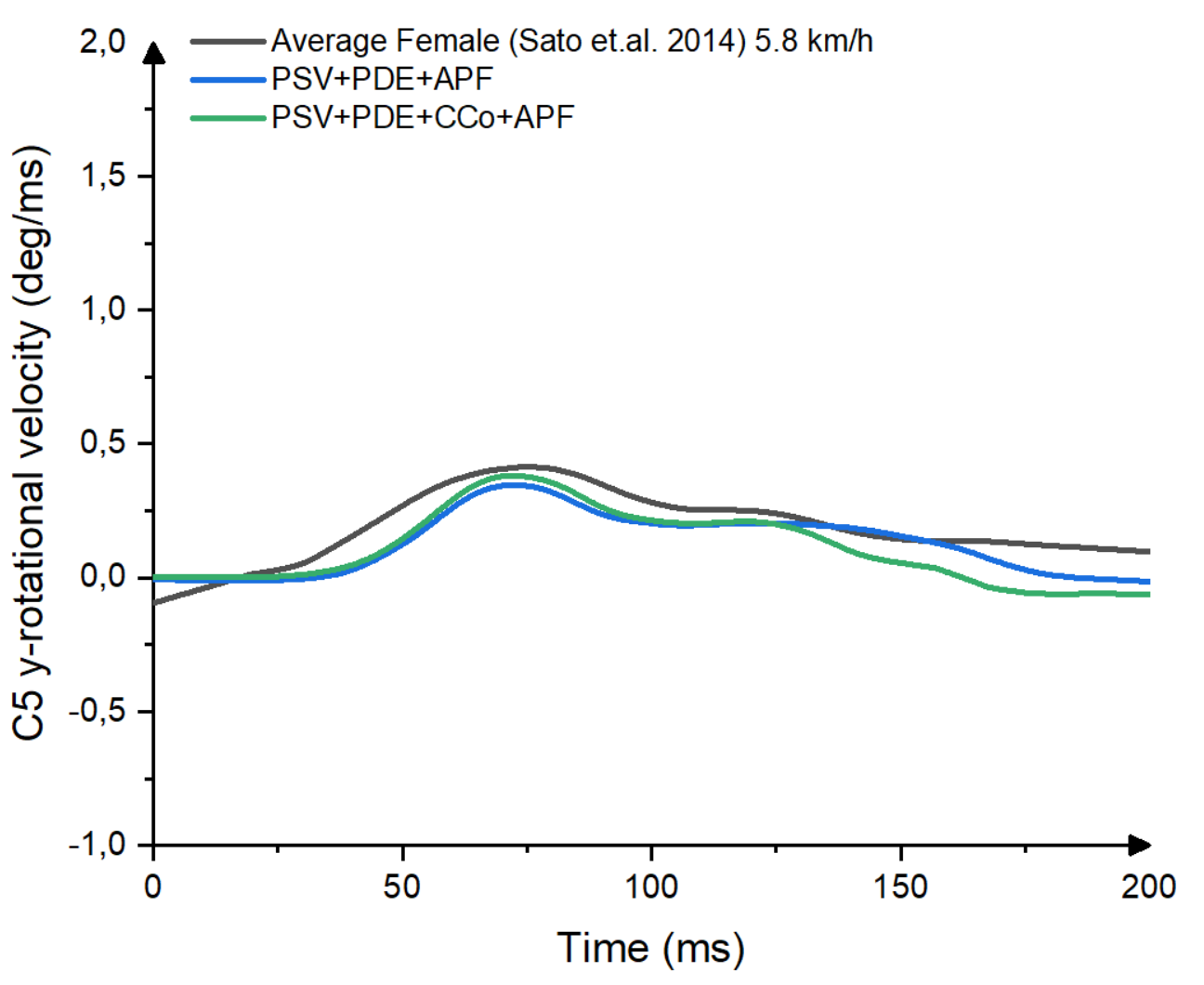


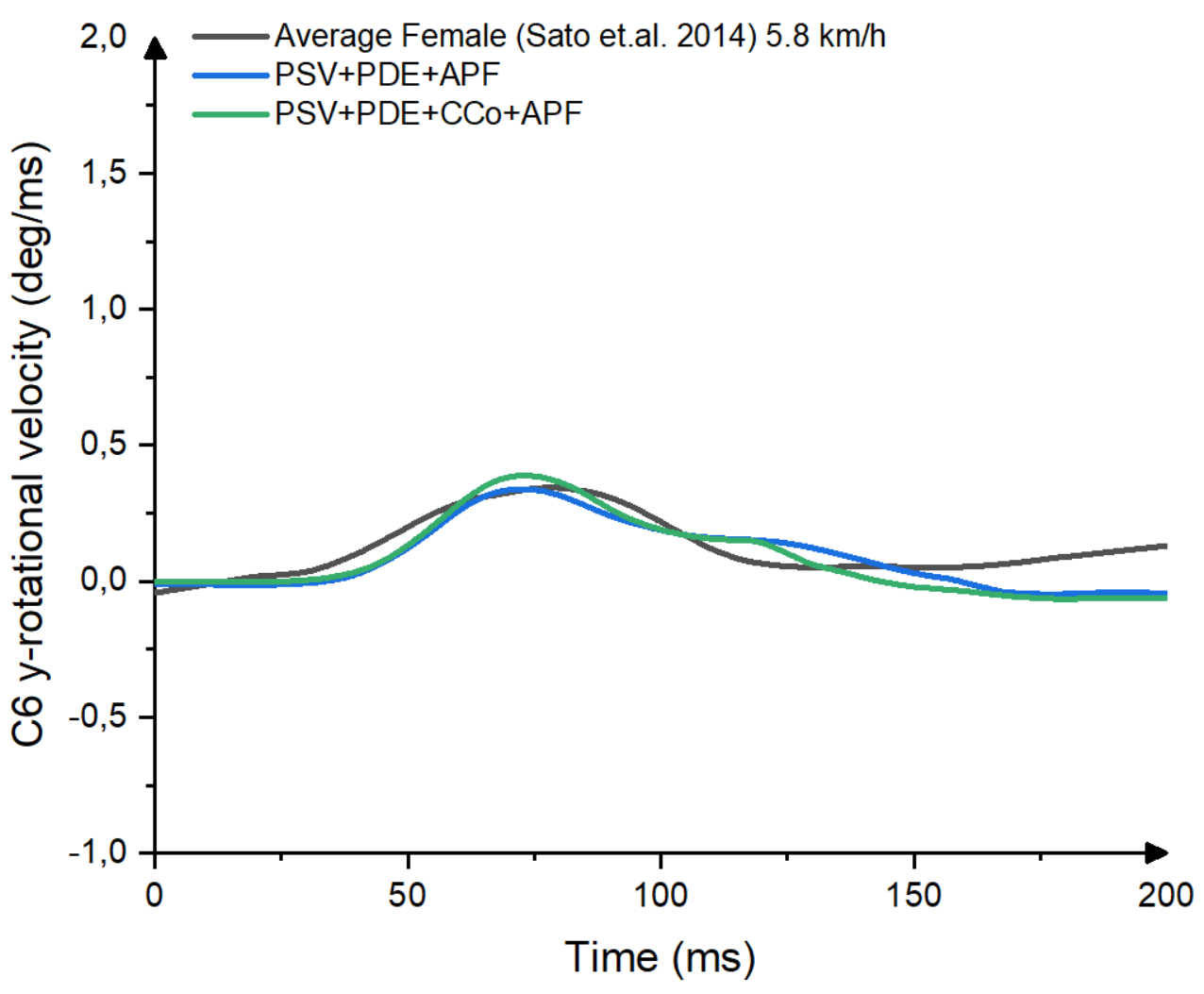

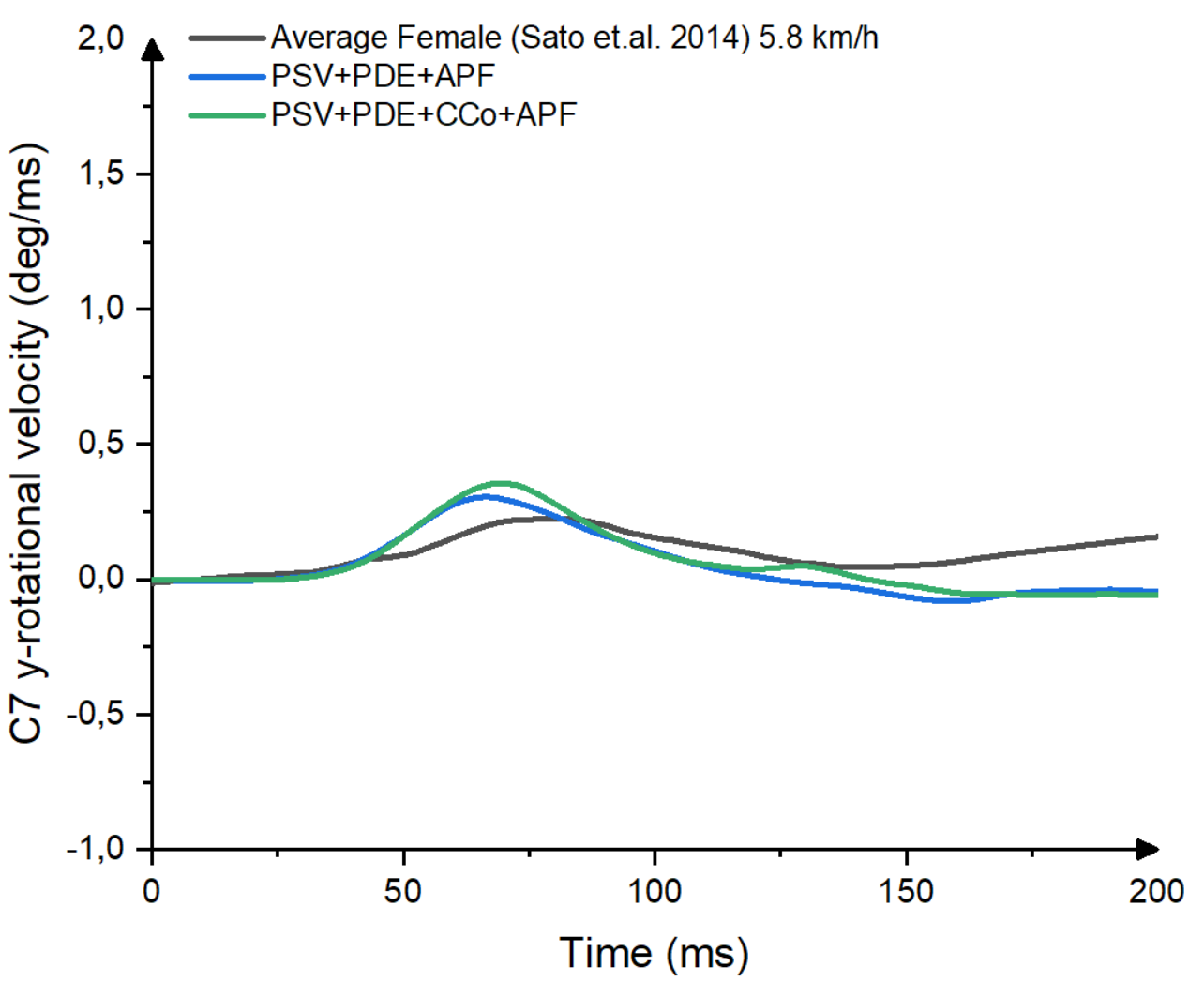

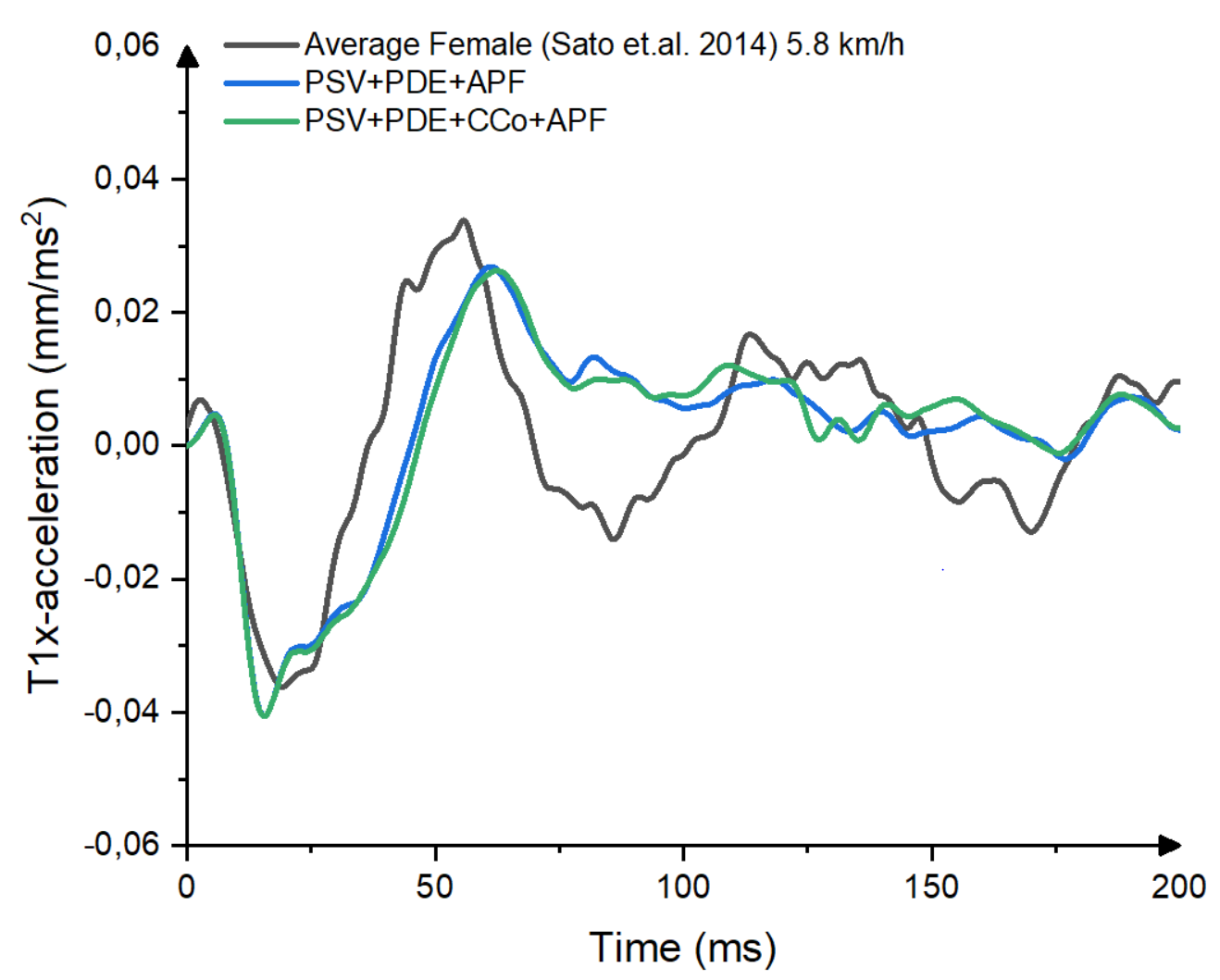


**Supplementary Figure 6.** Comparison of Head C.G x-Acceleration, T1 C.G x-Acceleration and Cervical Vertebral Rotational y-Velocity between Female Full-Body Models with Various Complexities and Volunteer Kinematics from Sato et al. (2014) 5.8km/h


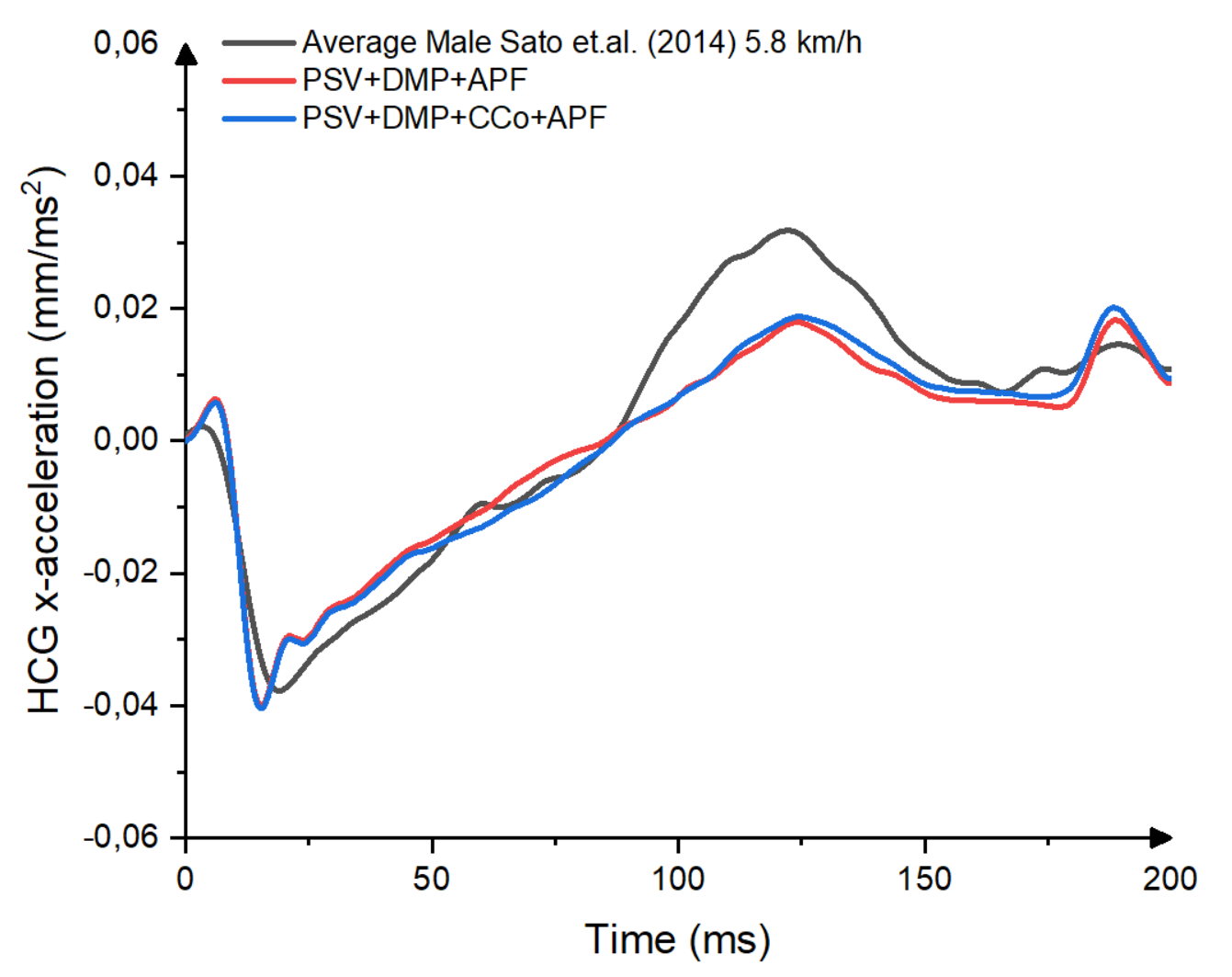

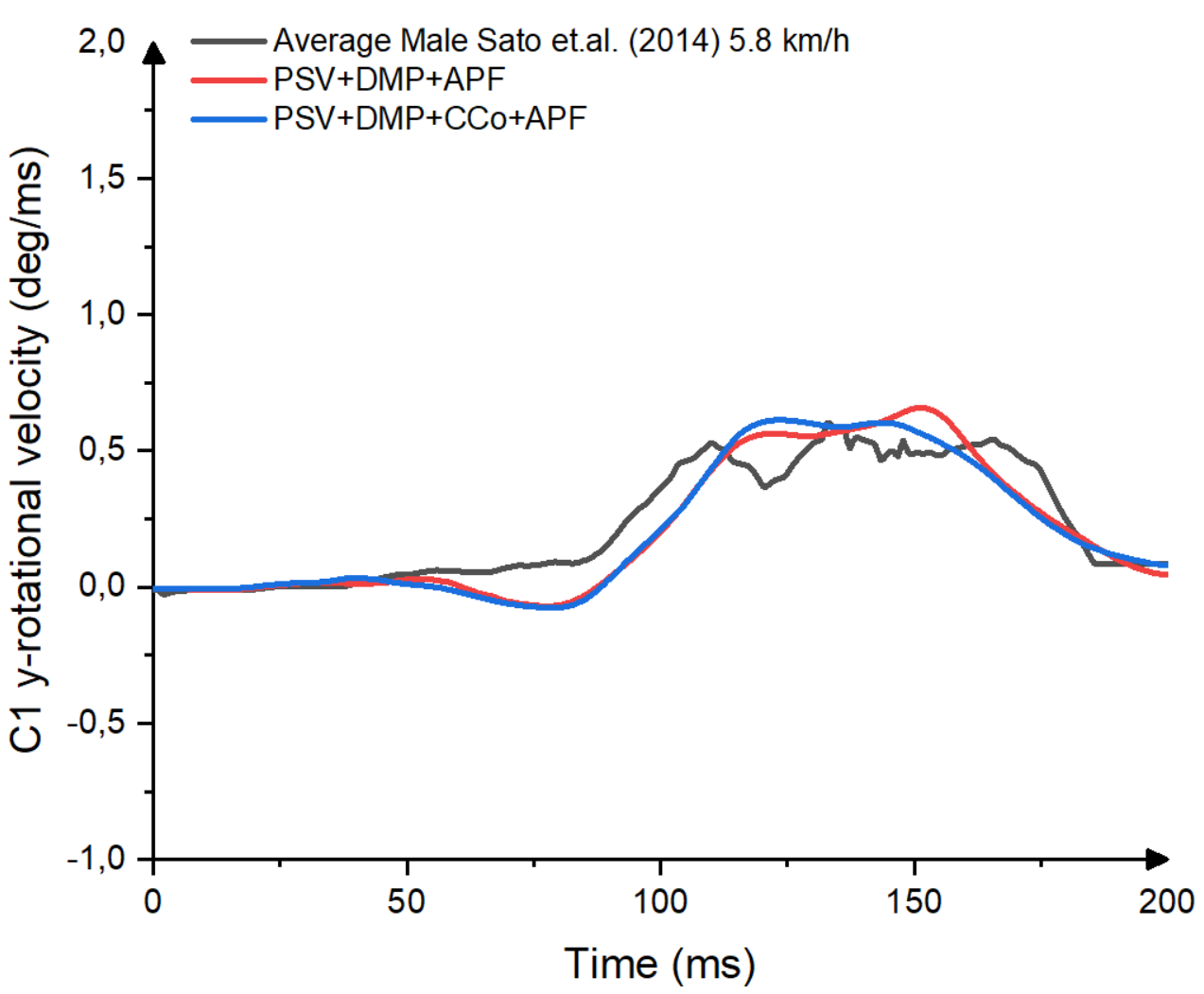

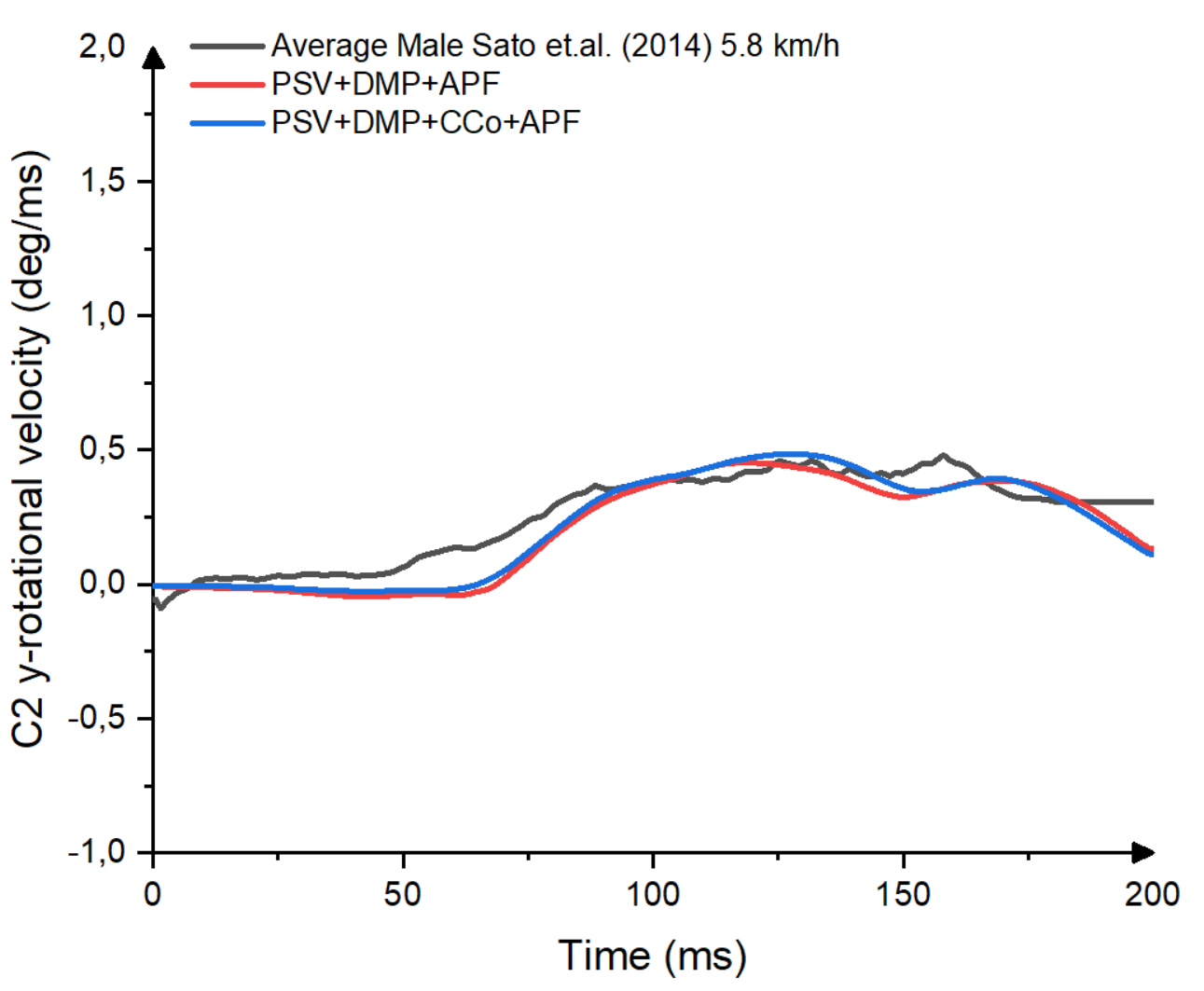


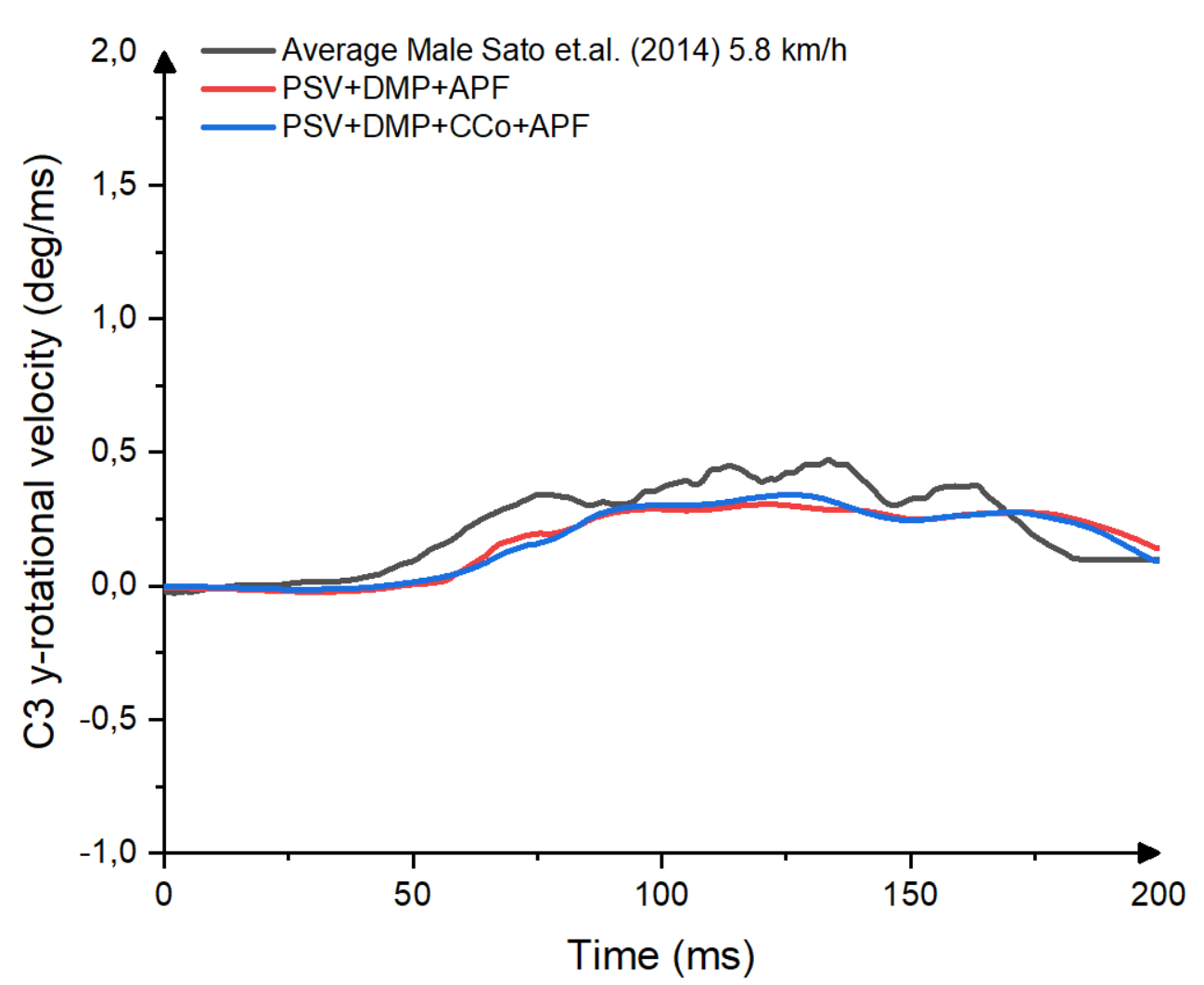

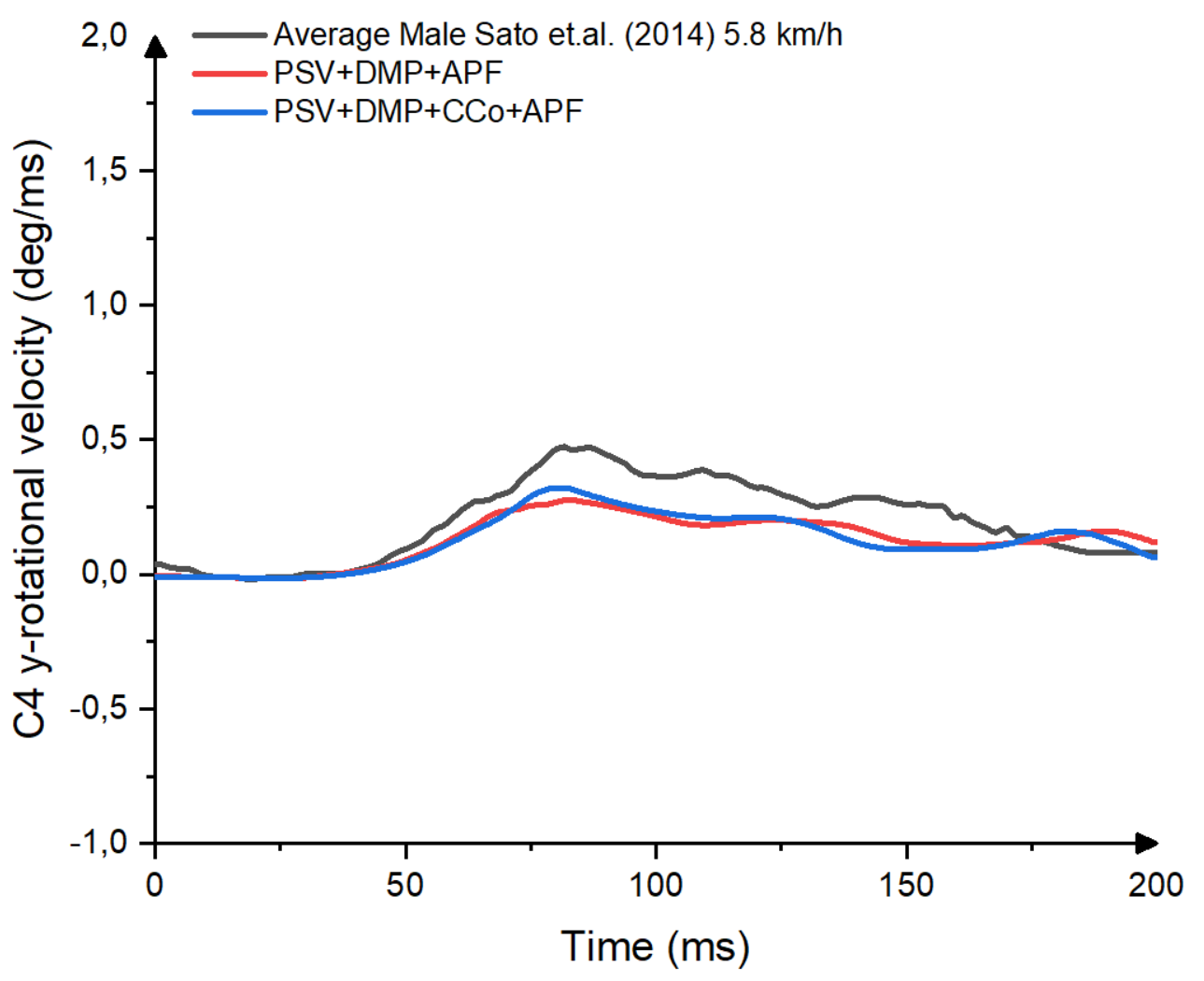

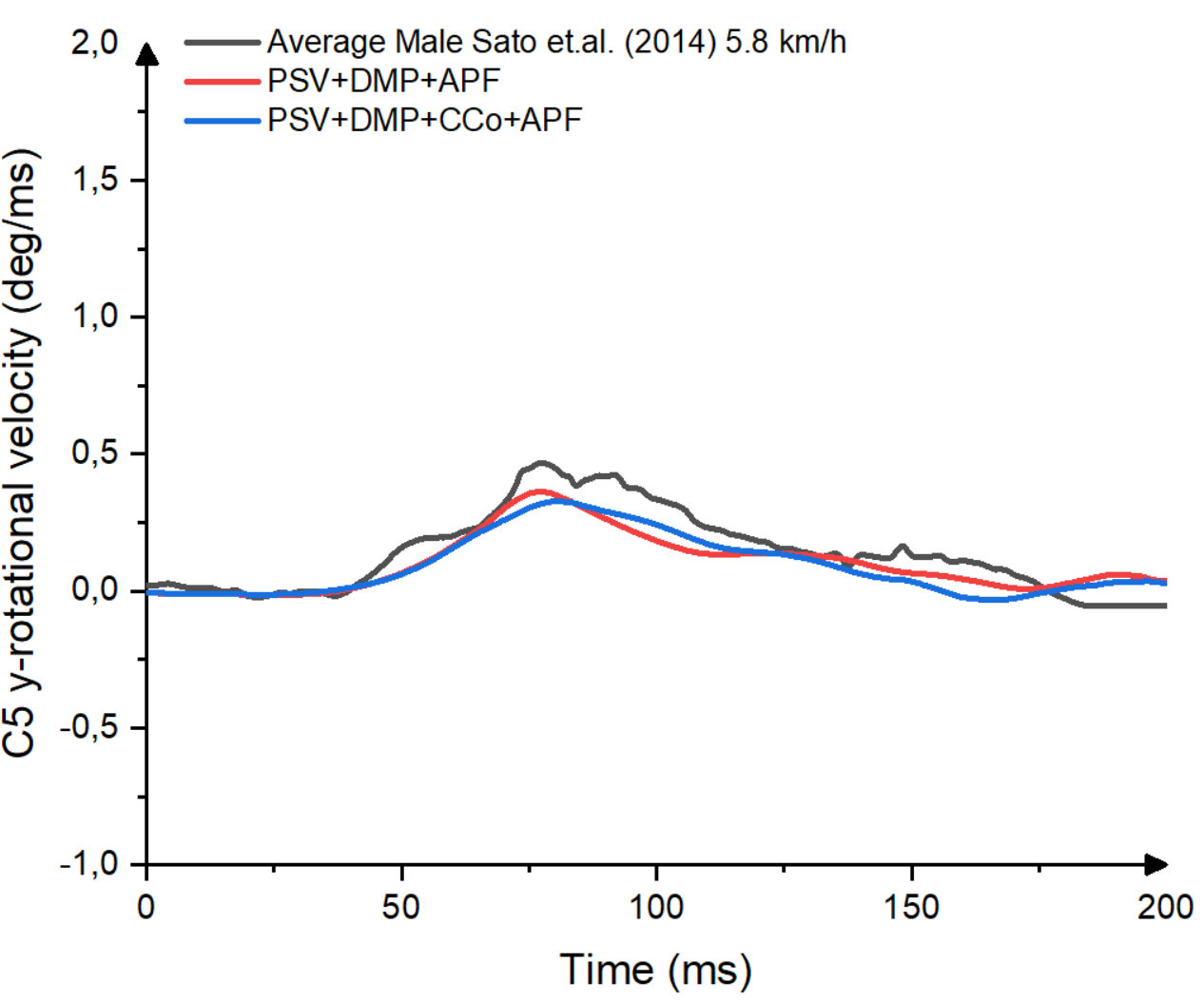


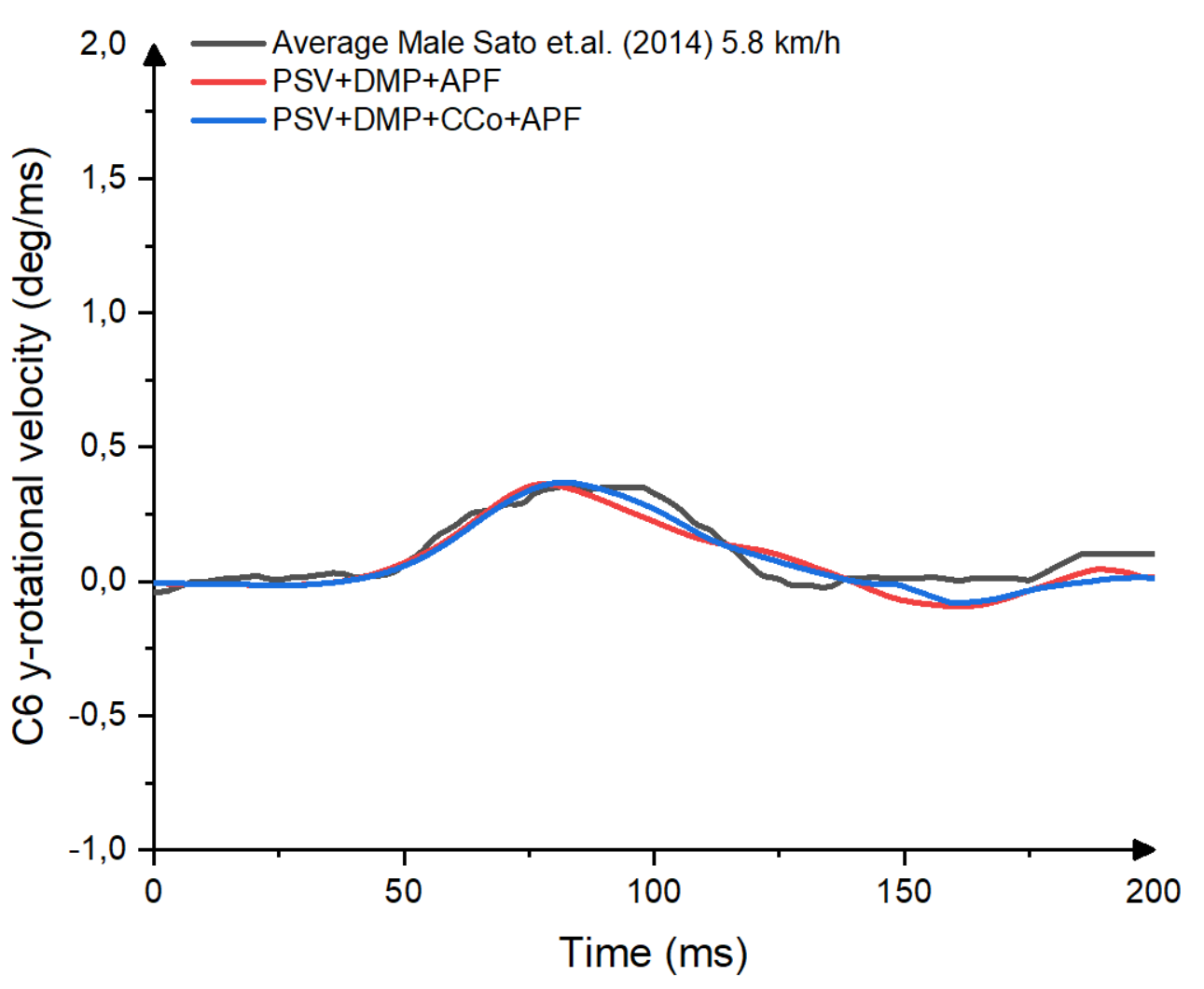

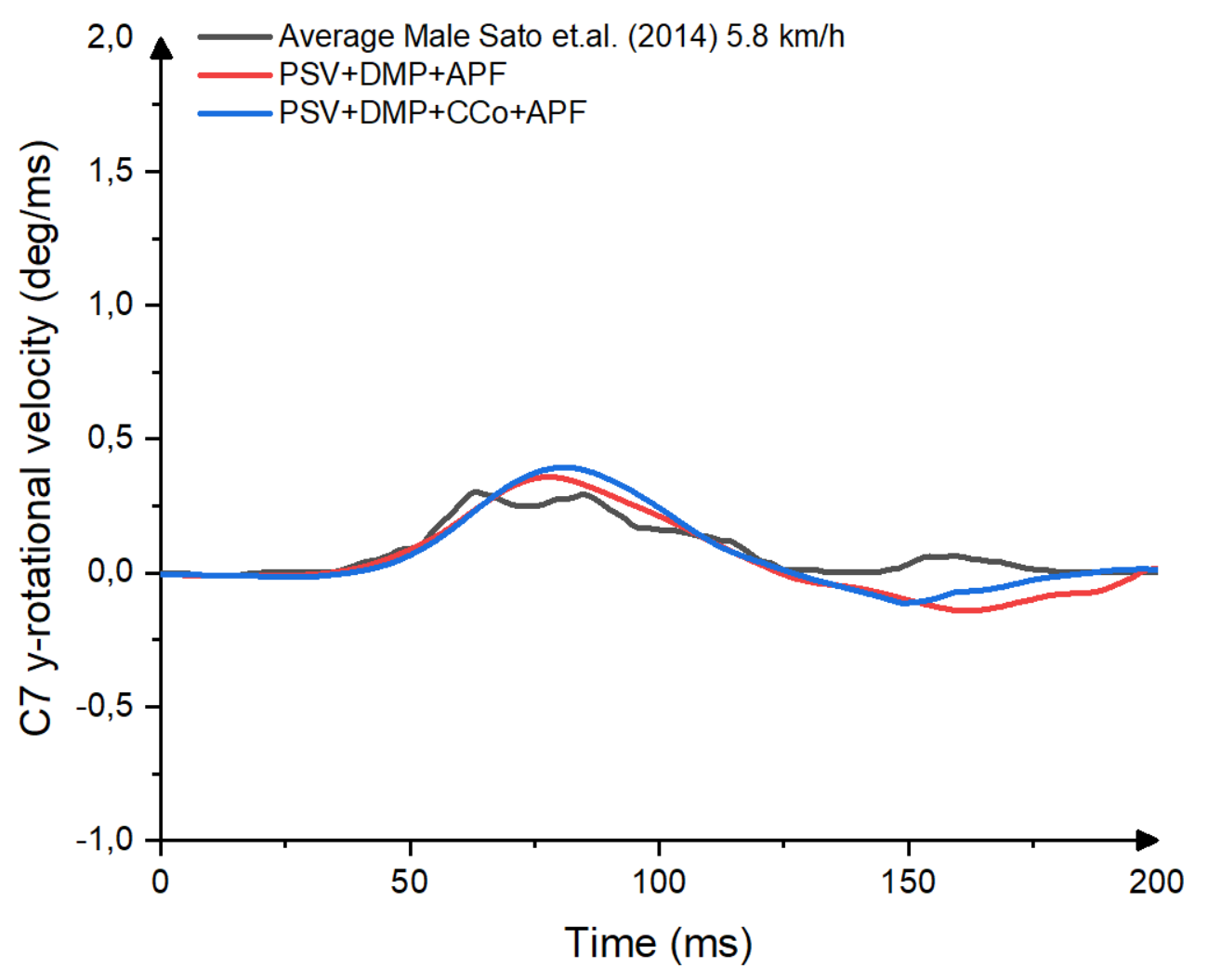
 **
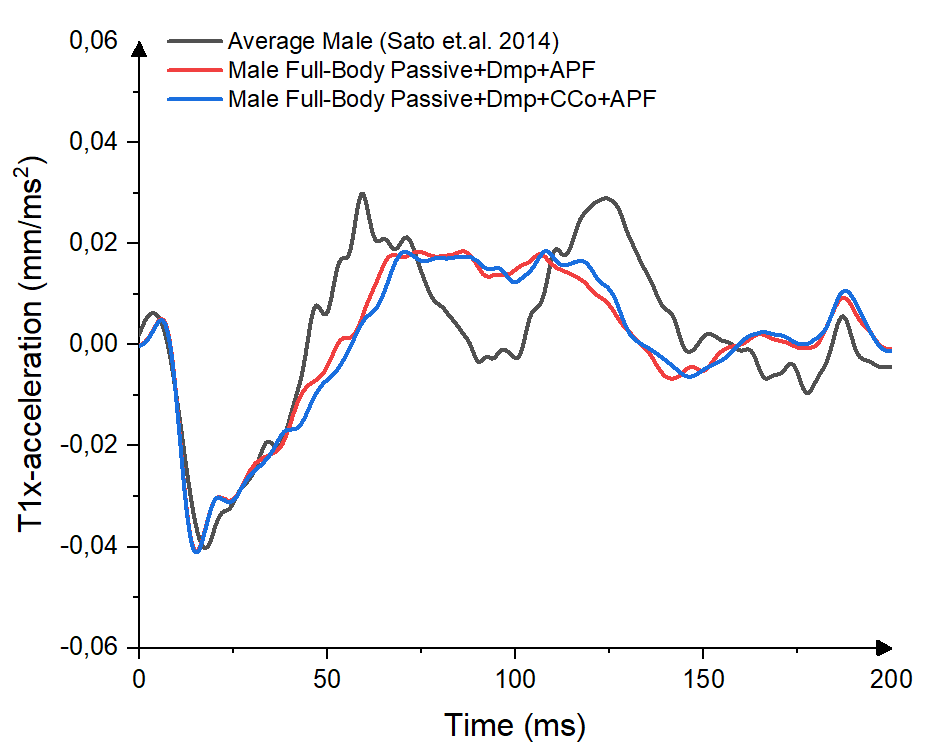
**

**Supplementary Figure 7.** Comparison of Head C.G x-Acceleration, T1 C.G x-Acceleration and Cervical Vertebral Rotational y-Velocity between Male Full-Body Models with Various Complexities and Volunteer Kinematics from Sato et al. (2014) 5.8km/h
